# Supplementary material for: Towards a Feminist Global Health Policy: Power, intersectionality, and transformation
Source: PLOS Glob Public Health. 2024 Mar 7;4(3):e0002959. doi: 10.1371/journal.pgph.0002959 (PMC10919653; doi:10.1371/journal.pgph.0002959)
Supplement: S1 Appendix — (PDF) [file pgph.0002959.s001.pdf]

Annex S1: Guiding questions for facilitating the focus group discussions:

Question	Annotations / possible follow-up questions for FG1	Annotations / possible follow-up questions for FG2 & FG3
**1. *Identification of components (What)***		
Question for everyone:<br>• **What are the most pressing structural challenges regarding global health policy?**	• Identification of problems*:*<br>→ structural perspective, causes of inequalities, rooted in society, to be influenced by policy<br>→ distribution of power; intersecting inequalities; structurally manifested (e.g. racism, sexism, classism)<br>→ possible examples	
• **What alternatives/solutions can a feminist global health policy provide?**	➔ Get into discussion, add to components mentioned by the others, or add new components, you can start thinking about Who & How already (we will also come back to that later)	
• **What are key components of a feminist global health policy?**	• Facilitating and con-straining aspects?<br>• What needs to be considered?<br>• What is already there?	• Facilitating and constraining aspects?<br>• What can a global framework encompass? (keyword: universal application, but local adaptions)
2. Identification of actors (Who)		
• **Who holds power and whose interests are being served?**	• Status quo<br>• Reference to relevant actors in Global Health: WHO, World Bank, Gates Found-ation (and other PPPs) … Remarks/ Evaluation?<br>• Structural aspects: Leadership, Finan-cing, Accountability → coloniality<br>• Possible reference to today's development	• Keyword: decolonial lens<br>• Whose interests are being served? → interests behind power<br>• Structural aspects: Leadership, Financing, Accountability → coloniality

	(anti-choice, right wing etc.)	
• **What are necessary steps to transfer power and to whom?**	• Whose interests should be served?<br>• Challenges?<br>• Agency/Empowerment → Civil society<br>• Political responsibility, but strong social impact	
		• Reference to relevant actors (on local level OR impact on local level from global level)
• **How can power regimes be dismantled?**	• At global level	• At local level
	• Mutuality, participation, decoloniality<br>• Intersectionality<br>• Terminate structural discrimination<br>• Start with most affected first<br>• Social change equally important	
• **Accountability: Who is responsible for adopting / implementing a feminist global health policy?**	• Global scope	• Local scope
	• What is needed to ensure/enable that?<br>• What / whose interests? → very heterogenous<br>• Partnerships / Alliances?<br>• Transformation without polarisation, to attract everyone	
3. Identification of action (How)		
• **How can activists / social movements / civil society be included?**	• Facilitating and constraining aspects? Challenges?<br>• What is their role?<br>• What is already there? → What can be built on?<br>• How can a global movement for a feminist global health policy emerge?	
• **What are concrete political steps towards a feminist global health policy?**	• Facilitating and constraining aspects?<br>What is already there? / What can be built on?	
• **How to implement a holistic, intersectional framework?**	• At global level<br>• First step / preconditions?<br>• On what grounds/ progress so far? Anything to build on?<br>• Challenges?<br>• Considering intersectionality	• At local level<br>• Facilitating and constraining aspects? Challenges?<br>• What is already there? → What can be built on?

		• Reference to past movements? → What to learn / make better?
• **How can social norms (i.e. gender norms and other harmful presumptions) be changed?**		• Local context → access to society
	• Underlying problems: structural discrimination / roots of inequalities not always obvious (esp. not for those not directly affected), reproduced by society → how to change / break the cycle?<br>• Political importance for social change? Sensitivity?<br>• Who?	
• **How can intersectionality be incorporated in concrete action?**		
• **How to transfer the message that a FGHP is beneficial for everyone?**	• Considering that a transformative FGHP is meant to address and ideally be beneficial for everyone - this encompasses quite a heterogenous group of people with various interests → How to combine/pool all the interests to achieve a global change? (Keyword: Intersectionality)	
Last questions		
• **Did we miss anything important / Is there anything you like to add that we haven't mentioned so far?**		
• **Your outlook/ hope/wish/dream regarding FGHP?**		
