# Supplementary material for: Towards a Feminist Global Health Policy: Power, intersectionality, and transformation
Source: PLOS Glob Public Health. 2024 Mar 7;4(3):e0002959. doi: 10.1371/journal.pgph.0002959 (PMC10919653; doi:10.1371/journal.pgph.0002959)
Supplement: S2 Appendix — (PDF) [file pgph.0002959.s002.pdf]

Transcript of FG1

Date of the focus group: 23 August 2022, 13:00 CET

Duration: 119min 57sec

Place: Online via the audio-visual tool Zoom

Moderator: **HE**

Participants: **CJ, ER, JC, MM, SKY, SE**

Transcription notation

**(…)**	Break up to 3 seconds
**(number)**	Break longer than 3 seconds, duration in brackets
**underlined**	Particular emphasis
**(nonverbal)**	Nonverbal expression
**(unint.)**	Unintelligible speech
**(word?)**	Unintelligible, assumed speech
**ehm**	Uniform notation of filler words (ehm, ah, eh)
**/**	Interruption of word or sentence
**//**	Speech overlaps

Transcript

1	[0:00:00.0]
2	[0:05:00.3] **HE**: Hello.
3	[0:05:02.1] **SKY:** Hi. (...)
4	**HE:** Just waiting until everyone arrives.
5	(24)
6	[0:05:34.1] **HE**: Hello, hi SKY, hi JC!
7	**SKY**: Hi HE!
8	(HE smiles, JC waives)
9	(4)
10	**JC**: Hello.
11	[0:05:45.2] **SKY:** Going to start my video. Hi. (SKY waives)
12	**HE**: Great, thank you. Hi. Ehm I'm just waiting //

// **SKY**: I am fine, how are you?

[0:05:52.8] **HE**: I am really good. Thank you. How are you?

SKY: I'm fine. Thank you.

HE: Nice. Yes, yeah, Thank you all for being here, especially also JC, for joining so early and CJ for you it's quite late (laughs). (Everyone smiles) (6) I'm just waiting for three more people to join. (26)

[0:06:43.5] **HE**: Hello, Hi, MM!

[0:06:46.2] **MM**: Hi HE.

[0:06:48.5] **HE**: How are you?

[0:06:50.2] **MM**: I'm good, and you?

HE: Thank you. Yeah, I'm also good.

MM: Great. (...) Hi everyone. //

// **HE:** I think everyone (6)

[0:07:05.1] **HE**: Yeah everyone's joining, now. (5) Hi ER. (5)

[0:07:17.9] **ER**: Hello! Sorry I'm late.

[0:07:19.7] **HE**: No, don't worry. So (...) yes, we're almost all there, (7) just waiting until everyone's settled. Hi SE.

SE: Hello.

[0:07:41.3] **HE** Okay, Great. So we're everyone now (...) yes.

[0:07:49.3] **HE**: Okay, great. Then I ehm would like to start. First of all, hello and welcome to all of you. I'm really really glad that you're all here and ehm that you're willing to partici/participate in this focus group discussion ehm thank you really, I know I'm asking a lot from you, and that it's very early and very late for some of you. Ehm I'm very excited for the discussion. Ehm, so yes, thank you. Ehm, before we start, I just want to quickly talk about some housekeeping. Ehm, as you notice the Zoom recording ehm already started, it's just so that I don't miss anything and it was easier to set it up this way. Regarding the formalities, I sent you everything from my university ehm about the procedure about data security. So ehm you all sent me back the signed form, so I assume you know about it. But if you have any objections, then you can tell me now. (...) Okay, great ehm. Then I'm happy to see that you all turned on your video, and I invite you to keep it on during the session as far as it's possible. I just think it's nice if we see who we're talking to, and it creates more of a familiar atmosphere. Ehm, but of course, if

your internet connection is not stable enough, then you can turn your video off. Ehm then I would also ask you to try, at least, not to talk at the same time too much, or to interrupt each other too much, also because I have to transcribe the session. Ehm, but it's meant to be a free discussion. So If you want to add something, if you want to jump in, then you can do so. So you don't have to wait for me to give you permission to talk. I'd rather want it to be a natural conversation. And I think with six people this will work. Ehm, so maybe you can even consider to leave your microphone on if we're in the discussion if you don't have too much background noise, we have to see how this works. Ehm yeah. So that's it for the technical stuff.

[0:09:55.8] Ehm, then I think I should also introduce myself because I only had e-mail contact to all of you before. So, as you know my name is HE. I am 25 years old. My pronouns are she and her. Ehm I live in Berlin in Germany, and I study the masters Public Health and Political Science at the University of Bielefeld. And I study these two masters because I'm especially interested in the intersection which is global health policy. I also worked with some NGOs here in Germany, in the field of global health and right now I'm part of a university project on Decolonisation of global health. Ehm more personally, I'm a sportive person. I like dancing or going for a run, ehm I also enjoy very much living in Berlin with my boyfriend, just to have this offer of everything around me. (CJ, JC and MM smile) Ehm, yeah, so that's me in a nutshell (laughs). Now I would also love to get to know you a little bit more, ehm and I don't want to be talking all the time. So I suggest we make this quick round of introduction, and everyone just introduced themselves. So we have a bit more interaction. Ehm and yeah, so maybe, I see on my screen ehm MM, if you want to start ehm then feel free to do so.

[0:11:19.5] **MM:** Hi, ehm hello everyone. I hope I don't have too much background noise. I'm at the yeah public library today so just let me know if it's too much. Ehm so my name is MM, my pronouns are she and they. Ehm I'm a social scientist, so trained mainly in anthropology and sociology. Ehm I'm originally from France, but I studied yeah I've done my PhD studies in Canada. I also worked in the UK and now I'm working in Belgium. Ehm (...) Yes, I've worked mostly like on health inequities, discrimination ehm yeah in my whole life. So I'm coming from a research background more. Ok, that's it from me, I can pass the mic. Thank you all. (HE smiles)

[0:12:08.0] **HE:** (...) Thank you. Ehm, maybe just CJ, you go next.

CJ: Ehm, sure. Hi, hi everyone I'm CJ or in the e-mail. Ehm I'm with UN Women in the Philippines. I'm handling the Ending violence against women portfolio and ehm the most ehm, I guess relevant and ehm recent ehm initiative that, I am particularly involved in is really on the intersection of violence against women and ehm and health, with particular attention, of course, to sexual and reproductive health and mental health or psychosocial health, so ehm VAW or GBV survivors. And yeah, looking forward to the discussion. (HE smiles)

[0:12:58.8] **HE:** Great. Thank you. Ehm SE, you want to go next?

[0:13:02.4] **SE:** Hi everyone. I'm SE, I'm from Egypt ehm, right now I'm working with the Swiss Tropical Public Health Institute. Ehm. My work is actually on diverse areas but mainly around sexual reproductive health ehm health financing and some of my work focus on the gender equality, the intersectionality, access to healthcare to women and intersectional groups. Ehm and I am affiliated also with the Women in Global Health in Cairo and we work on gender representation in many sections of health policy makers / representation as health policy-makers. Ehm, that's about it. (laughs)

[0:13:55.6] **HE:** Thank you. Ehm, JC, you want to introduce yourself?

[0:14:00.5] **JC:** Sure. Hello everyone. Nice to meet you. My name is JC. I use he/him pronouns. I'm a human rights lawyer and a professor based in New York, ehm originally from Canada. Ehm and after my legal training worked for nearly two decades in the non-profit and philanthropic sector, at organisations like Human Rights Watch and the Open Society Foundations, working at the intersection of health and human rights. Ehm my life's work is really about the application of the International Human Rights framework to public health and global health. And I'm currently appointed as a clinical professor at the University of Southern California, at our Institute on Inequalities in Global Health, where I direct the Institute's policy engagement work. Very nice to meet you all. Thanks.

[0:15:05.7] **HE**: Thank you. (smiles) ER, you want to go next?

[0:15:13.5] **ER**: (...) Was that me by any chance because my microphon/ my hearing, just cut out. (laughs) Sorry about that. Hello, my name is ER. I work at the United Nations University's International Institute for Global Health, ehm which is the UN Health Policy Think Tank. And I work, I'm a senior researcher in the team focused on the translation of evidence to policy. I also lead the Secretariat for the Lancet Commission on Gender and Global Health, and and as part of that have been developing and implementing a decolonial

feminist approach ehm to engagement with stakeholders ehm who are implementing work at the intersection of gender and global health. Ehm using that decolonial feminist approach, so really looking forward to the conversation and it's lovely to meet you.

[0:16:03.8] **HE:** Thank you. (smiles) And then last, but not least, SKY.

[0:16:08.5] **SKY:** Hi everyone. My name is SKY and I am based in India in Delhi. Currently, I'm working with the George Institute for Global Health and reading a program which is on mental health of adolescence, living in that two/ in the slums actually, in two Indian cities. But before this I used to work in an organisation called Sahayog, that was based in Northern India, and ehm as part of that I did a lot of work around women, women empowerment ehm engaging with men to, you know, ehm reduce violence against women, things like that. And I look forward to the discussion. Great to be here. Over to you HE.

[0:16:52.5] **HE**: (Thank you?) I can only repeat, I'm really happy that you're all here today, and I'm looking very much forward to the discussion. So just to remind us why we're all here, just as a quick refresher, I brought a very short power-point presentation, but it's mainly the aspect I also sent you in the concept note. So I would just share my screen with you. (...) One second.

[0:17:21.4] (HE shares her screen and opens the power-point presentation)

HE: So now you should all see the presentation. So, as you know, this project is part of my master thesis on the topic of Feminist global health policy – addressing health inequalities through an intersectional perspective. (HE CJes to the next slide) And this project is based on an intersectional approach, and I focus on the structural determinants of health, mainly gender, race and class. And I based this on the WHO framework on the social determinants of health. And this framework emphasises the political context and also the role of power regimes and how these cause structural inequalities and discrimination. And in my thesis I also give examples of how this discrimination impacts the health and well-being. And I give examples on the levels you can see there ehm I focus a lot on gender inequality, but I also consider racism and classism throughout. So if you want, you can also refer to some examples during the discussion and bring forward your own examples. But of course, it's not necessary, it's just sometimes nice to specify some aspects. (HE CJes to the next slide) And I also brought you my understanding of a feminist global health policy, but this is based on the literature, so maybe we end with a totally new definition. And if you have a different

understanding, of course then share your thoughts with us in the discussion. But for me, now, the a feminist global health policy is a holistic and intersectional approach which means it should also be inherently decolonial. It focuses on the socioeconomic-political level and it aims to challenge and also to CJe power hierarchies and the resulting discrimination. And it does so by focusing on the most marginalised first, and by a shi/ by shifting to more participation. So, with my thesis I aim to create a framework on feminist global health policy and how it can be implemented. And I do so on the basis of these focus groups as far as this is possible, of course. (HE CJes to the next slide) And so for the discussion I would like you to to keep in mind that the underlying principles are intersectionality and the role of power regimes, and I have this very broad frame for the discussion, which is that we cover the aspects of what, who, and how. If we think about a feminist global health policy. Now, let me just stop that. (HE stops the presentation and screen-sharing)

[0:19:52.4] Ehm and then there's one more aspect which is very important to me, so I would like to mention that ehm because you know that I'm adapting feminist research methods, and I'm using the focus group so that I remain in the background and the focus in on you. But I also know that this is not a perfect research pro/ project. Ehm I am a white woman, I come from a Global North institution, so I'm a very privileged person. And of course I invite you all to use the results, and I really hope that you benefit from this interaction, from this discussion. But I know that the results primarily serve my master thesis. Ehm, so I try to be very reflexive about this, but I know that there will remain some kind of power hierarchies. And yeah, I just want to mention this, and also that I will consider it in my thesis. But yes, having said all that, ehm I would also like to start with the discussion, so that we have enough time for that. I prepared some guiding questions, but I also ehm mentioned that I want the focus to be on you. So maybe if we just consider the what, who, how aspects ehm we should be on track. I also want to stress that this is an open and non-judgmental so discussion, there are no right or wrong answers. I also don't intend to find final answers because sometimes it's of value for research if we find new questions. And also you can disagree with one another. Ehm, yes. So ideally I did most of the talking right now. I want to start ehm with the first question, I also send it to you. And for the first question I would like to have a round where everyone gives an answer and after that we have a more open discussion when then whenever you want to say something you just jump

in. So for the first round ehm I want to ask you what you consider the most pressing structural challenges regarding global health policy. And maybe you think of a lot of challenges, but that you really pick the the most pressing one ehm at the moment. So is there anyone who would like to start, otherwise I pick someone? (...)

[0:22:15.6] **CJ**: Ehm so and the question is, what's the most pressing ehm global health issue?

[0:22:21.3] **HE:** Yes, regarding yes, regarding the status quo at the moment. Yes.

[0:22:26.4] **CJ:** Yes, I think ehm worldwide, we've seen ehm really the regression and the backlash against ehm women's health, including of course sexual and reproductive health and bodily autonomy. And I think that ehm of course, from the perspective of ehm my/ well coming from my work in UN Women, in relation to the intersection of ehm structural inequalities. Ehm that, of course, ehm position gender inequalities as a as as an as an effect of that, but as well as ehm how globally the perspective amongst even ehm those in position in power which is, of course, male dominated ehm and conservative ehm and anti-women is really about ehm the the ownership and control over women's bodies. And second ehm of the pressing issues, I think, is also in terms of ehm, again in relation to ehm women's bodies, women's health or sexuality is ehm how the interplay amongst patriarchy, sexism, heteronormativity, and ehm, the role of ehm ehm the capitalist and ehm you know, ehm neoliberal structures (MM and JC nod) as well ehm promoting this.

[0:23:56.7] On one hand, we've seen like the advantage in technology facilitating ehm more opportunities and ehm for instance, benefits ehm for women and girls. But as well ehm their role in terms of shaping this discourse and this conversation about ehm global health ehm should not be, ehm you know ehm, overlooked in terms of ehm in, in terms of the pharmaceutical or the medical, the health ehm ehm corporations. How they get away with ehm really profiting or profiteering ehm from from health per se and ehm instead of also ehm promote. You know, their role could have been aligned with business for human rights and corporate social responsibility is supposed to promote in fact ehm ehm sexual reproductive health, or, you know, attention and respect for for women's health in, in, in, in all aspects. But again, they have been (this silent by standard?), but also influencing the global discourse. And lastly, of course, ehm in terms of the socioeconomic ehm ehm you know, contexts, of course, coming from the Philippines, we are a developing country, and health would not be ehm seen as a top priority of ehm of the government, where resources are allocated. In fact, ehm, especially in, in the tide of the pandemic most of the

pandemic response is, you know, in relation to vaccination or ehm for example, ehm ehm quarantine or isolation and all the other aspects. But in terms of if we talk about feminist ehm approach in terms of ehm of this public health emergency or crisis ehm there's really very seldom ehm attention to ehm how ehm Covid affects, you know differently. In terms of the gender dimension of Covid, and how women are also are not ehm part of that conversation in terms of ehm decision-making and policy-making, and you know, response and prevention. So I think I will end there and over to you, HE.

[0:26:08.5] **HE:** Yes, thank you. So many aspects you mentioned. Ehm but I just want to collect everything, so, as I said, you're the ones who should be talking more than me. So who wants to go next. (8)

[0:26:30.5] **JC**: Ah, oh, please, MM. (MM signals JC to keep on talking) Ok, well, maybe I'll go next because I think what I wanted to say ehm really builds on what CJ said, which I thought was incredibly helpful. When you first asked the question, HE, my immediate thought was that the biggest structural challenge in global health ehm is the role of the unregulated private sector ehm in the delivery of health care ehm and the corresponding myth that the private sector is more competent to deliver healthcare than the state, ehm, which is not to say that the private sector should not play a role in the delivery of healthcare, it can and it should, and it will. Ehm but the lack of and to sort of equity informed, justice-informed social regulation of that sector ehm has led to tremendous health inequalities, and I think that ehm feminist economics (CJ nods), with its emphasis on equity and justice, provides a very important antidote to that. Ehm and to just give a couple of examples the kinds of unregulated industries that I'm referring to here, of course include the pharmaceutical industry, ehm whose patent monopolies not only drive up the price of essential medicines, but prevent the development of the most essential medicines that we need to confront today's public health threats. The insurance industry, which at least in my country the United States has held our healthcare system hostage for decades, and left tens of millions of people a pr/ a profoundly financially insecure ehm, and having to choose between their lives and their livelihoods when it comes to paying for healthcare, but also other industries, the medical equipment industry, the diagnostics industry, I mean these are all examples where, of course, as I said, the private sector could play a role. But where you see that political leaders through a kind of soft corruption have been seduced into the notion ehm that the private sector can

deliver on global public goods more competently than the government can, and structurally I think many people have fallen victim to the kind of myth of the, the bureaucratic state, being ehm slow and incapable of delivering on public goods, and I think that's one of the great threats that we face in global health. I, I also think that that's very linked ehm to the larger threat of authoritarianism. I, I think that political leaders who ehm are not held to democratic checks and balances are much more likely to enter into sweetheart deals with corporations to be seduced by corporate lobbying to be bought, and so the erosion of democracy and the erosion of political checks and balances, and the balance of power between the executive branch, the legislative branch, the judicial branch is so profoundly linked to ehm the kind of corporate capture of healthcare because ultimately it's the decisions of political leaders in countries with weak governance and weak rule of law and rising authoritarianism ehm that permit corporate capture of public goods. So in some ways those two issues of authoritarianism and corporate capture are very linked. Thanks.

[0:30:38.7] **SKY**: Yeah. So I can't agree more with JC. I also feel that the private sector is one of the major threats. And in a country like India the private sector is actually very vast. It's very different as well. So you have an individual doctor who is providing care. You also have someone who's not trained, as in medical, you know, procedures. He's not a trained doctor, but he's there, and they're all there. They're the first point of contact for most marginalised people living in rural areas because you don't have trained specialists, you know, available in those sectors. So obviously they will be turning to these people for ehm you know medication, treatment (JC nods). And that is one kind of danger that ehm people especially marginalised communities face in the sense that they're exposed to providers (JC and SE nod) who are not trained. They don't have the technical know-how. On the other hand, we have the other extreme of having corporate hospitals which charge ehm, you know, exorbitant rates. Ehm, and they indulge in a lot of (analytical?) practices unfortunately, we heard of real life incidences where people have been dead, but they've been continuing to stay in the ICU just to hike up the bill. Ehm, so all of these things have also happened, and the problem is that the, the private sector is completely unregulated in a country like India. Ehm, there have been moves to try and regulate it, so we had something called the Clinical Establishment Act which the, the government, the central government could have mandated and said that all states should take it up. But then very conveniently they said that no, health is a state subject, and

therefore it's up to the states whether we want to take this up or not. And private sector doctors are in arms and up against it. There are very few states who have actually signed onto it, and even after signing onto it, there's been no, you know, rules actually framed to enable that Act to be translated into reality. So it's just a piece of paper currently. I think that's one of the major challenges. And another challenge that I would also like to touch upon is really the entire medical education and the way in which this education is given. So you know the principles of human rights, and all of that are not taught to medical practitioners (JC and SE nod). So they see a patient who's coming in as just a, a person who meets a case. It's not a person, you know. And then, if this person, the doctor, is placed in a rural setup, and you know, India is still very deeply regimented by cast and class inequalities. And we have so much of the lower class coming in. Doctors who are from upper cast tend to look down upon them. Even if they are from the same cast, there is a power equation which is there. You know so they are the more knowledgeable, they are the, in a position of authority, and the way in which they treat people who have come for treatment, especially if they're from marginalised communities, is a problem. So I feel the entire medical education has a lack of empathy. The lack of these skills, which you could call soft skills, are very important, they're critical. But they're not part of the medical education. So you have turned out a large number of doctors who can't be bothered about using (incomp.). And when you have people like that treating you, then it is no surprise that the people lowest in the hierarchy get the worst treatment possible. If you come in with money, with power, with status, you would be guaranteed good treatment. Otherwise, ehm, it's your la/ luck basically whether you survive or not. So I'll just stop there.

[0:34:19.1] **ER**: Maybe I'll jump in because, and the point I had wanted to make really was picking up on the idea of power and, and the way that kind of power inequities are really baked into the global health architecture (laughs), and in part because of its history, but also because of the way that we continue to perpetuate them either as individuals or institutions. And even when we might set out endeavours to try and do otherwise. Ehm and kind of I see this reflected both within global health, so if we look at inequities between, say research partnerships, or the way that implementation of global health programmes might happen in countries we still, even though it's consistently called out, and has been for at least the last (laughs) 50 and 20 years in the sense that we can still

fly in experts from the so-called (uses air quotes) Global North into countries of the so-called the Global South and that there's this one-way direction of knowledge and influence, and and really a, a lack of it, yeah, there's this, there's a paper out, there's a few papers kind of the definition of global health itself is contested, right. Ehm, what do we mean when we say global health? Do we all have different ideas? And there is this potentially reductive idea that global health is public health somewhere else, and, and that you are only doing global health when you take your expertise somewhere else rather than thinking, as you say about all these intersections of class and power, and thus global health applies wherever you are. Which also means that a the knowledge and the power the knowledge can be bi-directional and multi-directional (uses a lot of gesture), and, and I think we still, that's something that we still don't address particularly well in reality, even though we like to talk about it a lot, which I think then is in part, and this may be a a controversial (laughs) idea but I think there's a certain amount of liberal whitewashing and slightly in kind of response to JC's comment about authoritarianism that there's a lot of us on the left who really feel like we're trying to do good, and we say all the right things, and it's easy to reflect back a bit "Oh, yeah, but I've done all the right things." And but are we truly being as reflective (CJ and JC nod) as we need to be when we look at the partnerships that we have, the way that we think about funding, about papers, about who has to say. Are we embodying the principles and the ideals that we like to ehm (unint.) And then kind of, sorry that was a bit of a rant. And then, finally, I think again about power. Ehm I think one of the real challenges is that's issues about power within global health. But the way that global health and the health architecture can be used as a vehicle of soft power, of building power, of the role of the philanthro-capitalism as it comes into the global health system ehm and the potential lack of checks and balances and real accountability structures that exist for those institutions ehm in a way that, an advisor here I work for a UN institution, and it is very imperfect. (ER holds up her hands apologetic) But if you look at WHO, for example, the WHA member state structure is supposed to have some form of accountability, first to member states and then trickle down to taxpayers, and for individual, very large philanthro-capitalists, putting lots of money into global health ehm, ultimately the responsibility and accountability so it's with their board, which may just be one, two, or three people. And I'll leave it at that. (MM, SE and HE

smile)

[0:37:47.9] **SKY:** If I could just add to this one point, I also feel that (unint.) not something. So I kind of feel like, you know, ehm while it's good that you have SDGs before that we had the MDGs and all that. But I still think, but because the indicators are all numbers (ER nods heavily, MM and SE nod), you know, so mean maternal rates to be reduced by this time, infant mortality to be reduced by this number. Governments like ours, I don't know if it's true across, but I feel it is, feel the pressure to show an improvement and show reduced numbers when actually things on the ground aren't really CJing a lot (ER and SE nod). So if you have indicators which are more qualitative in nature, you know, which capture maybe ehm experiences not just numbers as indicators. It might, you know, go/ governments might feel the pressure to actually show that things are CJing and moving rather than, you know, fudging all the records and then creating numbers to show that we are better than Bangladesh, for example. So I feel that kind of pressure also kind of skews a way in which ehh, actually ehm health, you know, programmes are rolled down, and the real progress that is made.

[0:39:01.9] **SE:** Ehm, can I jump in? (laughs) (HE nods). Hi. Ehm I want to emphasise the point that you just mentioned. I totally agree with you. Especially with ehm the political commitment to the notion of ehm leaving no one behind, like a it's, it's ehm ehm ehm a huge kind of of a ehm target in order to reach, but the way the ehm policies and the ehm and programmes are constructed are not putting into consideration people that fall, even if, if there is a focus on some of the marginalised groups, usually they are not putting into consideration the people that fall in between the cracks, people from intersectional groups. (CJ, ER, JC and MM nod) Ehm the way the regulations and the accountability ehm tools to track, if the programs being implemented actually meet the targets or not ehm (...) I agree with all the points that have been that have been mentioned ehm (...) some of the things that I came across that might not be ehm very ehm very top-down policy is the ehm the relationship between how the providers are being paid, or the contractual arrangements with the providers, and the way the service is being delivered. So the, the relationship between the purchasing of the hospital services and the way the health services are delivered in a way that is the patient-centred, and meant to meet the needs of the people, that these programmes are really designed to meet their needs. Ehm, I think, yes, this is it.

[0:41:07.3] **MM:** (...) Can I jump it, SE, you you finished?

[0:41:12.5] **SE:** Yeah, yeah I finished. A cough that interrupted the meeting. (laughs) Sorry.

[0:41:17.6] **MM:** Okay, sorry. Yeah, yeah, ehm, I really agree with what has been said as well, and talking about our power structure like I, I always feel like I work in a a widely ehm like white institution ehm white dominating institution, like at the Institute of Tropical Medicine in Belgium, and I really see, like who has the power to decide (ER nods) what is the, you know, most important health topic to discuss right now, are they all/ they always the same people (CJ nods). So, and we've seen it with Covid, we've seen it with monkeypox, we've seen it like so many diseases that, you know, are not discussed. And ehm much more people are actually dying from them. Ehm, so it's like to me one of the thing I, I, I really want to work on, and I, I really developed as well, is like more participatory approaches where people who are concerned by the topic are already taking the decisions, and not people who are like, you know, far away from from yeah from anything. But it's so, it's still so difficult to to like to make that CJe because people still think they are, you know, ehm legitimate to discuss something that they haven't experienced at all. Ehm, so it's always like ehm, and and for research also the the thing. But what you talked about this already, neoliberal university where we have to put like where the quantity of output is more important than the quality of it (ER nods). Then there is no focus on the impactful CJe, there's no focus on the process and only on the output. So, like, you know, the research process or the development of the guideline. Ehm for instance,I, I remember one guidance that was done on ehm data like guidance in Canada that was done on how to collect and how to interpret data on racialised and indigenous person in in Canada. And they actually the, the time to develop the guidelines was like three three years because they start with a draft, and then they shared with all the persons concerned and they put all the inputs and it takes time, like to really listen (CJ and ER nod), you know, or to really engage or to really bring people at the table and who have other priorities because health, as it has been said as well, is not always the main priorities, you know, getting food, getting a roof over your head, is. So, yeah, there are lots of power ehm imbalances if we say it diplomatically, but like a lot of power yeah asymmetries and and inequities. And it's in all all over global health, like research, global health policies, global health yeah.

[0:44:06.0] **ER:** Hello, sorry. Can I jump back in? I want//

[0:44:08.3] // **HE:** Sure, yes.

[0:44:09.3] **ER:** I want to pick on something (unint.) that MM said. So one of the things I've been really interested in as we look at the translation of evidence to policy, is challenging notions of what we mean when we say evidence. Ehm, knowing that for health policy often the RCT is still held up as the global standard. Ehm, so what we mean by evidence, and then who we consider experts when we talk about ehm evidence that's contributed, and I had sat on a panel for WHO evidence to policy thing last year, and I gave a presentation I had assumed was going to be relatively uncontroversial. Ehm and it turned out it was not. The two takeaways that I had anticipated with the uncontroversial and thinking about evidence used in the Covid response. The first was the need to ehm basically move, ehm to use a mixed methods approach to not rely so heavily on the quantitative approaches and particularly, you know, in the in the case of Covid there was understandably a very heavy reliance on the epidemiological data (MM nods). But ehm, the data around the social, the social and cultural impact, and thinking about what things like and the public health advice around hand-washing and physical distancing might mean in a whole dif/ whole range of different contexts really would have been much more beneficial earlier (CJ nods) if these a these kind of other social determinants of health have been taken into account, and that also we needed to prioritise, or not prioritise, but have a greater inclusion of lived experience. Ehm and I was amazed at the amount of pushback (MM nods) that came from people who are supposed to be experts in that area of, sorry, not supposed to be, who are experts in the area of health policy, who felt that by advocating for a broader range of evidence, types of evidence in a broader range of voices, that this meant that I was suggesting that we were dumbing down the evidence, it would inform policy that we were lowering the bar of quality, which was not the point that we were trying to make, but really to say that therein lies the problem. That our notions of excellence, and how we recognise excellence are so wedded to one particular way, predominantly kind of an Anglo-European approach or a Western approach of what evidence is ehm that we discard vast amounts of really important data when we're thinking about effects in health policy ehm and I think MM's point, any of us who's done anything basically participatory knows that it does take so much longer (MM nods heavily) to work in that way, and, but I firmly believe that we reap the rewards and the quality of the policies that we put forward, how well they're taken up by affected populations (SE nods) and, and I also I

hate to say it, but I think they're more cost-effective too in the long run.

[0:46:54.5] **HE:** Yes, thank you very much. You all mentioned very important aspects and I think we could talk about the structural challenges the whole discussion. But I want you to focus now a bit more ehm on the solutions that a feminist global health policy could provide for these challenges, so you can of course still refer to all the challenges that there are. But I want you to consider more a solution-based orientation. What alternatives does a feminist global health policy provide, what solutions are there. Ehm, so I keep this question very broad for the moment, and I can still narrow it down a bit. So yeah, whoever wants to start again just as we did before. (6)

[0:47:43.3] **CJ:** Well, okay, Oh, I, I it, I start again just to ehm start the conversation. Ehm, I would think that ehm amongst the the solutions, would heavily rest on looking at ehm who are responsible in terms of ehm who are the institutions that are responsible, and of course, ehm we know that a from a rights-based perspective, we know that the states are the duty bearers. Ehm and they are, in fact, in the possession ehm to ehm regulate or to formulate policies. Ehm even including in relation to ehm health responses and ehm and the role of the private sector or the corporations. So one would be to highlight in terms of ehm, you know, that understanding by the state in terms of it's our role ehm to fulfil, you know, health as a human right of individuals and the peoples. And ehm from there it's really ehm I was thinking, if it's this is in any way relevant, but ehm, discourse is, of course, in terms of the body of knowledge that we are producing in in terms of health. Like a I, I'm not sure if ehm a again this is relevant, but I remember Michel Focault, whose biopower were how bodies are in fact perceived (JC nods). And ehm, we have to think about ehm health being a right, and you know part of our human dignity and well-being, but ehm again we look back at how ehm discourses have shaped ehm people's perception, and ehm, this, the role of the various institutions, perception, and health is mainly, for for example, you know, in a neoliberal-capitalistic, you know, industrial ehm development frameworks that health is more seen as for labour to produce ehm labour and surplus and to the profit. And I think it's one of the fundamental issues, I think, and it's one of the fundamental gaps in terms of ehm producing more ehm knowledge, and ehm and ehm discourses around how health and how people's bodies ehm, should be ehm perceived, and should be treated. And ehm again, if not from the gender-based violence and a feminist perspective as well in terms of, you know, not only commodification of, of bodies of

people's bodies, but also in terms of ehm essentialisation, like women's bodies are only meant for this or men's bodies are only meant for this, in terms of that they carter me between reproductive or productive ehm, you know, ehm ehm roles, but also in terms of cis-heteronormativity, which ehm have been also very suppressive and repressive (ER and MM nod) for many ehm people of ehm diverse identities. So again, ehm, if if ehm there's also, you know, an interrelation between ehm, the perpetration of of violence, structural or gender-based or otherwise, because ehm this regulation, this ehm how bodies are governed, how health, people's health is being governed. It's also between, you know, other suppression and repression for women's bodies, ehm in/in/ including their sexual reproductive health, their sexuality. Ehm, but also in terms of sexualisation of women's bodies, and the other end, and ehm men's ownership, and ehm entitlement to to women's bodies, and how this also puts the non-heteronormative groups, ehm, you know, in a further detrimental position (JC nods). Ehm, because again, ehm those who are in power in shaping this discursive power of knowledge, of beliefs, of setting the norms in health are really, you know, unchallenged ehm, so to speak. So, and then, lastly, I would also talk about ehm in terms of ehm no/ culture or traditions, and I think indeed, there's been ehm this has been the the great debate (uses air quotes), if I may, in terms of how do we also value and recognise ehm, you know indigenous ehm health ehm (...) practices. Ehm, where you know, of course, the the the entry of this modern technologies, modern sciences ehm trampling on not only the, you know, of the rights of of the indigenous peoples, but also ehm you know dominated ehm the health discourse among other things. So ehm I'm not sure if those were quite coherent (JC and MM nod). But ehm, I will end (JC raises his hand) I will end there.

[0:52:38.8] **HE:** (...) Yes, JC, you can jump right in.

[0:52:43.6] **JC:** So (smiles, coughs) ehm I am, I could not agree more that feminism, takes us ehm from a narrow focus on biomedical solutionism to a broader focus on wellness and health as a complete state of psychological, physical, mental, spiritual well-being (CJ nods), and an emphasis on the social determinants of health. Ehm and I think even the very use of the term solutions in your question (ER nods), HE, in some ways takes us towards (coughs) what I think is another structural challenge in global health, which is the the emphasis on solutions (ER nods) when, in fact, the kinds of things that the devil global health that we're talking about ehm like inequities of power ehm, they don't really lend

themselves to easy solutions and attempts to solve them, can sometimes make matters even worse. Ehm and I think we've we've seen so many examples through the years of how an overly biomedical framing of complex social problems ehm narrows our lens of focus and limits our imagination about what is possible. We see it in Covid-19 right? If you frame Covid-19 as an epidemic, as strictly a biomedical phenomenon, you get the kinds of interventions that ER was talking about. You get physical distancing, you get hand washing, you get vaccines. That's nothing against those behavioural and pharmaceutical interventions. But if you widen the lens and look at Covid-19 as a result of hazardous work, as a result of crowded and substandard housing, as some, as a, as a complex phenomenon whose burdens (coughs) are distributed unequally in in society. Then you get a different (coughs) set of interventions (CJ and ER nod), a much more structural set of interventions. I think the field of mental health is another perfect example of where biomedical solution is ehm the notion that ehm lived experiences and mental health (MM and SKY nod) problems are exclusively diseases of the brain that can be treated with Pharmaceuticals, has also very much limited on our understanding of of the social and economic determinants of mental health. (coughs) That's not to say, ehm that psychopharmacology has not made a profound difference in the lives of many people, but an over-emphasis on biomedical solutionism can lead to ehm overprescription, it can lead to forced treatment, (coughs) it can lead to institutionalisation, violations of bodily autonomy, as CJ was talking about, ehm. So there really are so many examples of this, and I/ in some ways, I think ehm what what feminism, I mean provides in response to this is, it's not only, in my opinion, a different ehm paradigm of thinking about it that is less biologically deterministic. But, you know, if you look, for example, at the Convention on the Elimination of Discrimination Against/ of All Forms of Discrimination Against Women, it it almost gives you a blueprint (CJ nods), right, for the diverse determinants of Women's health, which are, I would argue, determinants of everyone's health. Ehm so the Convention speaks to everything, from the right to representation in political life, to the right to legal status and equality, in private life and in marriage, to rights to housing and employment and education, and all aspects of social and economic life. We know that all of these things are profound determinants of health, ehm that the more women and girls enjoy this full range of human rights enshrined in international law, the better their health is and in turn the better everyone's health is (ER nods). And

that, I don't know, I'm curious what other people think of this if they even want to speak to it, but that to me feels like the beginning of of at least a more concrete way of understanding, a more expansive approach ehm to health that feminism and women's rights brings us in contrast to an approach on that focuses on biopower and the control of people's bodies which we know historically has just flown right in the face of women's bodily and reproductive autonomy. (CJ and ER nod)

[0:57:52.7] **SKY**: JC, you know, I think the one of the reasons why they don't adopt feminist approaches is because it would then upset the apple cart (JC nods and laughs, CJ, ER and MM nod). And it's really good to continue with the existing status quo. Now you use an approach that was JC was talking about, we're talking about, you know, larger issues affecting health. It means that you have to take action to, you know, to address those. And to address that would mean, then, that you have to have a more equitable distribution of resources (ER and MM nod) and really question fundamental things which are easier left as they are (ER and JC nod) because then you're able to continue exercising the power that you have been. So I feel there is a very ehm, there is a very real reason <u>why</u> feminism isn't accepted and thinking around it really isn't promotive because you don't want those kind of solutions. It's going to CJe things radically and you would rather not have a revolution that challenges the existing way of thinking. So, that's what I feel. (SE nods)

[0:58:59.9] **HE**: Yes, thank you. And ehm I mean you also mentioned ehm who holds power, and also the power regimes of capitalism, neoliberalism, heteronormativity, but also coloniality. So we also really mentioned this intersectionality. And how can these power hierarchies be dismantled, or how can the power be transferred? What, what would you think would be necessary to do so?

[0:59:27.0] **SKY**: When we just come and hear from what from the experiences that we had when we were at the ICU. Only way to do it, is really to make people realise that they have rights. A lot of marginalised people don't even realise that (CJ and SE nod). So it's it's not an entitlement that the government is giving you. It's your right, as a citizen (JC nods). And unless, you know, that consciousness is raised ehm in people, they will not demand for their rights. And it becomes very convenient, then, to see the government as a benefactor, and then we, you know, (we're waiting for dull?) and ehm it means it's just much easier, but if you <u>build</u> that consciousness of this is your right, and you are

entitled to it, and you must demand for accountability, the government is accountable. They have to give it to you, you know. Then, the challenging, the status quo and, you know, shifting the power, all of that begins. It's only possible when you get people together and get them to ask those kind of questions (MM nods). I'll just stop with that.

[1:00:31.7] **ER:** Yeah, I think this term shifting power is a really interesting one (JC smiles). Ehm, and I say that we've had a whole series called Shifting Power. (laughs) So we've been talking about a lot, and really have not come to an agreement internally with people that we engage with, whether we think for power is this finite resource. Ehm and so, what does it actually mean to shift power? (JC nods) So we've been working with some expert partners in ehm South Africa, been working with this fantastic reverend who runs a foundation, ehm and I I describe his work essentially as Trojan horsing feminism. And his argument is that the patriarchy doesn't serve anybody ehm and that men in his in his context in South Africa are told, men must, men must, men must provide, men must be strong, men must be this. Ehm and that the the patriarchy is continuing to oppress those men, and that feminism is one of the roots that he's using to kind of help liberate. (CJ nods) And when they get pushback, he says "Look this isn't a zero-sum game (JC nods) by more by women having more power that doesn't mean that you're going to lose less power. And this idea that kind of power can grow. But then we have other partners in this same process, this is all part of the Lancet Commission on Gender and Health, ehm our Kenyan partners, who are at the opposite end of this scale. And they will tell you that there is only so much power to go around. (JC and SKY smile) And that if there is more power for women that means that somebody has to lose. And that actually losing the power is really important, and that there's a combination of forcibly taking that power, and pe/ and other people who are in position to power, being willing to, not being willing s/ maybe either willingly or being pushed to step back and to conceive power. Ehm and there isn't really like a definitive answer to this other than just, I think it's really contentious. And so the idea of how we have a solution. Ehm I, you know, as JC said, I think it's really hard, because this idea that there's you can just have a bit more power to go around, and that empowering others empowers you, is the nice answer. It's the less threatening answer. But I don't know if it's true. (laughs) (...)

[1:02:48.1] **JC**: Ehm (...) so if I/ I've / that was absolutely fascinating (ER laughs). I'm curious (coughs), I know I'm not supposed to be asking the questions, but I'm curious, I'm asking

myself (...) whether feminism as a theory, as an ideology (ER nods), takes us to the mutualising view of power that your South African colleagues articulated, where there is an abundant power to go around that the more power you have, perhaps the more power I have. (ER nods heavily) Actually, we neutralise each other's power. Or to the zero-sum view of power that your Kenyan colleagues articulated, which is, you know, the more power I have, the less power you have. My gut tells me that feminism is consistent with a mutualising view of power (ER nods), and that the latter view almost treats power like a commodity to be distributed. So that whole idea, like the commodification of power (CJ, ER and SE nod) strikes me as a threat to any kind of vision of health for all. Ehm and I think we need to overcome the myth of scarcity in everything. (ER and SE nod) Ehm whether that's power itself, or whether it's medicines or medical technologies, or health services or whatnot. Ehm so that's sort of what your intervention triggered for me.

[1:04:36.8] **MM:** That's a very interesting discussion. Yeah, indeed, for me as well, like I I'm I'm tend to think more that, you know, if ehm yeah, people will have to lose some power (JC nods heavily and smiles, ER nods) like to actually share resources, share access to resources, financial like anything and I'm also going back to my yeah years in anti-oppressive community groups, and they were like two people were saying "ok there are two ways to to make CJe, either doing the revolution or reform" (ER and JC nod and smile). So and and in on like anti-oppressive groups, do you, yeah, do you s/ I see of all frozen yeah. And people were saying "Actually yeah, it's either, it's it's both actually" (ER signals agreement, JC nods). Like, I don't think there's one solution to to everything. I I think there's more like context-based solutions or you know solutions apply to different settings at different moment with different people involved. Ah, and I totally agree with that idea, like, you know, how it's ehm it's very fashionable to to, you know, to think about solutions only when we haven't dealt with the issue at stake at first (CJ and ER nod), like we yeah, we have to deal with inequities at first, and and then we can ehm build on solutions for sure. Ehm because our solutions they're like people who are, you know, improving their own lives without global ehm health actors involved. Ehm, so yeah. But this is definitely a very interesting discussion. (...)

[1:06:17.0] **HE:** Yes and I would also ehm like to focus more on the actors. (SE nods) We also mentioned it a bit, and also ehm CJ mentioned accountability ehm and she mentioned the state and SKY mentioned the the individual human right that everyone has to ehm

recognise for themselves. So yeah, who is/ who needs to be involved in a feminist global health policy, or who would also be responsible? And maybe regarding more the global level and is there even an answer to that? So ehm yeah. What are your thoughts? (...) Okay and MM's connection is not so good, so she just turns her video off for a second. (7)

[1:07:13.0] **CJ**: Ehm, okay, so like I mentioned ehm in terms of the actors ehm we know that ehm when we talk about the state it's, it's also not homogeneous. It's / we need to ehm, you know, we are aware how nuanced ehm when we approach, ehm how we view, and how/ what we expect from the state. Ehm because, of course it ehm we're talking about feminist global health policy, but ehm really in terms of ehm the overall governance of ehm of ehm of nations of of the world, in fact, are in the hands of a few people. And this ehm in in ehm this would show us ehm in terms of patriarchy and ehm sexism how really power are/ power is in the hands of a few men, and we see also how ehm the political economy of of power as well, those who are ehm in power would have, you know, more wealth and resources, and ehm they continue the status quo because they benefit from it. And so, when we talk about ehm ehm (...) the the hierarchy or the inequities ehm. It's also about how can we dismantle this ehm this ehm this structure, this dominant structure that perpetuates inequality, and perpetuates ehm very oppressive, discriminating, ehm and repressive health policies. And ehm so, in fact, ehm we can define the different actors ehm which of course that would include ehm the financing ehm, you know, sector, ehm of course World Bank, ehm, you know, ehm this ehm m/money lending ehm institutions, these financial institutions who also have ehm that hold over ehm governments. Ehm and in terms of how they also have preconditions in terms of ehm lending or ehm, you know, loaning ehm funding for for the governments to operate. Ehm, but again we see that ehm how the resistance from ehm well, from academics or from ehm (unint.) ehm the progressive groups, the feminist groups, the movements, the social movements. So these are key as well in terms of really pushing for ehm, structural CJes, or, you know, transformation. Ehm, but also in terms of the the role of ehm human rights institutions, I mean multilateral, such as the United Nations, or those ehm Wor/, you know, norm setting ehm institutions. Ehm and even, for instance, those who are big ehm, you know, ehm funding of ODA, official development assistance, as well. And ehm also in terms of ehm the academia, and you know, in terms of the schol/ the the scholastic production of knowledge. And ehm we also need ehm to have more, I think ehm, I'm not sure if it's quite simplistic to ehm involve ehm the education sector for this because even the education

sector is privatised, it's also, you know ehm there's a lot of problems as well. But ehm in terms of really ehm, you know, transforming or shifting the consciousness and ehm the norms in a society we also have to look to the quality of education that we're having ehm in the society.

[1:11:06.0] **SKY:** If I may just add, for me it is really community-based organisations (CJ and MM nod). Which have or follow feminist ideology and principles (JC nods). They, I feel, are key. Because they are the ones who are in the community. They have the trust with the community and they are the people who've been really, you know, work on consciousness, raising awareness, raising ehm/ So for me, those, they are very key players. Civil society at large yes, but specifically community-based organisations, which have a presence, and ehm subscribe to feminist ideology. (7)

[1:11:48.4] **MM:** I don't know for you, SKY, but like for, like in Belgium, for instance, they just started like these community health care workers, and most of the time they are unpaid or not paid at all. So you know, having to ask them as well, you know, they do a lot of, I I totally agree with you, they they do an amazing job. Ehm but then funding is missing, you know, for them to do a proper job ehm sometime. Like to do, yeah, what they want to do. I don't know in your context how it is.

[1:12:19.2] **SKY:** No. So I'm I'm kind of distinguishing between, so we also have what is called the Asha (MM nods), who are community-based ehm / they are people from the communit/ from the community who are part of the health system. They're seen as volunteers, they're asked to do certain services and the government compensates them for each of the services. I'm not talking about that. I'm talking about NGOs, but NGOs which are nested in the community (MM nods). So they really act at village level, ehm at the block level, you know. And they're right there in the community. So those kind of organisations, I'm talking more about them, not about ehm (...) community health workers, you know. So I would make that distinction between the two (MM signals understanding).

[1:13:07.2] **JC:** Ehm, I I th/ I think we alluded to this before, but I think in terms of actors who need to be involved in a feminist global health I would also add ehm feminist economists (ER nods), who are, of course, not (...) monolithic, but ehm (...) you know, tend to bring both an analysis of (...) equity and justice that's rooted in feminism as well as an appreciation, and ehm MM you just alluded to this, ehm of and a regard for women's lives, and women's work, and very often unpaid work, in feminist analysis, and an

appreciation of how ehm (...) health economics, as it's currently constructed, ehm can disproportionately burden women, right. So who assumes the burden of unpaid care, who assumes the burden of catastrophic health expenses ehm in places that don't have universal access to health care. Ehm very, you know, who's ehm (4) whose health in a family is the greatest determinant of everyone else's health. (laughs) I think it's, you know, time and again the answer to these questions is women, ehm I think more and more we are actually starting to see feminist economists ehm influence the political economy of health. Ehm so, for example, you have, you know, within civil society organizations like Feminists for a People's Vaccine which have taken their understanding of ehm power differentials to the debate over a Covid-19 vaccine and push back on patent monopolies, or ehm you have the, you know, at the multilateral level the World Health Organization's Council on the Economics of Health for all which is made up entirely of women. Ehm I don't know if they all subscribe to feminist economics per se. Ehm I think that, yeah, I think I I could not agree more with what SKY said about community-based organisations that subscribe to feminist ideologies so not at the exclusion, but I, the ehm role of feminist economists, gives me some hope as well.

[1:16:04.1] **SKY:** Another thing that I would like to also add are ehm networks and coalitions. Like, for example, the People's Health Movement. (CJ, ER and JC nods) So in India we have a very active ehm Indian chapter, which is called the Jan Swasthya Abhiyan, and they've been very, very active in, you know, ehm raising pertinent issues around (SE nods) government (moves?) privatised to many other things which actually subverts the interest of marginalised communities and ehm universal, you know, health, access to health, not just health coverage. So I think those also are very critical players.

[1:16:45.2] **HE**: Thank you. Now SE, yeah, I see you are trying to jump in.

[1:16:50.7] **SE:** Ehm yeah, no, I was going to ehm follow back on what SKY said. I actually love the ehm notion of ehm empowering the community-based organisations, but the problem happens that in some paternalistic leadership ehm (...) kind of of ehm contexts, sometimes these ehm organisations don't have the ehm the power (MM nods) to implement or to do what they are really trying and reaching out to communities and ehm finding ehm real world solutions because of and ehm in some context they actually turn to be ehm kind of hollow voices of how the political direction is going. So ehm in as much as the the civil societies and community-based organisation can play a tremendous work in in implementing

ehm feminist policies. I think the key to ehm their role goes back to how the leadership of the country, or the context of the, the political context of the of the whole ehm country is ehm based. Yeah, this is what I wanted to do ehm/

[1:18:19.7] **SKY:** Yeah SE, I can't agree more (SE nods). Ehm most of you, I mean people who follow India would know now that a lot of activists are being jailed, it is not easy anymore and it's actually a threat to your own safety and well-being. Ehm the moment the regime becomes more and more, fundamentalism increases, and you know there's more of ehm ehm in India this course is really CJing a lot. So it is dangerous, yes. But I think it's still the only hope.

[1:18:51.4] **ER**: I ehm to count to that idea of the only hope, but also to take this idea of a two-pronged attack. I mean I think, linked to what JC said about feminist economists, I think more policymakers, more people sat in funders that have positions of power within global health that subscribe to and live feminist values is really important. Ehm and I think that's, (sighs) you know, it's it's people in power now, but also/ So one of our Kenyan partners, Young Women Leaders Institute, when we ask them about success, they're really playing the long game, you know, they've been around, they've been active in Kenya for the last twenty years, they have seen young activists come through their networks, who are now starting to infiltrate positions of power, and to be able to influence from the inside the same way that they're agitating on the outside. And so I think that also gives me a sense of hope. It's not an immediate sense of hope (CJ and SE nod), but you know, but longer term ehm if we, if we really can have more people living and embodying the ideals that then this bottom-up and top-down, we really might start to see CJe.

(10)

[1:20:13.9] **HE:** Thank you. Anyone wants to add on that. (MM raises her hand)

[1:20:16.6] **MM**: Yeah, I I will jump in quickly because we'll be talking about feminist approaches, yeah, or approach. But I I think they are plural (ER nods heavily), and like for Europe and and North America it's very white dominated, you know, by yeah middle-aged educated white women. So ehm if we think about community, but ehm I'm sure ehm all of you, if you think about community-based organisation, it's, you know, other kind of, like not other, but like different, no not different, what what word, various types of feminisms (ER nods heavily) like let's say Black feminism, Muslim feminism, like all of this. And and I Think, if you think, HE, maybe that that's for you, if you're doing research ehm and

you take you want to take you want to take a feminist approach, and I'm a whit/ white researcher like you, so using inter/ intersectional lens, you really have to be ehm careful not to erase race from your analyses (ER nods) because, ehm intersectionality comes from you know, ehm racism and sexism at the same time, like the analysis both of the two of them, and class, of course. But ehm intersectionality has been whitewashed (CJ and ER nod) in the last years and decades by white feminists. So it's really ehm, yeah, just a small ehm thing I wanted to share with you because that's very important to me. Anyway, yeah, thank you. (...)

[1:21:43.2] **HE**: Yes, thank you very much for stressing this and I also wanted to ehm ask you to ehm consider these aspects in the discussion because now we're focusing also a lot on on women and women's health. But the feminist approach also includes ehm decoloniality (CJ, ER, MM and SE nod) and considering racism, classism, so ehm, if we can maybe elaborate a bit more on these aspects, ehm maybe also what ER mentioned in the beginning, I mean global health, ins/itself is deeply colonial, the history, but we also see it today, ehm maybe what would be alternatives to that, or how can we approach these? Ehm, yes, so maybe someone wants to add on these aspects, or also new aspects that are linked to this, that it's a truly truly intersectional.

[1:22:31.3] **ER**: So I'll jump in there. And so, first of all, to echo MM, I think the idea that it really is about a pluriversality of approaches (MM nods) ehm and that is because we have a plural/ we have many, many contexts in which this is being applied, and it's the same with the conversations around decolonising global health, there is no single way to decolonise ehm or really to achieve the decoloniality, I think, more than physical ehm decolonisation. Ehm and that's because of the contextualised nature of all that it is that we're doing. Ehm to pick up on your first point. Yes, we have focused, there's been lots of talk about ehm women, and I am glad that you brought that up, HE. I think what's really important about feminist methodologies or approaches is that it's, for me at least, in many ways, a set of values and principles that is not exclusively about women. (CJ and MM nod) It's centred on the idea of equity, ehm and that may mean, you know, in order for us to reach some sense of equality. And I'm always a little bit cautious about the idea that everybody will always be equal ehm because I'm not sure it's realistic in the world that we live in. That we might put the/ that we're going to privil/ we're going to privilege certain groups. Ehm, but it's not, feminism does not equal women (MM and

SE nod), I think, is what I'm trying to say, and it really is about the the breadth of the human experience and and people's lived experience. Ehm on the decolonising, the coloniality question that you asked. I mean what can we do about it? The reality is that it's our history. Ehm (laughs) we can't, we're not going to be able to go back and rewrite that (MM and SE signal agreement) as much as people some people might like to try. Ehm so I think it's really important that we acknowledge where this beast, that we call global health, comes from ehm and what that means about ways of acting that are baked into the (...) the way that we work and the systems that are perpetuated. Ehm I think you know, in terms of how we address it, I want to avoid the solut/ deterministic solution approach of Jonat/, you know JC has rightly critiqued. I think it's about thinking about multiple levels and sites of action. So the role that we play as individuals ehm that reflexivity about how we're contributing, that role that organisations play, the institutions that we work in, and how they engage with partner institutions, and how we may or ma/ how we might be able to influence that. Ehm but then also really to to where you have enough privilege and safety to be able to do so. Ehm, to be able to call out the bigger structural issues, when we c/ when we can, ehm and I think it's really important to note the the privilege that comes with being able to speak out. Ehm as SKY mentioned, it's becoming increasingly challenging in a number of contexts. And when we talk about decoloniality and challenging the status quo with other colleagues within ehm the global health system, many of them say it's something that they feel very passionately about, but the/ they don't feel comfortable talking about publicly (MM nods) because their position as non-white, ehm not male, not based in a Northern institution, means that their jobs can be on the line. (SE nods) Their funding is on the line ehm and it makes it very difficult to be as vocal as they would like to be.

[1:25:49.9] **HE:** Thank you. Anyone else who wants to add on that, the more broader frame of feminism, so that we don't focus too much on women, yeah, CJ.

[1:26:05.7] **CJ**: Yes, well, ehm, I think I'm going to pick up on the point of ehm decoloniality or decolonising ehm, you know, the discourse on health (ER nods) ehm as it is. Ehm, but we also have to take into the account ehm the point of access, like access to education (ER nods heavily), access to ehm, you know, who is being published (ER and MM nod heavily), who is ehm, you know, who holds the dis/ the discourse, who holds the knowledge, and of course ehm it is indeed, ehm a, a long stretch to to expect ehm from the

communities too. Also, we want the community to be there. We want ehm diverse, indigenous, and, you know, from various cultures ehm good practices, good health practices, and ehm and discourses up there, you know, have a seat on the table, have an ehm equal opportunity in the in the conversation. But ehm again, it's a long, long way to go because ehm we still need to talk about ehm, you know access to these platforms. Ehm and the the the resources that it requires ehm for this ehm varied diverse ehm knowledge, ehm sources of knowledge to be ehm also in in in the position that holds influence. Ehm but I agree, of course, when you talk about ehm health, a feminist global health ehm policy is not simply about women's bodies. But we ehm identify the sites ehm as feminists. Ehm, you know. Ehm, you know, taking off from the feminist perspective, we identify the the sites of oppression and ehm inequity and inequalities. And so and ehm, the prevailing dominant ehm, you know, ehm group ehm shaping the discourse, influencing the discourse, controlling the discourse, that, in fact translates to the the current status quo and the statu/ the current ehm, you know, conversations around around health, and that has concrete ehm impacts on people's ehm health or people's lives. Especially those ehm again ehm those in the poor or lower income countries. Ehm and so again, if we talk about ehm the alternative, how do we also bring about that shift ehm to, you know, equalising ehm the conversation where those who are underrepresented or misrepresented are able to also have a voice, and you know, a seat in the table. (ER, JC, MM, and SE nod)

[1:28:57.0] **JC**: Yeah, I think it's ehm (...). For me it (...) it involves a larger shift in mindset ehm from focusing (...) ehm not only on what we do, but how we do it (ER nods). Ehm and I think that the how (...) often gets dismissed in subtly or not so subtly sexist terms as (...) kind of process ehm as opposed to substance. Substance is what people of action and intelligence (laughs) focus on. And I I think that there is a sexist implication in that, and ehm I mean feminist leaders have certainly taught me that how we do things ehm is not only as important as what we do, but it determines (ER nods) what we do. And critical to that is ehm really the principle of meaningful engagement (CJ and ER nod) and meaningful participation, of all people, particularly ehm people historically on the margins. Ehm and so (...) a feminist global health, I think, commits itself every day to enabling the participation of diverse, historically excluded voices and actors. Ehm (...) you know again, not only as a kind of ethical imperative in and of itself, but as something that gets you far, far superior ehm (...) ehm results (CJ, ER and MM

nod), if you will, yeah.

[1:31:07.9] **HE**: (...) Thank you all. Ehm, so now I'm also thinking about (...), is there something that we can already build on? I mean, we mentioned all the structural challenges we have in the beginning, especially also private sector, neoliberalism, colonialism. But to have, like more of an optimistic output if it's possible, is there something, maybe a feminist global health policy can build on, or some, I don't know, some examples or some structures that are already there. (8)

[1:31:52.8] **CJ**: I think we can build on ehm the current ehm, you know, ehm well not necessarily we're putting all our eggs in in one basket, but ehm we can build on how ehm we have this ehm internationally, I mean widely accepted ehm norms, human rights norms and standards and convert/ and conventions. Ehm, where that holds ehm, you know, ehm the state to account, including ehm the private sector to account, or you know, certain ehm institutions to account. So I think ehm, for example, ehm, you know, the CEDAW (SKY nods), or ehm even for ehm health specifically specifically ehm the other ehm conventions on ehm from the World Health Organization. Ehm, this can be something that we can build on and I think ehm more, you know, see it m/ (...) proliferate, or you know, reach the consciousness of people in terms of how we should ehm look at health for people's health and ehm the public ehm, you know, health response or public health policy. So ehm, we have, we we need to also see the value in ehm this policy frameworks ehm in adherence or pursuing to the conventions or the human rights standards. And ehm, of course, these policy frameworks have been long and hard fought for ehm, especially the community-based organisations, like the movements ehm in keeping, you know, feminist social movements. And ehm and to also recognise that ehm, we have ehm, as SKY I mentioned, you know, community-based groups, but also to have ehm a a nuanced view in terms of within the community-based organisations, I mean, who are those who are holding power, and you know ehm making decisions as well because we all know that ehm, you know, community-based organisations sometimes are, you know, reproduce certain patriarchal or or ehm conservative ehm practices (SKY nods), or or or or or beliefs. So again, ehm what we have that we can build on is ehm these multilateral institutions. Ehm ehm that state and you know, private sector listen to. And ehm the policy frameworks at least at the country level ehm and ehm and positioning also our states, our governments to ehm adopt progressive ehm positions in in public policy ehm discourse ehm in in in important ehm platforms and and dialogues. And we have to thank the,

you know, human rights groups, community-based organisations, feminist groups and the movements for that. And we do have this ehm movements, our strong movements, who really ehm ehm have an important role to play, including in terms of bringing even the concept of intersectionality and ehm, you know, challenging ableism or heteronormativity (MM nods) and ehm and the other ehm, you know, ehm sites of ehm oppression and repression. So (...)

[1:35:14.9] **SKY**: So I (unint.) agree with CJ and I think that ehm all of these international processes like (UPR?) and, you know, ehm the Beijing (incomp.) and all of these (CJ nods) reviews that you have are good because they also give space to civil society organisations to present. It's normally before you have the nation states coming in and giving the reports which could be skewed, there is a par/ there is ehm and ehm in / something that happens before where you have civil society coming in and then presenting, you know, the maybe the situation of human rights, or how much has CEDAW being met, or what has been the progress after the Beijing Platform. I think those are good because they help to keep the pressure on, and while governments might not, you know, they cannot be entirely dismissive of it because there is also a reputation to consider. So I think that does help to put pressure, and it is a good starting point to build up upon others. Everything is very complex, but we can't given to that. So I feel as civil society organisations or spaces which have been created for us, we should really be participating in it, and bringing the voices of the most marginalised to these places (MM nods). So this is a (...) that's it from me.

[1:36:39.9] **ER**: (8) I think there's ehm some good examples of funders out there who are starting to embody feminist principles and the work that they do, that might also be examples to look to HE. So the ones that kind of come to my mind are the Ford Foundation and also Global Fund for Women ehm they both very clearly have a/ they do focus quite heavily on women's health. So I think that is potentially a limitation. Ehm but they explicitly have feminist principles and values underpinning the work that they do in the type of projects they're looking to fund, ehm and in the way they expect grantees to engage and participate with ehm members of affected communities and and other relevant stakeholders. So I think those would be two good examples worth looking at, particularly given that money does equal power.

[1:37:36.0] (SE writes in the chatbox that she has to leave. Everyone else waives to say goodbye and smiles.)

[1:37:43.9] **HE:** Thank you. And bye, SE, thank you for joining. (smiles)

[1:37:49.4] **JC**: Yeah, I am (coughs) I know, HE, you were asking for sources of hope and I I don't know if what I'm about to say sounds hopeful (ER smiles) or not, but I I think that ehm (...) wherever people (coughs) wherever people have rejected, (coughs) excuse me, rejected authoritarianism ehm is a source of hope, right. And why I say that is that (...), and this is the pessimistic part, we've we've seen it over and over again, the way in which ehm authoritarianism, autocracy (CJ nods), ehm thrives on ehm appeals to traditional family values (uses air quotes) that are profoundly (CJ and ER nod) anti-feminist, ehm thrives on an anti-gender ideology and the kind of creation of panic, a moral panic around the the end of gender which has led to terrible violence and discrimination against transgender people (MM nods), ehm thrives on kind on the the politics of the big man. Ehm, and what ehm I think the feminist philosopher Bonnie Mann coined the phrase sovereign masculinity, right, and the the ehm it thrives on ehm I think some people's, I don't know, kind of subconscious desire for strong decisive authority figures who demand and command obedience and conformity, and and and somehow, perhaps, that makes people feel safe in certain circumstances. Certainly, authoritarianism thrives on anti-abortion ideologies, attacks on women's reproductive autonomy (CJ nods). And you see this, I mean, you see this in the United States, you see it India, you see it in the Russian Federation, you see it in Brazil. I mean I don't have to name (laughs) which authoritarian leaders I'm talking about (ER smiles) so and ehm. So any time social movements, voters, ordinary people reject that ehm even if it's, you know, a referendum in the State of Kansas that fails to ehm outlaw abortion in its constitution. That to me is where the hope is. (MM nods)

[1:40:50.1] **HE:** (6) Yes, thank you also for mentioning the the opposition to feminist movements, and because I also have the feeling, these anti-gender, anti-feminist groups they are very good at ehm gathering people behind them probably also because they are based on fear, as you just said JC. Ehm but it's much more harder for feminist movements, I think to to address the people or to to build this movement ehm as it is for the ones who are just against it. So what would be maybe helpful for a feminist global health movement to emerge? Ehm what what would be needed.

[1:41:40.3] **ER:** So I think there actually is a very vibrant feminist global health movement. Ehm and kind of a lot of activists that are working in this space. And when I use the term activist, I think it's kind of important that we remember that the term activists can

apply to people across a whole range of backgrounds, situated in a whole load of different organisations. And, you know, we've been talking a lot about the rise of anti-feminists movements, and I I think sometimes we're a bit idealistic or naive and like, oh yeah, we've got all these amazing new tools and social media allows us to organise, but we have to remember that even if we don't agree with them ideologically, there are also lots of very clever very articulate, very well-organised people in the opposition (CJ nods) who are weaponizing many of the same tools that we are using to our advantages. Ehm and you know, I think Roe v. Wade is, in JC's context, a perfect example of a long term, very sustained, very well organised, oppositional movement that has been able to affect CJe, and we underestimate that at our peril. Ehm but I don't think I don't thin/, you know, if if anything, what we've seen is the many protests all over the world, not just in the US over the last few months have shown us that there is a still a very vibrant feminist movement when it comes to health. (...)

[1:43:09.9] **JC**: Yeah, I mean, I hope I don't know. I hope it's ok for me to also suggest that (...) in order fo/, and this is true of any movement, in order for a feminist global health to emerge ehm I suspect that feminism, like every movement or theory or ideology, needs to wrestle with some of its own demons (ER nods heavily, CJ nods), right. Ehm particularly the, and this is not a new argument, but the perception, the concern, the narrative, that feminism is the province of kind of white middle class, cis-women. And you see that expressed in trans-exclusive feminism (MM nods), in sex worker-exclusive feminism, ehm and in BIPoC, Black, Indigenous, People of Colour, -exclusive feminism (ER nods). So, and I say that because, you know, HE, you started out very clearly, identifying, I think, rightly, with a kind of intersectional feminist justice lens. But, you know, ehm the human rights movement as a whole is having to grapple with many of the same questions (CJ, ER and MM nod) as having to work to decolonise itself and ehm recover from many of the embedded hierarchies of power that have bedevilled that movement for a very, very long time, and I think that those kinds of healthy conflicts will only make the movement stronger, although I should say that sometimes they're like not healthy at all. Ehm and (...) I, I have heard ehm and and I I can't speak for them, but I have certainly heard ehm people of trans experience, sex workers, ehm (...) referred to, and express actually quite considerable fear and insecurity about what they refer to as a kind of pseudo-feminism that erases their existence. Ehm that is, I don't know, that (sighs)

(4) dictates who they are and (MM nods) what they do with their bodies as much as (...) the men, who traditional feminists condemned, do and have historically. Ehm so I think some amount of wrestling with those fissures within the movement is going to be really important.

[1:46:17.7] **ER**: I, I think building on what JC said, we're also link, and also linking back to MM's comments earlier about kind of this pluriverse of feminism. (MM nods) I, I think it is also really important to be very aware of where you sit (JC nods heavily, MM nods), and where your own frame of reference to feminism is coming from. So maybe I can give two examples to explain this. We were having a conversation with a women's group in South Australia. They're an aboriginal women's groups that specifically serve their community, but they have partnerships with ehm predominantly white researchers from/ not no, not all white researchers, that's unfair. Researchers from one of, from Adelaide University, who are mixed of ehm Aboriginal and Torres Strait Islander ehm heritage and European heritage, and when we asked them if they considered themselves feminists, they categorically said No. Because for them feminism was this thing (MM nods and smiles), as kind of a desc/ description of JC gave that was about white, middle-class Australian women fighting for the rights of other women like them, and was actually deliberately very exclusionary. The conversation, and then so that's kind of one, so feminism is not something they identify with because it's not something that they've ever seen as for them. Ehm in a different context, our Kenyan colleagues colleagues, ehm a feminist organisation, and and there, to the the issues of race are different. So for them feminism is less about the the white ideal of feminism because there's just kind of still a/ there are power hierarchies, former colony, but in the majority ehm they're a majority Black nation. (MM nods) So the issues that they are dealing with when it comes to feminism with people who say they're feminist, whether they talk what the way they walk the talk are different. And so I think it's really dif/ it's really important to understand that the challenges that are being faced and the reckoning that needs to be done is as contextual as the forms of feminism out there, and without undermining or disagreeing with any of the points that JC just made. Ehm but again, colleagues in the South East Asian context say "That's your feminism, that's what you're dealing with in Europe and in the US, and in Canada. That's, that's not what we're dealing with here."

(CJ, JC, and MM nod)

[1:48:37.5] **HE**: (...) Yes, thank you very much for also giving these examples. Ehm, now regarding the time ehm I because I also want you to give the opportunity ehm because, of course, I don't consider every aspect that's important. That's why I also, I want you to talk a lot. Ehm I want you just to add if we missed anything very important, or if you would like to add something that we haven't mentioned at all, or that we only mentioned very shortly ehm which you would be/ which you consider very important for this discussion, so something you would like to mention that is very important for you on this topic.

[1:49:22.1] **CJ**: I'm not sure if this is already covered ehm in your ehm literature review, but it would, I would be interested to see it ehm in your ehm thesis ehm how diverse ehm health perspectives are in in values ehm, I don't know, at least from the Global South or Global North. And ehm just noting, I mean ehm, as earlier mentioned, ehm where do we start in terms of promoting alternative discourses (ER nods) that are, you know, that will challenge ehm the status quo. So if we, if we can ehm include that in terms of, you know, presenting it in such a way that there are ehm alternatives to the ehm, the dominant ehm global health ehm discussions. I mean, if even if we talk about global (ER smiles and nods), we also have to decide what does global mean. Ehm is global, ehm you know, the mainstream or the dominant discourse. (MM nods) And or what should it capture? So it could ehm align with your framework, which I think, you know, many of us here also, you know, resonate with, is really challenging, ehm that ehm dominant ehm discourse, I mean, that is white ehm, you know, ehm not really representative of the diverse perspectives. (ER nods) Ehm and so that would be something that I would, I would be very much interested to, to see. (JC and MM nod.)

[1:50:59.4] **MM:** I I agree with CJ, and I would be curious also to see, you know, who you cite (CJ and ER nod heavily) because that's very also important, you know, this hashtag #citeblackwomen, for instance, that has started like a few years ago in the US. Ehm because talking about yeah, gender health, global health ehm intersectionality, so who you cite is very important. (CJ and ER nod) Ehm, yeah. So if, I mean, if you want us to be involved as well, or at least me, I can speak for myself, but to be involved in your editing, like review/ reviewing your work, I would, I would love to do that as well. So yeah. (laughs)

[1:51:35.6] **ER**: Yeah, I think I'll second that one. And we've, as we've developed this kind of decolonial feminist approach, we've done a massive literature review and it really pushed

ourselves to look beyond the usual sources (MM nods), ehm and that means pushing ourselves out of our language comfort zones as well (CJ nods), you know, there's a lot of really deep and important thought that historically and still was coming out of Latin America. Ehm if you look at what afro-feminism looks like, ehm it's really very different. So, again, if you requ/, if you would like any assistance in kind of broadening your reference/ broadening your reference database, very happy to do so (JC and MM smile). And just ehm, just I don't have a specific point that I think we have been missed, I just want to say that it's been a really interesting conversation (CJ and MM smile and nod) that has given me much food for thought ehm about discussions that we're having internally for our own work as well. So thank you, HE, for the chance to participate and everybody for all the thoughts.

[1:52:36.0] **HE:** Yes, thank you also. Ehm, may/, yeah maybe JC, you go first. (laughs)

[1:52:41.5] **JC:** Yeah, this is a thank you. Ehm and just profound thanks to everybody, I've learned so much. (CJ nods and laughs) Ehm (...) yeah, this is a slightly adjacent thought, and maybe, HE, this is a different inquiry or a different project. But I think it would be really interesting to investigate and to acknowledge the contributions of feminism and feminist movements to ehm what are often described and understood and historicised as milestones (ER nods) in the history of global health. So whether it's the founding of the World Health Organization or the Alma Ata declaration on primary health care or the recognition in international law of the human right to the highest attainable standard of health, or the birth of the health and human rights movement in all of its manifestations. I mean feminists were behind, in front (laughs) of all of those (CJ and ER nod) developments. And so how we talk about that, how we narrate them, how we historicise them is so critical to ehm in a sense decolonising the field. (ER nods) (...)

[1:54:19.5] **HE**: (...) Yes, thank you very much for your ideas, ehm and I mean, as you know, I will definitely provide you with this video recording ehm and I'm also very happy to send you if I ehm analysed everything to send you my results (CJ, ER and MM nod), so that you can also see if it, if it really was what you meant to say if you feel understood, if it's okay. I mean I'm also happy to send you my my theoretical background ehm, but (laughs) I mean it's a lot (CJ, ER and MM smile), so I wouldn't expect you to read it, but if you're interested (CJ, ER and MM nod and signal agreement), I'm very happy to do so (MM holds a thumps-up), and I mean, of course, to get ehm a review from

your side would be very very helpful. Ehm, yes so that will definitely ehm happen. And yes, I can only say, thank you for participating. Yes?

[1:55:08.9] **ER:** HE, would you, I know you're having a second focus group with a slightly different emphasis. Ehm, will you be, I I know there's consent, but would you be in a position to share at least some of the takeaways of that conversation with us as well because I think it would be really interesting (CJ and MM nod) to see how they complement each other.

[1:55:28.3] **HE**: Yes, ehm so for sure, in my results, I mean I will I will just gather all the results together, but ehm I was also thinking, if I could just provide everyone with the both both of the videos of the discussion. So I would ask you if you were okay, if I ehm, if they are, (CJ, ER and MM nod) can have access to this recording so that you would also get it. I mean, ehm I would say it's okay for everyone who participated in all of my focus groups, and then you just don't give it to any third parties. Ehm, for me that would be okay ehm because yes, I think it, it would be interesting for all of you and I don't want to completely separate it ehm because it's also one project. Yeah, for sure. (CJ, ER and MM nod)

[1:56:15.1] **MM:** Yeah, thank you, HE, again, for the opportunity to think about this for two hours. I mean, I've been missing this kind of discussions (CJ nods) during Covid. Ehm yeah, and thank you because there were brilliant ehm interventions. (JC nods, CJ smiles) So thank you all who are still here. It was nice to meet you. (Everyone smiles)

(6)

[1:56:36.7] **JC:** Yeah, I feel the same. I feel a nice (smiles) connection between the group. (Everyone smiles) I really think doing this as a focus group worked (CJ, ER and MM nod). I felt just so ehm inspired and ehm prompted in a really positive way by what everybody shared, and never, never would have been able to say what I said without the ehm community that was created here (CJ, ER and MM smile and nod). Thank you.

[1:57:11.0] **CJ**: I echo ehm everyone some, you know, ehm positive feedback, and ehm feelings about this FGD. At first it was really more of, you know, ehm the typical research ehm, you know, another set of ehm interview requests for ehm inputs (ER, JC and MM smile and nod). But ehm, indeed, I can feel that ehm we really have connected (JC nods) and we share ehm the same ehm, you know, we are on the same plane (ER and MM nod) in terms of looking at ehm both ehm the problems and ehm well the ways where we can challenge this ehm, you know. Ehm ehm dominant structures. So thank you, HE, for the opportunity and

to everyone, I learned a lot from all your inputs as well. (...)

[1:58:05.0] **MM:** (MM sends a red heart emoji to the group). I know it's not allowed to share emotions ehm in (everyone laughs) in you know (laughs) this kind of field, but I just share love. (laughs)

[1:58:13.9] **ER**: Oh, somebody should have told me that a long time ago. (laughs) (Everyone laughs) Ehm, I have one request and I will not be offended if people said no. I'm just trying to look at the calendar invite to see if we already have the contact details of each other. (MM shakes her head) And but if not, I wonder if people are willing to share email addresses. (CJ and MM nod) I say that because it is this work that we're doing, and I would love to have the opportunity as this programme we're, we're working in very similar places to be able to continue to engage with people. I think JC and I probably have a colleague in common, in the form of Sofia Gruskin, (JC nods and laughs) who is like one of my favourite people in the world (laughs) (JC holds a thumps-up). But yeah, it would be really great to stay in contact, if possible. (MM nods)

[1:59:00.8] **HE**: Yeah, great, yeah if everyone agrees ehm I can all also give you the e-mail contacts. (CJ nods) I will probably ehm send an email to everyone so you can also see it there (ER and MM nod) But ehm yeah, or you write it in the chat now. Yeah, thank you very much ehm for participating. I also got so many new insights. Ehm I think it will be a tough task to all ehm gather it in my results. (CJ nods, ER smiles) Ehm but I'm yeah, I'm really thankful it was very useful for me. So I will ehm keep in touch ehm I will conduct the second focus group in a bit, and then it will, I will probably take some time, but I will keep you updated on everything and come back to you.

[1:59:42.2] **ER:** Fantastic. (CJ signals agreement and thanks, JC and CJ waive) Thank you very much, HE, and good luck with passing all the data. (smiles) (MM nods and smiles)

[1:59:46.4] **HE:** Thank you.

[1:59:47.3] **MM:** Yeah, good luck with the transcription. (smiles)

[1:59:50.0] **HE:** Thank you very much. Yeah.

[1:59:52.0] **ER:** Bye (waives). (MM waives)

[1:59:54.0] **JC:** Thank you all.

Transcript of FG2

Date of the focus group: 24 August 2022, 16:00 CET
Duration: 106min 13sec
Place: Online via the audio-visual tool Zoom
Moderator: **HE**
Participants: **OLU, SM, SC**

Transcription notation

**(…)**	Break up to 3 seconds
**(number)**	Break longer than 3 seconds, duration in brackets
**underlined**	Particular emphasis
**(nonverbal)**	Nonverbal expression
**(unint.)**	Unintelligible speech
**(word?)**	Unintelligible, assumed speech
**ehm**	Uniform notation of filler words (ehm, ah, eh)
**/**	Interruption of word or sentence
**//**	Speech overlaps

Transcript

1	[0:00:00.0]
2	[0:08:39.4] **HE:** Hello. (smiles) (5)
3	[0:08:43.4] **OLU**: Hi!
4	[0:08:45.9] **HE**: Hi OLU, hi SC! (smiles)
5	[0:08:49.3] **SC:** Hi! (...) Sorry I was a cou/ ehm a couple of minutes late. I just/
6	[0:08:55.0] **HE:** Don't worry! Don't worry, but you said the link was not working. Maybe I should//
7	[0:09:01.0] // **SC:** No, no. It worked, it worked.
8	**HE**: Ok.
9	[0:09:03.0] **OLU:** The one you sent over e-mail, it's working ehm the last one. I don't, I didn't try
10	the other one. I just send you the the consent form. (HE nods and smiles)
11	[0:09:11.1] **HE:** I saw it, thank you. Yes, thank you very much.
12	[0:09:13.8] **SC:** I sent it to. I sent it too. I hope you got it //

// **HE** : Yes, yes yes great. I got all of them.

[0:09:18.9] **OLU**: We're waiting for others, right?

[0:09:21.2] **HE**: Ah, yes, yeah, LDA should join, and SM wasn't sure if she can make it. But I think we wait another couple of minutes.

[0:09:29.5] **OLU**: Great I'm just going to grab a glass of water, and I'll be back here.

[0:09:32.6] **HE:** Sure! (smiles) Okay, great. (5)

[0:09:39.8] **SC**: So how many of us are going to be on the call, HE?

[0:09:44.4] **HE**: Ehm, I think it will probably be three and me. So LDA will join because SM was not sure if she can make it today. Ehm, so three or four plus me. (laughs)

[0:10:05.5] **SC:** Ok. But it's really nice that people were willing to move the time so /

[0:10:08.9] **HE**: No, no, no problem. //

// **SC** : Because we were supposed to do it yesterday.

[0:10:12.2] **HE:** No, I'm happy ehm that you're here, and also this way maybe this way SM can join us. So don't worry about it. Maybe I just send the link to ehm LDA again, so that she also got it.

[0:10:26.8] **SC:** Yeah.

[0:10:27.9] (20) (HE is sending the link again via e-mail)

[0:10:59.1] **SC**: So how is your study going?

[0:11:01.8] **HE:** It's going quite well. I had the other focus group//

// **SC:** How is your research going?

[0:11:06.5] **HE:** Yeah, I had the other focus group yesterday ehm. So it's a full week now. (smiles) Ehm, but yeah, I'm I'm quite happy.

[0:11:18.8] **SC:** Oh, nice nice!

[0:11:21.3] **HE:** So I'm just sending LDA a message. (16)

[0:11:50.3] **HE:** Okay (...) Yes, no, I'm ehm really content until now. Also that I got you here today, and that you're all willing to participate, yeah (nods) (5). So I would just see if LDA comes. But she said she will join, so she's probably just a bit late. (83)

[0:13:39.7] **HE:** Yeah, sorry you have to wait. Maybe we wait until ten past, and otherwise I just start and see if the others will join. (132)

[0:15:59.8] **HE:** Okay, I would just say we'll start because I will start with some introduction first, ehm and maybe hopefully, LDA will join us, and maybe also SM (4) (OLU turns on her video and nods)

[0:16:16.6] **SC**: Go ahead.

[0:16:18.0] **HE:** Ok, great. Ah, yeah, first of all, welcome and hello. Thank you very much that you're here. I'm really glad that you took the time ehm to participate in this focus group discussion. I also know I'm quite, I'm asking quite a lot from you also because it's early for OLU and late for SC. So ehm thank you that you're all here. Ehm, I also want to shortly talk about some housekeeping. Ehm as you noticed, I already started the recording. It was just easier this way in Zoom, and so I don't miss anything. Ehm, then I also sent you the formalities from my university in advance where you find everything about procedure and data policy, and you all sent them back to me, so I assume you know about it. But if you have any objections, then you can tell me now. (...) Ehm, then I also invite you to turn or keep on your video as far as this is possible. I think it's just nicer that if we see who we're talking to but ehm, of course, if your internet connection is not stable enough, you can turn it off. Ehm, so that we can lea/ can at least hear you. (SC turns on her video) Ehm, yes, hi (smiles). (SC smiles) Ehm, then also ehm, but I don't think this will be a problem in this group, ehm I ask you not to interrupt each other too much, or talk at the same time. Also because I will transcribe the sessions ehm. But we're two or three people, so this will be fine, and it's still meant to be a free discussion. So whenever you want to say something, you just go for it. Ehm, you don't have to wait for me to give you permission to talk. So it's more like a natural conversation. Ehm, yeah, and that's it about the the technical stuff.

[0:18:09.4] Ehm I also want to introduce myself because I only had e-mail contact with you before. (OLU and SC smile) So, as you know, my name is HE. My pronouns are she and her. I'm 25 old, and I live in Berlin. I study the masters Public Health and Political Science at the University of Bielefeld. And I study these because I'm especially interested in the intersection which is global health policy. And I also worked with some NGOs here in Germany, in the field of global health. And at the moment I'm part of a university project on decolonisation of global health. And more personally ehm, I like to do sports, in particular dancing (OLU smiles) or going for a run. Ehm, and I also enjoy very much ehm that I live in Berlin with my boyfriend at the moment, just to have everything this city has to offer around me. So yeah, that's it about me. Ehm, maybe now ehm you can also introduce yourself because I would like to get to know you a little bit more and to have it a bit more interactive. I suggest you just yeah, introduce

yourself. Ehm, yeah, maybe, OLU, you want to start?

[0:19:25.1] **OLU:** Sure (nods). (laughs) Hi, nice to meet you, both of you. (SC smiles) Ehm, I am OLU and based in Mexico City. Ehm (...) (laughs) I don't know what to say about me. But I'm a, I studied social communication. I, when I finished my my (laughs) my college degree I actually went to Berlin for nine months. (HE smiles) So I I also love that city a lot, ehm, but that is, I think, the longest that I've been away from my my hometown. Ehm so I'm, I've lived in Mexico City my whole life, and I am the Executive Director of an organisation called Balance, which means balance. Ehm the, we're a feminist organisation working on sexual and reproductive justice at the national level, but we also do some local stuff and also regional global ehm advocacy. And ehm, what else? My passions and hobbies (laughs) I (...) I don't know, I don't think I have a lot of hobbies. I (...) I have a cat (shows to the cat in the background which just walks in) (HE laughs), and I have a dog that I think has just arrived, and I need to open the door, I live by myself in an apartment and I have two sisters, that's a little bit more about m/ me, but it's not about hobbies. I enjoy a lot of ehm watching movies. Ehm, but I am also a movie geek, so I enjoy every kind of thing that it it's out there. But I am really a geek, and I get into this, ehm you know, like knowing all the names of the directors and this kind of stuff (smiles). (HE and SC smile)

[0:20:54.8] **HE**: Nice, thank you. Nice to meet you. I also have two sisters, by the way. (laughs) So yeah, SC tell us something about yourself.

[0:21:09.3] **SC:** To start with, I can't remember anything about movies, so I'm just the other end. (laughs) (OLU laughs) So I keep getting confused about who the actor is, and who the ehm, you know, I don't remember anything about directors and definitely not. Ehm, but okay, but I'm an activist I work on ehm issues around sexuality and gender, work with ehm largely the ehm LGBTIQ+ and sex worker communities. And ehm, in eh, especially those who come from socially and economically disadvantaged backgrounds, and ehm also those from small towns. Ehm, and we do have a range of ehm interventions with them, which includes ehm, you know, issues around rights and violence, addressing violence and violations, and ehm strengthening their own organisations, and also ehm social entitlements, accessing social entitlements. And health is also a component that we focus on, including sexual reproductive health, mental health, and primary health care. And ehm (...) yeah, and I love to listen to music. Ehm, I can't sing to save my life. But I love listening (HE

smiles), and ehm, I also enjoy ehm going and seeing old monuments ehm, like ehm, like some history, so so historical places excite me. So yeah that, that's roughly what it's about. Ehm so we work in many parts of South India and I've been doing this work for many many years. Ehm (...) ehm yeah and I live in the South of India, live in a city called Bengalore, which is in the South of India.

[0:23:05.9] **HE**: Yes, thank you. Thank you, both of you. Ehm OLU said she can still hear us, she just has to open the door. Ehm yeah, it's very, very nice to get to know you ehm and to yeah, have met you. Ehm I prepared a very short powerpoint presentation just to remind us why we're here today. What's the content, the expectations. So I would just ehm share my screen with you. (HE shares her screen and opens the power-point presentation) But it's basically what what's been in the in the concept note. (...) So you so should all see the presentation now. Ehm as you know, this project is part of my master thesis on the topic Feminist global health policy – addressing health inequalities through an intersectional perspective. Ehm, so it's an intersectional approach (HE changes to the next slide), and it focuses on the structural determinants of health, mainly on gender, race, and class. And this is based on the WHO framework on social determinants of health. And this framework influenc/ ehm emphasises the political context and the role of power regimes as these, then shape structural inequalities and discrimination. And I also give examples in my thesis ehm on how these inequalities affect health and well-being, and I give examples in the areas you can see listed there. Ehm and I focus a lot on gender inequality, but also consider racism and classism throughout all these examples. So if you want you can also ehm use these examples or others throughout the discussion. Ehm it's it's not necessary, but sometimes it's nice to specify some ehm aspect. (HE changes to the next slide) And then I also ehm, as you can see here, my understanding or definition of a feminist global health policy. But this is based on the literature. So maybe you have a different definition, or we end with a totally new understanding. Ehm but we can use this maybe just to have something common to start with. Ehm so I understand feminist global health policy as a very holistic and intersectional approach, so it should be inherently decolonial. It recognises the importance of the socioeconomic-political level, and it also aims to challenge and change power hierarchies and the resulting discrimination. Ehm and it does so by focusing on the most marginalised first, ehm and by shifting to more participation and mutuality. So in my thesis I aim to create a framework on a feminist

global health policy and how it can be best implemented as far as this is possible. And I use the focus group discussions ehm for my results. So for (HE changes to the next slide) the discussion, I would like you to consider ehm the aspect of intersectionality and the role of power regimes. And also as a very broad frame ehm I have this What, Who, How aspects that we could cover ehm in the discussion. So I can (HE stops the screensharing) stop that there. Ehm I have one more ehm important aspect I would like to mention. Ehm because you know that I'm adapting feminist research methods and that I'm using focus groups so I remain rather in the background and the focus is on you. But I'm also aware that this is not a perfect project. Ehm and I am a white woman, I come from a Global North institution, so I'm a very privileged person. And of course I want you to use the results, and also to to benefit from this discussion. But ehm of course I also know that ehm this primarily serves my master thesis. So I try to be be very reflexive about it, and also to include these thoughts in my thesis. Ehm but I know that it's not a perfect project and that there will remain some power hierarchies.

[0:27:06.2] Yes, so ehm. Having said all that ehm, I would actually like to start the discussion now. Ehm, I think we just start ehm with the two of you, I don't think it's a problem. Ehm maybe it would have been also nicer for you to have more people, but ehm. Yeah, I think it will be interesting, anyway, and maybe others will still join. So I prepared some guiding questions. Ehm but, as I said, the focus is also on you, so ehm we can also see th/ where the discussion takes us. If we consider the What, Who, How aspects ehm. Yeah and also I want to stress, but I think ehm it won't be a problem, that this is a open and non-judgmental discussion. So there are no right or wrong answers, ehm you can disagree with one another. And also ehm I don't intend to find final answers. Sometimes it's even more of a value if ehm new questions emerge. Ehm yeah. So ehm ideally, I did all of the talking by now. Ehm. And that we can begin, I also sent you the first question in advance. And this question I would like to get an answer from ehm the both of you, and then ehm we can have more open discussion, where you just jump in when you want to say something. So I want to start by asking, What do you consider the most pressing structural challenges regarding global health policy? So at the moment. Ehm and maybe you think about a lot of structural challenges, but I just want you to consider the ones you think are the most pressing ones. So ehm I don't know if anyone wants to start, or I should choose someone. (smiles) (OLU and SC smile) (...) Ok, then SC you, you go first if you want to.

[0:29:04.6] **SC:** Yeah, fine. So I think in India from an Indian perspective, I think of, one of the big issues we face is that the state has ehm (...) you know, I mean not that the state was doing too much in health, we've always had a problem. But even whatever there is, is retreating. So, you know, there is more privatisation of health that's happening (OLU nods), at all levels. And ehm this ehm, of course, has various kinds of implications fo/ on health, because it has implications for access, it has implications for affordability (OLU nods), it has implications about what kind of treatments get pushed, and what get ehm left out or neglected. Ehm, it has implications for ehm, who gets it, who will get access (OLU nods), you know, which which class of people, which location of people, which kinds of people. So (coughs) and of course, ehm because it's getting more and more privatised then it is not, it/ you know, it it's getting diluted as a right. Because as a private person there is something that you can decide who you want to give this service to. (OLU nods) Yeah I mean you have that (expertise?) whereas with the government you are supposed to get it as a right. So in my view this is one of the biggest challenges that we have.

[0:30:24.4] **OLU**: Yes, I I think of it that is happening also in Mexico. I (...) I I was going to say neoliberalism, as the structural (laughs) barrier because that is a framework right. Like ehm, and it's this idea of universal health coverage instead of universal health access. That I think that is, is, by diluting ehm the right to have ehm well, the Triple-As-Q for for for healthcare, no. The accessibility, affordability, ehm I don't remember the other one. Access, I don't know. No accessibility is the other one. (SC laughs) I am missing one A, and quality. Because for example, in Mexico the thing/ the same thing has been happening over, I think that for thirty years there's been a a campaign on how public services are very bad. So people are like going scared of, of of of those public services and going to the private services, thinking that it's going to have more quality. But the reality is that there's no one that is really or truly ehm (...) regulating or evaluating the the kind of services that private hospitals are are giving. So it's it's very privatised. And so any any rights violations that are occurring at the private sector, is really hard to follow up on that. I think that there's another challenge also in terms of criminalisation. And that, don't get me wrong, I I totally think that there's that there's ehm obstetric violence exists. Ehm but I think that there is this ehm. The risk is that, for example, for obstetric emergencies, doctors don't want to pitch in into the into

getting into the emergency and have their hands on on someone. Because if that happens and the last person that's going to be ehm attending the the woman if she dies, and that is like when emergency occur that's that's what one of the the possibilities that is the higher, is that she will die. Then they don't want to have their name on that record, because so/ then they will be the ones that that will be punished for the obstetric violence. Even if it if they were the ones that were trying to save her at the at the very end. And not the people that were neglecting her before. So I think that tho/ those are kind of a risks that are very specific, but I think that that are happening, and that (...) in a country like Mexico that had a lot of effort on public services, and we have different systems of public services, and we have rural ones and and these are the kind of the specificities that are happening that are also keeping, keeping women away from from having quality ehm care. And that falls on on us beca/ on us as a feminist movement because we have been pushing for that agenda on criminalising ehm neglecting doctors. But I think that we are missing how to do it in a way that is not affecting them.

[0:33:17.8] **HE**: Yes, thank you both ehm very interesting and very important aspect. Ehm so now we mentioned, like the most pressing challenges and the more the problems. So now I want to go more to a solution-based focus. What do you think, what alternatives could a feminist global health policy provide? Ehm, maybe to the examples you gave, but also ehm in general, what what you consider, what benefit would it bring?

[0:33:51.3] **SC**: I think if (coughs) I mean I think the the it would have to be ehm about going back to ehm see it as a right doing so. (OLU nods) We establish back that health is a right and ehm, you know, and and and right with all kinds of other things. That means you need access to non-judgmental, appropriate healthcare, which is in a way with (...) consensus-sought, you know, where inform consent, the, you know, the per/ the person can give <u>informed consent</u> in those situations. And ehm so and I think the only way in India that you can actually have all this doing is through community actions. (OLU nods) And ehm, so only when communities are more organised and ehm can come together that they can a feel a sense that they have a right to the health. Because oftentimes women I mean, like in many most parts of the world ehm neglect their health. Women's health needs come ehm very late because ehm, you know, mostly it's about it's about not just money, it's also about time. Because if she's going away then, you know, there's other chores that somebody else has to pitch in and do. And also (OLU coughs and apologises) you find that there's

no, you know, usually women are the one playing the accompanying role. That means they're accompanying men, they're accompanying children to the hospital and you, you know, you have to wait for a long time and they're doing all that. Ehm but nobody is there to accompany her when she's ill. Ehm so for many reasons, and women are also taught in India that, you should not be whining and you should not complain (OLU nods) and you should you should be the one who, especially if you become a mother, then, you know, then you have to look after people, look after, you know, either a wife and a mother, at least before that maybe there's some tolerance but after that now you're a whole person really, you know. So then you have to look after your husband, you have to look after your children, you have to look after your in-laws. So you can't be the one who's complaining and whose, you know. So all of this means that (...) that you fe/. So establishing rights is to you yourself to understand that you have a right to get this. Ehm it's not only that the state doesn't give you and all this. And I mean, of course, all that is there. But (coughs) even for the woman to feel that I have a right to look after my health and I can, you know. So it's about that. And it's about therefore looking at examples where communities have gotten together and started to press for rights and those ehm, you know, taking more control. Ehm, there's also a difficult a line on traditional healthcare, you know. Because ehm on one hand, we have systems of healthcare in India like in many other parts of the world where, you know, which are non-Western forms, of healthcare, systems of healthcare. Ehm which on one hand, you know, you feel that you want to encourage, you want to support, and because it's more, you know, it's less doctor-centred, it's less pathologizing, it's less ehm, you know, less expensive, it's less all of that. Ehm but, on the other hand, ehm (...) the flip side is, some of those things are very harmful. Ehm, some of the local traditions are very good and positive, and ehm, you know, and and the thing, but some of the others are harmful, and ehm, and some are harmless, some are harmful. So ehm, you know, how do you sort of ehm deal with that. Ehm, and I see SM has joined us. So, it's nice. So /

[0:37:42.3] **HE:** Yes, yes, she just joined. (smiles) //

// **SC:** Yes so that's something /

// **SM:** Hi everyone.

[0:37:47.4] **SC:** Hi SM.

[0:37:49.3] **HE:** Hi SM, thank you for joining us, I know you have a very busy schedule.

[0:37:53.8] **SM:** I do. (OLU and SC smile) Because I'm training in the Eastern Cape and I don't know how long I can be here for. But I'm just joining and listening in. And sorry I won't put my video on. If that's okay.

[0:38:04.6] **HE**: Yeah, sure, that's fine. Ok. Sorry, SC. Ehm, just go on.

[0:38:10.4] **SC:** Yeah, that's I was just trying to say that with ehm, you know, local traditions of medicine. Ehm, there's on one hand, it's a tendency as you're talking about decolonising, there's a tendency to see it as a lower tradition like, that it's less than the Western ehm kind of traditions of medicine (OLU agrees), and ehm, you know, and that's not true, because it's sophisticated, and it's so way it's complex in its own way. It's, you know, just a very different system. That's it. But on the other hand, some of the ehm local traditions are very harmful, but then some many of the allopathic practices are also very harmful (OLU nods), you know. So it's not like what in India we call Western medicine or allopathy. It's not like allopathy is like (breaks?) is some, you know, some of what/ things that because big pharma pushes a lot of stuff which we know is extremely harmful. Ehm so it's it's, you know/ So navigating that is a bit difficult, I mean, I don't know, feminists having ehm having the position of yes or no to ehm local, local medicine. Ehm but there have been efforts, for example on the whole midwives and, you know, how (Orina agrees) and the (unint.) got institutionalised. What did it mean for people who worked as midwives, you know. (OLU nods) Ehm is there a way that they can get into the system of institution (...) child ehm, you know, child births in in in in hospitals and institutions. Ehm (...) because lots of women were dying (OLU nods) of ehm maternal maternal mortality was quite high. Ehm, you know, ehm because they, they neglected and other things. But at the same time it's not like traditional birth. Attendees didn't have ehm knowledge, you know. So you're facing that completely and it's also problematic. So yeah, so it's it's. (SC signals with her gesture that it is a complex situation)

[0:40:07.8] **OLU:** Yeah.

[0:40:08.7] **HE**: Thank you. And I will just ehm say, SM, we were just talking about what alternatives ehm feminist global health policy could offer. We were talking about like the major structural challenges in global health at the moment. So ehm, just let you know. And ehm yeah, OLU can say something, and maybe you can also then jump in, if you have the time.

[0:40:31.7] **OLU**: Yeah, I was just going to say that it's also about the definition of health, and I I

mean like, yes, health as a right, but also health in a more holistic approach, and making sure that when we are talking about health we're not talking only about, you know, basic healthcare, but also that we are talking, you know, in a more, yeah comprehensive, integral package that that that is also about prevention and promotion of health. And it's, I mean, it's not just about attending when somebody has a disease. And that's why I think that the definitions are important in ehm in how how are we seeing what health means. And I think that a/as as SC said, there's something about communities, that is also providing health and there's a comm/ you know this community of care and they're many things that are many things that are happening because there's a community. For example, abortion in in Latin America, it's it's very criminalised. And and there's this abortion doula that arrive, like abortion accom/ companions. That is basically civil society and feminist movement organising to be able to support women ehm through through their abortions. And it's and it's quite safe and I think that WHO ehm new technical guidelines are now including the community, ehm the community aspect of it, and how the the the community providers are are supporting that kind of access, and I think that that is relevant in in terms of how do we see ehm more like an ecosystem ehm that is working around health. Ehm I think that it's really important to talk about budget. Ehm national budgets and how we don't go back from the from what governments are giving to health and to education, and all these basic stuff that we need. Ehm and redi/ redistribution, of course, and how we talk about taxes and all this other stuff. And ehm (...) and for sure, how do we talk about militarisation, and how much money is going into that. And why are we still like going through that approach? Because I think that at/ I mean Mexico, as as you probably know from from the news is a very violent ehm country in like cross-cutting. And it can be blamed on specific actors, but I think that it's also about how institutions are becoming more and more violent. And having ehm now they're having, because of gender equality, they're requiring also women to do the military service. They're they're promoting that bill right now. So this kind of of like weird ideas of what equality means, and I think that one of the the most (...) radical things that we need to see is that gender equality means a lot of of of improvement on how do we ehm place health. And how do we place specifically sexual and reproductive health. How do we ehm give that control of the women so they can also control a little bit more about their lives, and their bodily autonomy.

[0:43:32.5] **HE:** Yes, thank you. Ehm, yeah, SM maybe you can also add on the alternatives a feminist global health policy could provide, maybe also by focusing a bit more on ehm decoloniality?

[0:43:48.6] **SM:** Ehm yeah, sure. Ehm, so (unint. due to bad internet connection)

[0:43:59.9] **HE**: Sorry I can't really/ Ah now, it's working better.

SM: Oh, can you hear me now?

[0:44:05.9] **HE:** Yes, yes, now it's good. Yeah, sorry for interrupting.

[0:44:09.0] **SM:** Yeah, reception is really bad. I'm going to switch my video off. Ehm, so what I feel is that ehm we have to ehm, we have to acknowledge the history. We have to acknowledge the past. We have to go back to the history and to understand ehm not, not what happened ehm because we we know what happened, but I think we need to understand the technologies that were employed by the colonial project to racialize and gender ehm subjects in relation to whiteness. Okay. And in relation to the white man, the white European subject. Because ultimately the white European religious subject, (OLU smiles) actually and and the list can go a little bit longer or deeper, but I think the Othering process is in relation to an idealised ehm ehm, a human. (OLU nods) Ehm and ther/ there is human and then there is the Other. And the human is not even a woman. The wo/ the human is a male body. It's a male subjectivity. So if we aren't able to go into the technologies of how domination and control operated, we're not really going to get to the belly of the beast. We are not really going to tackle the problem. We're going to look at it as a peripheral thing. We're going to try and make adjustments in policy. But actually, if we go to the heart of it, we can see that in in in the South African story, in the Indian story, in the English colonial story there needed to be a creation of the of of the of of race of of this kind of hierarchy. So the creation of of the racial subject, so the Black person, the the Indian person, ehm the Chinese person, the/ and in South Africa we only have four racial categories. It's Black, white, Indian, Colour. And then it was the creation of the racialised subject, the the gendered subject. So in in that process they ehm understood that ehm family structures and women are the heart of the problem, or or or hold a lot of power. And ehm and and by ehm by, you know, dehumanising the woman (OLU nods) and taking away her power, they had the power to control ehm, you know. So I'm laying that foundation because I think perhaps we don't understand enough of the problem before we go into the solutions of poverty, I mean, of of policy making for for the problem, right. And

so I I I mean, I know I'm entering the conversation half an hour late so perhaps that/ the groundwork is laid. But I think that until we understand those technologies of (governing?), and how they intersect and interlink with capitalism. So racism, capitalism, and patriarchy ehm (OLU smiles) anything that we're going to do in terms of feminist policy ma/ne/ needs to take into account those things and needs to intervene at the point where we're not just thinking about ehm ehm women ehm as a as as a as a universal issue, but rather the intersectional issues of which kind of feminist policy we're talking about. Because in South Africa feminist policy has been critiqued because we know the issues of white feminists (OLU nods) versus, you know, the different levels of feminism.

[0:47:55.3] And then, if you go back to the core or the heart or the history, the history of sexual reproductive health and rights. We know that Margaret Sanger (OLU smiles) and Marie Stopes are controversial figures (OLU nods), white feminists, radical white figures, who brought about contraception and promoted women's rights and promoted the use of contraception, you know, but only for particular types of people (OLU nods), and, on the other hand, supported your eugenics. That would actually literally, through a genocidal process eliminate bodies, like the three of us on the score, you know (laughs) (SC smiles, OLU nods). Ehm, all of us as People of Colour, those white feminists had an intention to remove us from the earth, (laughs) to wipe us out (SC smiles, OLU nods), you know. So I think you can't go into feminist policy if we're not taking seriously the history. And I think this is what the decolonial process is trying to draw our intention that actually that they/ that colonial polic/ feminist policy, we need to examine the language because there are epistemic injustices (OLU nods) occurred in policy that policy making in the global im/ imperial design is ehm is is a colonial process still. It's a it's a it's not necessarily ehm trying to achieve ehm social justice or repet/ it's trying to achieve a particular type of fe/ social justice that appeal to a particular type of feminism. Not necessarily the feminism of the people where I'm sitting, which is rural Eastern Cape, where our maternal mortality rates are astronomically high, where ehm women and men are ehm are considered unemployed, but they're actually subsistence farmers, and they operate outside of the formal economy, you know. Where they do use doulas, and they do use birth attendance, and there's a whole different infrastructure that's not existing within the the the Western imperial design project. They're outside of the system already, and we know in India that they are communities that

exist like that, too. So feminist policy for whom? (OLU nods) You know, feminist policy to achieve what? Ehm and in whose interest? And who is the/ Who is the policymaker? Let's ask those questions. Ehm and how are these policymakers really going to get to the heart of the issue. And are the these feminist policies going to re-create ehm the same problem using the same technologies? Or are they going to actually ehm in some ways introduce new power dynamics. Ehm because right now we see that the that representation is shifted, and you don't only see ehm particular faces or particular types of people in in policy spaces. They are women of Colour. They are women who are in powerful positions, but they they use the same ehm technologies (OLU nods) of domination and control. And we're not going to achieve, just through representation, ehm a level of policy-making that's going to achieve social justice. Yeah. So that's my intervention.

[0:51:18.1] **HE:** Yes, thank you very much. You touched on so many aspects I think we can ehm further discuss. Ehm and also maybe this this fits to it. Ehm we talked about who holds power at the moment in global health ehm at the beginning we mentioned privatisation, neoliberalism and now we added the the colonialism and coloniality. So (...) what are the necessary steps to to transfer this power? And the second part of the question would be to whom, if we really want to make a difference. And if we really want to be truly intersectional feminist policy. (...) So yeah, whoever wants to answer ehm can, do so, or just also one aspect of the question. (5) (OLU smiles)

[0:52:12.1] **OLU**: I feel like that I need a/ Can you say it again?

[0:52:15.5] **HE**: Yeah. (smiles) Yeah, it was just ehm ehm what are necess/ like, what can we do to transfer the power from those who have it at the moment in global health and to whom should the power be shifted? If, if there is a possible answer. (6)

[0:52:38.6] **OLU**: I feel like that for me it's a definition is what is global health. But ehm (laughs), and because there are, there are many ways, I mean when you're doing global advocacy, and you're sitting with a with representatives of different states. It's really tricky because I think that it is hard, because you know that there/ there's a lot of, even if if there's People of Colour that are that were there, and we're trying to make our case. There's definitely more ehm white women or white men trying to do that part of the of the work. And it's really counterproductive for them to be approaching governments from Africa, governments from South East Asia, you know. Like why are they the ones that think that they have the best way on talking. And//

[0:53:23.1] // **HE:** But you can, you could also think about ehm/ I totally agree with your point, but to to think about at the local level, about health policy in general. So you can also adapt it to your context of course.

[0:53:35.9] **OLU** Yeah yeah yeah. So I think no. But but I think that it it goes at all level. So I think that there's there's one level of discussion there, that has to do with what SM was was saying, in terms of how do we, when it's it's suspicious when these white women are talking about abortion in my country, you know, like these kind of things it's like why are you talking about it? It sounds like you don't want more indigenous people being born, or this kind of of narrative, so it it pushes back. But even when you get some agreement at the international level, when it comes into the country, there's still a lot of racism that's happening. Ehm for me one of the best programs in in Mexico is the rural program that I was talking about before. And it integrates, for example, the the the midwives, and it integrates them into the into the whole institution, and how they/ because they realise that women were not coming to the hospitals to have their their births because they were not able to take their placenta to to bury at home. And these kind of things that are traditional to them and that needed to happen. So now it's, there's a lot of more integration into that. But it's still that same institution. It's only giving ehm services to women that respect the two kids rule. So if you're an indigenous person, you only are allowed to have two children, and that is still completely racist, and it's about controlling. So I am I am just thinking I don't think that there's an a specific answer, and who to/ how to change the power. I think that there is a part in the universities, ehm in in how we teach doctors what the role is. I think there's a lot of hierarchy happening there. I don't think that they see themselves as facilitators of techniques and knowledge that I think that they they see themselves as judges and decision-makers, instead of of of allowing them to, you know, like to give the the specific tools for them to to yeah, for for the people and for the patients. I think there is something that got/ gets a lot of hierarchy there and then it just gives away all the power away from the patients and into the doctors, and it's still happening. It doesn't matter if the doctors are female or male. There's a hierarchy there that shouldn't exist.

(6)

[0:55:52.8] **HE**: Thank you. Someone else wants to add on that or to bring forward a new aspect? SC?

[0:55:59.5] **SC:** Oh, yeah. So I wanted to talk about the grassroots-level workers that we have in the

country. Ehm who are basically the backbone of ehm our health, you know, all the progress that has happened on health. And these are women from ehm, you know, they called Asha workers, and they are ehm, they form the backbone of the, as I said, they form the backbone. And they, you know, they have taken on the/ We have a National Health Mission and they do most of the work, a lot of the work that's done at the village levels, reaching women, encouraging them to get into, you know, whether it's about vaccine, you know, giving the children the vaccinations, or whether it is ehm (intuition?) the delivery or, you/ a range of other things that they track and take care of. And ehm unfortunately ehm they are one of the largest (unint.) I mean it's perhaps the world's largest ehm, you know, health (unint.) So, you know, I mean it's it's a huge number that of people that/ women who are who play this role. Unfortunately (...) they are burdened with a lot of work ehm, (OLU nods) lots of things that are just dumped on to them, and they they are considered as volunteers. So they are not paid a salary. They are paid some small kind of honorarium. So there's a lot of ehm, you know, so it's it's again on the backs of poor women that the health system is sort of built on. And ehm so, you know, and I think ehm if we are building policies then we have to build by having us/ having them at the centre. Because they've been doing this work. They've been struggling and doing and reaching very remote areas in India. But you know, in all kinds of ways. So one needs to have conversations with them, that's respectful, and ask them what is working, what is not working, what would help, what is the situation? Ehm it cannot happen the other way round. It has to be built bottom-up slowly. (...) For most things, there may be one or two/ a few things where, you know, maybe you can have more centralised whatever, but for most/ some research or something. But most other things you need to have a bottom-up approach. (...)

[0:58:27.7] **HE**: Yes, so ehm, what are the challenges in including these grassroots-organisations, or community-based ehm movements? So why, why is it not happening?

[0:58:41.8] **SC**: Ah, many, many reasons. (OLU smiles) I mean, first of all, there's a tendency to see them as, you know, the split between the thinkers and the doers. (OLU nods) So they're visualised only as doers. That means you have to do/ you you design the programme, and they have to just go and do it, kind of thing. It's a very limited view of what we think of, you know, the capacities of people ehm who are not in formal kind of institutions or universities, or whatever, you know. Ehm so there's that, there's this this kind of thing that happened. Ehm there is, of course ehm ehm vasted lots of vasted

interest to keep things a certain way, you know, ehm so you know, and it's not just corruption and all that. It's a far deeper kind of thing of the valuation of what is valued, what is ehm thought of as the best solution which is often like, you know, ehm sort of lopsided, you know, and ehm. So it's it's it's, you know, so it's all of that that (coughs) plays a role in ehm making it difficult for ehm, you know, people who are/ because, you know, they/ their knowledge is not acknowledged or counted as knowledge, you know. It's not valued as/ they're just seen as foot soldiers.

[1:00:12.2] **OLU**: And there's a lot of ehm, there's a lack of (...) political will to do that, and there's a lot of (...) waiting to like all, from the moment that they understand all the technical parts, it's going to be already too late. As if, I don't know, there's not enough accompaniment. I was also thinking about this kind of ehm civil society mechanisms, and how ehm to bring in people from different communities, you/ young people, indigenous people, people from outside the capital, these kind of things. And there's a little effort on on to give a/ giving accompaniment to them. So it's just, I I think, that people are set up to fail in these kind of mechanisms also because they're not giving enough of the resources to be able to participate in a meaningful way. So it's just like having this tokenistic approach in to like pretending that they are being intersectional, or they are ehm I don't know, doing that part of the of the work, but they are actually not. Ehm not giving enough for for people to be able to fully integrate into the decision-making.

[1:01:22.0] **HE**: Yeah, thank you. So what would be if you could ehm pose some, what would be your demands to the, really like the political decision-makers? Ehm what would you expect from them to improve the situation?

[1:01:36.4] **OLU:** To resign. (laughs) I'm kidding. I mean, I'm kidding, and I'm not kidding. But ehm but that is that, the thing right? Like as long as they, I mean, it's this idea of like, if you're not part of the solution, you're part of the problem. If you don't have the political will, and you're really not ready to see why it's needed to have this this this power shift. Then it's because you probably are the ones that are gate keeping or blocking that from happening. So ehm, I think that there's for me this this idea of, maybe very naive, of what a public servant should do, and is caring about the communities that they are attending. So there's there's a part there that I don't see that people are getting the jobs because they care about the community, that it is, I don't know, heartbrokeni/, heart-breaking. I don't, I don't understand how (...) Like, if they, if they only care

about working eight hours per day, or these kind of things or getting, I don't know, a pay check, why are they doing the public service then?

[1:02:44.4] **HE**: SC, what would you say? What would be demands to the political decision-makers?

[1:02:49.2] **SC**: Actually, I would also say resign, resign. (laughs) We have, we have a very awful government, but anyway. (OLU laughs) So ehm that's a topic for another day, another conversation. But ehm (...) Yeah, but I think ehm the idea would be to stop this ehm, you know, we have we have these workers for this, who are doing this. So if we can have, and they're all over the country, so if we can have a more decentralised approach, and an approach that is far more respectful of different ehm traditions, cultures, ways of understanding health (OLU nods), you know, which goes beyond the hospitals and goes beyond the pathologizing and that kind of attitude to health, and actually then we can have, you know, and then there can be networks, they can learn from each other. Community learnings can happen, you know. Ehm, you know, from grassroot-level groups to the other grassroot groups, et cetera. And a lot of other things can happen if if if if we move to a more decentralised kind of approach to health rather than ehm, you know, just looking at it in this way, and also stop seeing health only in terms of hospitals and tests (OLU nods) and medicines, and you know all the rest of it. So ehm you know, it's just the way that health is viewed itself is still narrow and ehm also that the people who have been doing the work for so many years, and have the year to the ground, are not respected. They are used in a very instrumentalist kind of way to deliver something. So ehm if we up-turn it and we have, you know, information flows from them. And of course, in conversation with doctors and scientists and everything. But in conversation and dialogue with, not, you know, not not not not just they would give you, they tell you what to say and you put it down. (Something buzzing in the background)

[1:04:54.1] **OLU:** Yeah, I think that this is still (...) I mean the part of the budget is is one of the things, too. And that part for me is a little bit tricky in terms of how decentralising ehm healthcare in general because when we decentralise that and the states get to say what they're going to use the budget for it, then it becomes a little bit more tricky. There are definitely parts that need to be decentralised, but it's still like how to push back on privatisation. I think that there's there's a part there that is not responding to the interests of People of Colour. Privatisation responds to the to the interest of of white people. So ehm (...) Yeah, I think that/ And then it also becomes not about health, but

it's about profits, and, you know, like changing the whole, the whole system in terms of like, how do we think about things? I I think that sometimes people don't want to create a new system. And I think that it is needed to create a new system and to think outside of the box. And and remember what public (smiles) health, you know, services mean, and and why/ how is the only way that we can actually say that it's a human right. Health is a human right only if we all get access to quality services.

[1:06:15.1] **HE**: Yes, so ehm if you were to create a a new system, ehm who would be accountable? Like if we consider the aspect of accountability, who would be responsible ehm that it all works in a better way. Ehm I mean, of course, we mentioned that civil society, especially the grassroot-level, needs to be included, but who would be actually, ehm yeah to be held accountable.//

[1:06:40.1] // **OLU:** Accountable. (HE nods)

[1:06:41.9] **OLU:** The government. (laughs)

[1:06:45.1] **HE:** After after the old one resigned. (laughs)

[1:06:48.4] **OLU:** After every else went, yes. (...) Ehm (...) I think that there there needs to be a system of governance, ehm and also create this kind of a state definition that it's/ it's, state is not only government, but rather that we all take par/ part of what the state means. And that means that we need to participate, we need to monitor, we need to evaluate, as well as. As as, you know, as, and to be able to to hold them a accountable. If we don't do that, we're going to be held accountable (smiles) from from the community in not doing that part of the job, you know. Like there is a chain of accountability, that it that that needs to happen, and I and I think that yeah, I think that we are, we as a (uses air quotes) feminist organis/ organisations or civil society, that is organised, are accountable to our communities. And I think that that is a a chain ehm and then we are holding accountable the government, and we all/ all this chain (underlines her speech with gesture) creates a state that is more aware of what's happening, and and that means the government needs to be more transparent, and then there/ there needs to be governance ehm systems in place for for that to happen.

[1:07:56.6] **SC:** I think also that it's time that we also held eh corporates accountable because we know that, you know, an awful lot of stuff that has happened, and what they have pushed, you know, Big Pharma (OLU nods) has really ehm done a lot of damage. So I think it's important. And also to ho/ hold multinational organisations, you know, the the whole ehm,

for example, international finance institutions (OLU nods), you know. And we saw through Covid also that, you know, what happened, or what didn't happen. Ehm, you know, what what lo/ a lot of things around the vaccine and who got and who didn't yet and who was pushing what and, you know. So a lot of the politics and I think including WHO, there is a need to call them out and trying to, you know, and ehm (...) have more accountability even there. I mean it's one thing//

[1:08:50.3] // **OLU**: Yes.

[1:08:50.7] **SC:** Yes, the governments, but also these bodies, you know, I mean ehm somehow we seem to think that if it is a World Bank or a WHO, then they are sort of the final authority and experts are very unbiased. But it's not. You know, they're not. I mean, we know what they've been pushing for so long. We know the kind of things that they've been doing (OLU nods), you know. And I think it's time to call that out quite openly. And you know, say this and not treat them with kid clubs anymore.

[1:09:24.0] **HE**: Yes, thank you. Ehm, so (...) you also mentioned that WHO, multilateral institutions, so ehm if we include this this aspect of global health, like really the global scope ehm (…) Is it actually possible to to have like this global holistic framework of a feminist global health policy? Ehm is it possible to agree on universal components? I mean, you mentioned in the beginning that it's really difficult because it all has to be context-specific because we all face different problems, issues and ehm you don't want me coming to you saying what you should do about abortion in your country, of course. So I'm thinking, is it actually possible to ba/ to agree on basic, basic aspects that then have to be implemented locally. And ehm, yeah, I just would like to have your opinion about it, and maybe like, what, if yes, what could these aspects be? Ehm, and if possible, I would also like to get SM back into the discussion if she's able to do so. (smiles) (8) She's muted, so maybe (...) she's just listening. (5) Okay, so maybe someone else starts.

[1:10:59.5] **SC:** Ehm, (...) Yeah, I mean it's it's it's a big question. But ehm (...) I think efforts have been on, I mean to to to define some principles. It's not, you know, what what what can get you (unint.) agreed upon is some principles of overarching frameworks. It's not ehm, you know, it's not so much ehm particular because those have to be developed at the grassroot-level, and it has to be contextualised, and it has to be/ it has to make sense, you know, for you. But certain sort of broad principles, broad frameworks can be ehm developed, but it's important that those frameworks take into consideration (...) the

local the, you know, and the state level, the regional level, at the international level, and and how these linkages can be established. Because ehm it's ehm, you know it's ehm, you know it's both like sometimes you're frustrated about working at the very local level, because so/ some big wave comes in, you know, through ehm (NIFI?) or whatever and this sweeps away whatever's been done, you know. And ehm at the same time, ehm if you're working only at the international level, then it seems very removed and very distant from what's happening in communities. So how do you build the framework which sort of allows, you know. So it has to be ehm, like with all feminist stuff, it has to be flexible and (more important?) it has to be work in progress and you know, we keep, we keep allowing ehm, it's not like, there's no Bible that they're going to be writing. (OLU smiles) Something that we, this is it. But it's going to be something that allows people to take what they want, add, eject, shape, reshape, you know. It has to be (...) ehm, you know, like clay or plastic, whatever, you know. Like clay where you can keep moulding and re-moulding it. And that's what we what do, what broad principles and frameworks (unint.)

[1:13:15.3] **OLU**: But ehm still there's some kind of teeth that are missing from those kind of frameworks, you know. Like, I I think that we have, I mean, we have the human rights declaration, (laughs) this is quite broad, but it's a it's the principles for everybody. Ehm, we had like the the Platform of Action from Cairo, that was the framework of how to do things and because it was a framework, and it didn't say exactly what it (...) / how it needed to happen, there were A) a little bit of some rights violations that were happening, for example, in Peru, where there they they still went on with the population control kind of way. And, on the other hand, when people/ when governments saw the possibility of of having the the millennial development goals, then they went to like, "Oh, these are more specific. Then we agree to these, and we forget about the framework. Ciao." And I think that, I don't know how to say it (laughs), but I I agree with this framework because the framework gives you more about the (...) yeah, the principles and the under/ undershell and then you you can decide a little bit more in in like what makes sense. For example, right now, the Agenda 2030 I think it was, it was done on a / it it's really hard to create goals that are going to work for all of of the countries. (SC nods) Because, I mean the old targets and indicators are very, some of them are like unattainable, and some (laughs) others are like that was easy, ciao. (laughs) (SC smiles) But ehm, you know, like they're not relevant because it's not a challenge for some countries. But ehm I think

that that the thing is, how do we keep seeing everything as a as a more (...) whole approach, and how, I mean, like the financial agreements affect the climate agreements and affect the health agreements, and how that all is a little bit intertwined, and I would say, like, we still need to talk about the common but differentiated goals on how how do we keep holding accountable governments from the North, from the colonisation, and and it's not. And I think that the narrative out/ outside, I mean, we talk about those kind of things in ehm in some rooms and in some, yeah, some rooms like the UN halls and these kind of things. But then in the news and in the president's speeches, et cetera, there's a lot of imperialism like going through their speeches still and through their practices. And I I agree completely with what SC said in ter/ in terms of of how the/ how are we keeping accountable corporations? Because those are the ones that are ruling the the world right now. And how are we talking about that? They have more power than most of the/ our governments. So how do we create a system that is also (...) creating some kind of, I don't want to say deep, because I don't think that's like, how are we not, (...) (gestures with her hands) I don't I, (sighs) I just want to burn it all. (SC laughs) I'm sorry I'm not being fruitful. (laughs) I just see the problems. I don't see any solutions. (laughs) Let's burn it all and start over. Let's see if that works. (laughs)

[1:16:18.5] **HE:** Thank you, yeah. (laughs)

[1:16:21.4] **OLU:** I woke up very pessimistic, sorry. (laughs)

[1:16:23.7] **HE:** Yeah, but maybe you have ideas how to //

[1:16:27.2] **// SM:** Hi HE, sorry, I couldn't answer the question earlier.

[1:16:29.7] **HE:** Yeah, yeah, no //

[1:16:31.1] **// SM:** I was just busy

SM: But can you just repeat the question?

[1:16:33.7] **HE:** Yeah, we were just ehm, I was just wondering ehm, because you, of course you mentioned how important context is, and your local adaptions, and from the people who are actually actually part of the community, so I was wondering if it's even possible, or or something to to pursue if we have a global and a very holistic framework, for example on feminist global health policy? And then I was wondering, is this actually something we want? And what could this very global holistic framework encompass? And of course, if we have to think that it should be adapted context specifically.

[1:17:15.0] **SM:** Ehm yeah. (laughs) So that's a big question. And ehm I I I think you're moving us into

a sense of imagination. Ehm I don't know that we can imagine outside of a Western frame. Or outside of the current system, when it is actually so entrenched ehm in how we do things, how we talk, even the very fact that we're having this conversation in English, yet we are from three different continents and the traditions in each of these continents, context-specific, ehm are so vastly different. Ehm I think that we ehm it it makes it the task of re-imagining really difficult. Ehm and yet it is possible, I think that we have to keep imagining, we have to have that hope, somehow, because I think that if we stay with a sense of the despair it's not going to really help us ehm move forward, and it's not also going to help us to kind of imagine alternatives. So I think the first thing we've got to do in that imagination again is that we must acknowledge that all of us are deeply colonised. And all of us are deeply operating from a space of ehm really ehm, I guess ehm ehm that space of of trying to to to perform a particular type of ehm/ we're trying to fit in, we're trying to advance our own personal careers and personal lives, while at the same time ehm doing the work of ehm social justice or or/ And and I suppose it's what I'm asking, what is feminist policy for, what's the goal? And if the goal is social justice than we all trying to do that within the the very difficult complexity of survival. So I just want to like step back and acknowledge that every single one of us has to find a way to materially survive on a personal level while doing the work of the imagining, it's the work of imagining is sometimes to actually say, do we need to break everything down and start from scratch. Or do we need to ehm build from all the ash, or do we need to try and turn things around. Ehm so I think yes, that's the real complexity around imagining. And I think that the imagining project is/ ehm people are imagining different ways and it's about who we listen to. And I think, I caught a little bit of ehm SC saying that perhaps we need to turn to where communities are ehm communities ehm (...) where whe/ where is the real structural silence that they experience and where they're trying to exist. Because these are communities that are are, that face some/ the the deep (grant?) of the layer of intersectional approaches. And they're surviving. So the the survival ehm and the ability to resist is something that we need to get to know. And we need to start listening. So we need to stop looking to people in power and who currently hold power and don't have the lived experience of the systems of oppression to provide solutions. Because they actually don't know. And it's not (their fault if they don't know, they're in?) a position of privilege to the point that they cannot submit to the experiences of those ehm

of ehm im/im/ impoverished situation.

[1:21:03.5] Yet those who are in those positions of of poverty and inequality, they actually know. And so we've got to/ we've got to shift who we listen to. Because that's where the imaginist of work is actually taking place because they are grappling on a daily basis with the issues. And so (...) and why I go back to colonial issues and the, where I started with the elements of Othering is that, the the problem with the colonial project, it's convinced us that those of the people who are poor are non-human. So they don't have an opinion, but we cannot spend our time listening to them because they don't know. But actually they do now. They are the experts (...) of their own lives, and they are experts of the problems of the system. And so we've got to turn to them to demonstrate ehm to to show us ehm alternative and possible ways. I think that we also can turn to communities ehm like like the Maori community. Ehm and perhaps people/ some some Latin American communities, like the Zapatista movement, and I I'm not really ehm suggest/ I don't know enough about them. But I I I think maybe even the doulas that were mentioned earlier. Because these are women, who are largely women, ehm who are reclaiming their ehm their humanity. They're reclaiming their rights to (...) to exist. Ehm in a way that they wanted to exist. They don't want to be working medical industrial model, they want to serve ehm with a community around it, with a system of care. Ehm with indigenous knowledge. Ehm, they don't want the the medical gynaecologists to be the one that's the only one in charge of birthing. They are actually restoring the idea that every woman has this natural knowledge of how to birth. So, you know, I I'm I think that we've got a lucky ma/ the imagining project, how do we do things differently. Ehm, do you d/yo/ it depends on whether you can only imagine in the current frame that you're existing in. Or can you widen that frame and ehm and and and if you can widen that frame and you can think outside of the current system, what are you thinking about? You know, ehm, so I'm I was interested in your topic because you're asking the question of imagining. And I'm interested in that question and I'm interested in knowing ehm ehm how we can imagine. But I do know that we also have to think about where you are located and as a researcher as well. That is trying to think about imagining ehm and how those of us in a (form?) are located and where we are thinking about imagining from. Because each of our positionalities also either provide us with an opportunity or an limitation to be able to imagine outside of ehm a system, you know. And some of the women I work with in in are are women who are migrant women, who experienced layers of li/ like

ehm violence across their life course and (this is?) gendered violence, state violence and and so on. And so they are the women that I'm really like trying to turn to. To to to help ehm uncover some of these ehm answers. Ehm I don't know, I'm I'm hoping I'm not complicating it, but (laughs) that is my thinking, right now. And I hope it's helpful. Thanks.

[1:24:39.4] **HE:** Thank you very much for sharing your ideas. Ehm I don't know if the others want to add on it because you mentioned so many aspects. (5)

[1:24:53.7] **SC:** I'm sorry I just have to leave now. Ehm I/ thank you very much ehm I have my mom has/ is waiting for a dinner, I have to give her dinner, so I'm sorry.//

[1:25:07.8] **// HE:** No problem. I'm //

SC: Thank you very much, and I hope we will stay in touch somehow. Ehm thank you, HE, for bringing us together. And ehm, thank you, SM and OLU for share this sharing, learning, experience. It's been really really a lovely conversation. Thank you.

[1:25:23.5] **HE:** Yeah, thank you for participating. (smiles) Ehm maybe one second ehm because I had the other focus group yesterday, and they asked if ehm I can also give them this recording. And then you would also get their recording because maybe you're all interested in what the other group said. (OLU nods and agrees)

[1:25:41.1] **SC:** Sure.

HE: Okay, great, great. Okay. Yeah, then have a good evening and ehm thank you so much for participating.

[1:25:48.9] **SC:** Thank you. Bye bye.

[1:25:50.8] **HE:** Bye.

[1:25:54.2] **OLU:** I was just going to say that, I (...) I mean yeah. There were all those things (laughs) that SM said ehm sh/ she/ SM you're very articulated, thank you. Ehm (...) There's still the thing about gender and decolonisation and I think that it has to do also with education. Like I don't see how those things can be can be separated. I think that the way that we see health has to with with the way that we have been educated. And what kind of, and, as I was saying like for Mexico in particular, there's a lot of violence, and I think that there's, there's still a part of education that is not taking into account how we manage emotions, even. (smiles) Like I think that working around gender and and to be able to to to (...) to change the whole culture around gender. It needs education, and it needs mass media to be involved in it. It needs to be, I mean, how are

we all together trying to change that. I think that that's that's a tricky question, because people/ I do think that gender is something that's (natural?). And some feminist (laughs) groups are also like (...) furthering that more and ehm and essentialising gender as something that is intrinsic to to women, you know. And like there's these differences that just make us who we are, as if women was not also a creation that we (...) human kind did at some point in history. And I think that there's there's a lot of things that need to be tackled in terms of how do we/ because that is that has to do with the way that we share or not share power. And that has to do with the with the way that we hiera/ hierarchise, I don't know (unint.), but how do we build on hierarchy every system that we think of. Even in feminist collectives. Sometimes they have hierarchy, you know like, that, and I know that, some of them don't, and I think that, I think feminism and anarchy are the one / the the two (laughs) movements that can can to / can play more with that kind of a structure. But the rest of the things are very structured in that way of of the power being centralised, and the power being, you know, like not shared. I don't know, I don't think that we, we are able to, I think that we need to change the way that we share power, and that has to do also with/ the distribution of power has to do with redistribution of money and resources in general. And if we don't do that, I don't think that we're going to be able to really have proper access to to quality health. And we keep just ehm turning down fires. Like with Covid, you know, like it's just like, how do we put the fire out? (underlines her speech with gesture) We're not changing the structures that are that are that put us in that position. And just to all these inequalities that were before that with every (forward?) that would just like further deepen with ehm with Covid. And we're not doing anything about that. It's just like, okay, vaccines that's it. (claps her hands to underline her speech), move on. But nobody is taking care of the inequalities that we saw how deeply affect when when there's an emergency and when there's this kind of crisis. It just, it's just leaving the weakest behind all the time. Even if we have this narrative of not leaving no one behind. We are leaving everybody behind all the time.

[1:29:11.2] **HE:** (...) Yes, thank you. Ehm and also ehm I like that you also mentioned, because we were talking a lot about structure and political power, financing, but I think it's also a lot about society and so/ social change. (OLU nods) So how do, do you think ehm we can get to the people by and and also and yeah by showing them that ehm if we think about a feminist global health policy, but maybe more in general, that another approach would

maybe be more beneficial for everyone. How how can you get this message across? Ehm, yeah. Maybe you have some answers to this? (laughs) (8)

[1:30:00.3] **OLU:** I had like three thousand notifications coming at the same time in my ear. So I would have to to rep/ to ask you to repeat the question again.

[1:30:08.4] **HE:** (laughs) Yeah, I was just ehm asking that, how do we (...) get the members of society to to, maybe to understand or to transfer the message that another approach would be even more beneficial for for everyone, actually. So. ehm. If we more, rather, (SM unmutes herself), yeah?

[1:30:28.1] **OLU:** I think /

[1:30:30.8] **SM:** I can (come in here?). Ehm, just, it's (unint.) time, sorry. (laughs) (unint.). Ok, let me go over there. Yeah, I I I don't know that people are going to really give away their power. (OLU nods) That's the scary thing. If they were going to go and give away power, distribute power, share power, we were / then we would not be an inequitable world. Ehm, I think that we have to start facing how much we love power. Those of us who have power and the power that we want, we want more of it. It's an insatiable world ehm we live in. People are not satisfied. They always want more. Ehm that ehm, you know, that's the capitalist strategy, promoting the element of greed and extraction, extraction of ideas, extraction of knowledge, extraction of (power?) shifting of the resources ehm I think ehm distributing only so that it serves the capitalist project. So I I think that, you know, the project of resistance (hasn't been a fight?). So it's not really going to be easy to tell people that we need to make a change. Ehm I think that we have to start trying to ehm move that process of imagination, that you brought us to earlier, to understand what does reclamation look like? Ehm, what does it look like for me? You know, what does it look like for you? Because ultimately ehm the colonial project dehumanises us all. We're all in the system (laughs), work in the system. And until we all kind of reckon with that and how we (pass of?) it, ehm we can/ It's not it's it/ So I'm saying it's not anybody else’s work. The work starts at the individual level. Ehm and we are able to to to make that change ehm, you know, and if you have a critical mass of global health scholars/ not even global health means, because I also don't, I don't (unint.) I don't understand that term, it doesn't really mean much to me. Ehm but it's many of us who are concerned about the issues of equity and health are able to (join?) the critical mass of shifting how we do things, and to be confident enough to to do that, and to reject certain ground or to try to demand

ethical ehm money or to look at money as maybe reparations rather than ehm, you know, donor aid. And it's this that holding people accountable because ultimately (unint. come down to resources?). (OLU nods) Ehm and if we if if if it's a model of how resources are a a/ are distributed ehm ehm and engaged to us in a different way. I think we're going to start ehm making a change, you know. So I think unfortunately it's nothing to be in close (reach?) it's going to be a very uncomfortable, difficult, hard process. (OLU nods) And it's going to require work and resistance. I mean, are we here for a revolution? I think that's the question and I suppose the question of decolonial thinking is that it's asking for a revolution and I don't know that we know what revolution looks like, you know. (OLU nods) And people get scared when you talk about revolution (laughs). But, you know, until until we (...) the current situation is (...) that so many people are dying, and their lives are are ehm/ and we have knowledge of how to prevent and save their lives. And so many children are not having enough food so they can't grow ehm/ they/ they they're starting life in ehm affected and so I don't understand why we're not more angry and not doing more (laughs), (OLU nods) you know. I just/ I don't understand why we're not angry enough to actually get out into the streets and to stop this (laughs). And and I'm saying this to you standing in the Eastern Cape and I'm working with people who are sharing the realities on the ground of of the si/ the situation. Ehm and I think we've turned a blind eye. So, until we're willing to be brave enough, to admit that we make mistakes ehm we don't know ehm we need to do things differently ehm and and kind of learn to listen. I think that's all I can tell, I don't have anything more to say, ehm except that we need to go out and do those things. (laughs) Yeah, thanks.

[1:35:38.1] **HE:** Thank you very much. Yeah, very, very many important aspects, and ehm I think all of them really true. (...) Ehm, yeah.

[1:35:49.0] **OLU:** I (coughs) I think there's already some of the victories of of the work that we have been doing for for many years. We, as a feminist movement. Like I think that young people are already demanding some of the respect regard/ regarding intersex, regarding non-binary, regarding trans people. And I think that that is the thing that, I mean there's a change that is happening. But it's going to be, as SM said, a long term, it's not going to be something that we can (...) I don't know, be able to hold and and say "Yeah, we did it". (smiles) But ehm but for sure, I think that it is happening, and I think that we need to to to be able to/ I mean, we have agreements at the international level, and we just

need to keep pushing for those to be implemented. I think the Beijing Platform of Action, this was very specific on all the cultural change that we wanted to happen. And I think that it's still relevant, and we we should/ I mean, there's there's this political, supposedly there's a political will happening from some ehm countries and these action coalitions and this kind of like organisation around, like keeping that platform alive. And I think, I mean I'm being very institutional, but I think that that's a way that we can push for governments to to to be on that track. Ehm and it's, I mean that/ the idea of like being that the feminist is being trendy, and that we're wearing a t-shirt and wearing it in, you know, different (laughs) merch and this kind of thing. And even, I mean it ca/ it can sound like ehm capitalism in co-opting this (laughs) the movement, but it's also about like how how that narrative is changing. And we're not being demonised and I think that is, I mean even if it's a little bit of pinkwashing, it's still valuable to see that there's people that are actually starting to see it from that perspective. Ehm, but I am still up for burning it all and (laughs) I think that these systems of oppression that are working right now need to like a radical change, you know. Like being able to see how capitalism gets in our heads, and it's colonising our brains and, you know, like sometimes in the mornings, when I'm not able to wake up, I know that my capitalist self is going to be like "You're not being effective", you, you know. Like even like religious, you know, like feeling guilt, I mean all these kind of things is how (...) how the system has has become part of us, and I think that it is important to talk about these things and being more holistic in a way that we are fighting against the systems of oppression. And I'm figuring out ways that we can (...) still have some reflections and questions out there and just being able to cri/ critique. And and even if we're not offering solutions, but asking questions and and ehm being able to to have deeper reflections on how are we doing things and trying things and experiment things. And I think that that is/ that is as good as (...) as we can do. So. Ehm (...) your question exactly was? Because I think that I just went on on a real rant. But ehm. (laughs)

[1:38:57.6] **HE:** Ehm (laughs). Ehm my question before was (...) also to, how to get to society and or to members of society to, to //

[1:39:06.0] // **OLU:** Yeah. I think

[1:39:07.5] **OLU:** I think we're getting there. I think the/ we're not the only people that we've seen now how the systems of oppressions are working. But definitely we need to keep able to to

to to to to stay (...) as reflecti/ as reflective as we can, and to invite others. I think that sometimes feminists are only talking to fem/ to other feminists, and I think that we need to improve the way that we communicate our messages better to other people. Because we o/ oftentimes we're just speaking these big words like patriarchy and (laughs) capitalism, and neoliberalism and these kind of things. And that is not going to relate to my aunt, and it's not going to relate to to my neighbour. So other strategies that we need to do also in the ways that we communicate from a value (uses air quotes), you know, also, but what we've talked about these principles that are the framework for governments. I think that we also need to find the values that are in the framework, and that that can reach out to other hearts and minds outside the movements. (...) To join the movements. (laughs)

[1:40:08.4] **HE:** Yeah, that's really true. Thank you. Ehm (...) Yes, SM.

[1:40:21.8] **SM:** Sorry, I'm I'm going to have to leave soon as well because ehm we just yeah. Anyways, I I wanted you to add that I I certainly believe, and I also think that ehm it has been really ehm interesting to see how suddenly the word decolonial thinking and and ehm is a buzz word. You know, there has been (many things?) like planetary health is this huge buzz word and people are now like really trying to locate themselves as decolonial thinkers and scholars and feminists and so on. It's it's a new trip. And I think it's very/ We have to be very careful because then feminism became something that entered the academic space, and not, it didn't live in the homes and in the, you know, in a in a (unint.) activist groups and in communities ehm and and (unint.) resistance movement. And it started becoming something that people studied and anybody can take up feminist studies ehm. It kind of lost (laughs) it lost the radical nature of it. And I see that decolonial thinking is yet another term that is going to do the same and yet it's also going to be isolating and alienating to to some. Because it's it's difficult, it's a new discourse and it's not easy to always ehm break it down.

[1:41:43.3] So I think that we need to be careful about what claims we make. Ehm and to start defining more carefully what we are trying to achieve and what we are trying to do. And I think we, we as those of us who are really ehm (...) ehm resisting on the ground ehm (unint.), we also need to start paying attention to other forms of power that are going to intentionally trying to undermine what we're trying to do. Ehm and and to and to just be able to ehm yeah, I think be quite careful. Because I think in every society there's

always room for some sort of ehm openness for for alternative views. But (...) ultimately the status quo is remaining. And it is very clear, during Covid-19, that vaccine access was ehm/ as soon as the vaccine access and through the realities of how many People of Colours across the world were disproportionately dying (...) that ehm (...) we aren't in, we are in a crisis. A global crisis of ehm values. And of this issue of care. Ehm and I think that the the for me this the the core of this (topic?) is that feminists and feminist thinkers, valuable feminist thinkers, and for my space it's, you know, it's who do we/ who do we refer to them, you know. For me it's people like (unint., names of South African writers and thinkers) from from who who's in a community that I work with. Or ehm, you know, sometimes I refer to Audre Lorde as well. But she's not from my home, she's she's an American. So I think, you know, feminist thinkers have really called us to settle in our homes with our mothers, with our grandmothers, and listen. And to build that community over food and this element of radical love and care. And I think I'm (unint.) the right values due to ehm global policy making, which I think is like a probably (laughs) not yet an easy thing to even conceptualise, probably a joke if you write if you (probably?) hearing what I'm saying. Ehm but I think that way, what what needs to happen. Like it it gets, it's actually, you've got to be careful, and we've got to be aware of who's doing work that feels like it's aligning to the values of feminist work and those that are talking the talk, but in the embodied action ehm, you know, they aren't. Ehm I'm very, like I'm part of academic spaces, but I'm also quite critical of academic spaces because oftentimes I feel like academics can can can theorise but the work that will translate to an embodied practice of ehm li/liberation and reclamation. And this is why I say we're going to turn to those who are doing that embodied work and who can teach us how to read this, or how to speak in ways that people can understand. Yeah, thanks. Ehm and (unint.) that, I'm going to leave. (laughs) Take care.

[1:45:10.3] **HE:** Thank you, (OLU smiles) thank you so much for joining also, SM. Ehm I think we can also end the session then ehm. I mean OLU if you want you can also (smiles) add some more aspects but I also don't want to keep you any longer (laughs) if everyone else is leaving. (OLU smiles) Ehm yes, thank you both very much for participating. Ehm (...) I will send you also the video, (OLU nods) and also the transcript and everything, and from the other group, it will probably take some time also. And then, of course, also the results. Ehm if you ha/ want to give feedback or anything ehm. So I will definitely come

back to you. Ehm, yeah. And also I don't know if you want to stay in touch with the others, but your, your e-mail addresses I sometimes send emails to all of you. So/

[1:45:54.6] **OLU**: Yes, thank you. (smiles)

[1:45:55.7] **HE:** I will probably contact you.

[1:45:56.7] **OLU:** Yeah, thank you. Thanks, thanks a lot, HE. I hope that you had everything that you needed.

[1:46:02.7] **HE:** Yeah, no, it was really interesting for me definitely. Yes, thank you so much, and have a good day. (smiles)

[1:46:09.3] **OLU:** Ok. You too, bye, bye. (smiles)

[1:46:11.1] **HE:** Bye! (smiles)

Transcript of FG3

**Date of the focus group:**	7 September 2022, 18:00 CET
**Duration:**	98min 22sec
**Place:**	Online via the audio-visual tool Zoom
**Moderator:**	**HE**
**Participants:**	**AN, LDA,** (NK could not attend)

Transcription notation

**(…)**	Break up to 3 seconds
**(number)**	Break longer than 3 seconds, duration in brackets
**underlined**	Particular emphasis
**(nonverbal)**	Nonverbal expression
**(unint.)**	Unintelligible speech
**(word?)**	Unintelligible, assumed speech
**ehm**	Uniform notation of filler words (ehm, ah, eh)
**/**	Interruption of word or sentence
**//**	Speech overlaps

Transcript

1	[0:00:00.0]
2	[0:06:30.4] **HE:** Hello, hi everyone. (9)
3	[0:06:40.8] (AN waives and smiles. Unintelligible sounds from LDAs microphone.)
4	[0:06:52.8] **LDA:** Hi!
5	[0:06:54.6] **HE**: Hi, hi LDA. (smiles)
6	[0:06:56.9] **LDA**: How are you?
7	[0:06:59.0] **HE:** I'm fine. Thank you. How are you?
8	[0:07:01.7] **LDA:** I'm okay.
9	[0:07:03.5] **HE:** Good. (smiles) I'm glad it's working this time.
10	[0:07:06.6] **LDA:** Yeah. (6)
11	[0:07:15.7] **HE:** We're just waiting for NK. (AN nods) (...) But I think she will join us soon. (5)
12	[0:07:26.1] **LDA:** Okay, no problem.
13	[0:07:36.8] **AN**: (8) I think your video is having a little, (gestures with her hands), it's a little

disruptive for me, and I'm worried that it's my internet connection. But it should be great. (laughs)

[0:07:48.9] **HE:** My video? Okay.

[0:07:51.5] **AN:** Ehm can you see me all the time, even if I move?

[0:07:55.0] **HE:** Yes, yes, I see everything.

[0:07:57.2] **AN:** Ehm because I don't see yo/ I do see you but I only see you frag/ fragmented, unfortunately.

[0:08:04.3] **HE**: Okay.

[0:08:05.0] **AN:** Mhm.

[0:08:06.2] **HE:** LDA, do you see me clearly?

[0:08:08.9] **LDA:** Yeah, I see all of you very well. (laughs) You want to use a video?

[0:08:15.8] **HE:** Yeah, if you don't mind. (smiles)

[0:08:19.0] **LDA:** Ehm for a short while, I think I can try.

[0:08:22.6] **HE:** Okay. (LDA turns on her video) Great, hi. (smiles) (AN waives and smiles)

[0:08:26.3] **LDA:** Hi (smiles) (...) So you see me now.

[0:08:30.3] **HE:** Yes, yes, I //

// **AN:** Yes, I you. I see you wonderfully, too.

[0:08:34.3] **AN**: For some reason, mhm.

[0:08:36.4] **HE:** But do you think it's my connection? (AN signals that she is unsure)

[0:08:41.0] **AN:** No. (shakes her head) //

// **LDA:** I see all of you.

[0:08:43.4] **AN:** I see all of you as well ehm (...) No, but I ehm. (AN turns off her video) (13) (AN turns her video back on)

[0:09:06.9] **AN:** Ehm we can just try it, and if it doesn't work I just turn my video off in the meantime.

[0:09:11.6] **HE:** Okay because if it's my connection, I can also try something else with the internet, you can just tell me. (...) But I see you very clearly, so, maybe it works. (AN smiles) (5) Yeah and thank you also, AN, for adapting ehm because I know you had so much going on, and I was writing a lot of e-mails, I know. (laughs) (AN and LDA smile) But I'm really happy that you're here now.

[0:09:40.8] **AN:** Thank you for inviting me and I'm very happy to be here as well. (HE smiles). It was not too many emails. (smiles)

[0:09:47.8] **HE:** Okay, thank you. (laughs) (57)

[0:10:48.8] **HE:** I'm just checking, maybe NK sent me an email. (AN nods) (88)

[0:12:23.0] **HE:** She didn't, but I think she will come. Ehm maybe I wait two more minutes, and then ehm I have a little introduction, so I will just start with it. Sorry for keeping you waiting. (AN shakes her head) (74)

[0:13:44.2] **HE:** Okay, I think I'll just start, ehm and she'll probably just join a couple of minutes later. Ehm, but you can see and hear me clearly now?

[0:13:58.1] **AN:** See yeah/ ehm hear, yes. See not so much. I'm going to just try turning my video off too and then we'll see if its//

// **HE:** Yeah, I can also try something.

[0:14:16.4] **HE:** Yeah, maybe it works better this way. (...) So (...) Yes, so ehm welcome and hello and thank you so much for joining. (AN smiles) I'm really really glad and also curious that we're having this discussion today, and I'm really happy that I can do a third focus group. Ehm, and also because I know that you're all very busy and you have a tight schedule. I'm really thankful that you are here today. Ehm, before we start, I just want to quickly start with some housekeeping. Ehm, as you noticed, I already started the Zoom recording. It's just so that I don't miss anything. And it was also easier to set it up this way. Ehm and I also sent you the formalities from my university about the procedure, about data policy in advance (AN nods), so th/ there you can find all the information, but ehm you all sent the document back to me so I assume you know about this. But if you have any objections you can also tell me now. (...) (AN shakes her head) Great ehm, yeah. Then for the discussion, if possible, I invite you all to keep your video or turn your video on ehm. I think it's just nice if we see who we're talking to. But of course, if you don't have a stable internet connection ehm, or there's something in the background then it's no problem. Ehm, I mean the most important thing is that we hear you. And also ehm I want to ask you to try not to talk too much at the same time, also because I have to transcribe the session. Ehm, but of course this is a free discussion. So whoever wants to talk can just do so. Ehm, so you can just jump right in ehm I don't have to pick you to talk. It should rather be a natural conversation. (AN nods) Ehm but we are two or three people so I'm sure it will work this way. Ehm, yeah, and that's actually all about the technical stuff, ehm and then I should also introduce myself properly, I think, because I only had e-mail contact ehm to all of you before.

So, as you all know, my name is HE. My pronouns are she and her and I'm 25 years/ 25 years olds. Ehm I live in Berlin, in Germany, ehm and I study the masters Public Health and Political Science at the University of Bielefeld. And I study these two masters because I'm in particular interested in the intersection which is global health policy. I also worked with some NGOs here in Germany in the field of global health. And right now, I'm part of a university project on decolonisation of global health. And more personally, I ehm, I like doing sports in particular dancing (AN smiles) or going for run, and I also enjoy very much living in Berlin having this, this offer of everything around me, cafes, restaurants, yeah, So, that's it about me. Ehm then maybe I would suggest that ehm you also introduce yourself ehm so that we get to know each other and also it's a bit more interaction and it's not me talking all the time. Ehm yeah, so maybe AN you want to start and tell us something about yourself?

[0:17:35.2] **AN:** (smiles) Sure, can you hear me well? (HE nods) Perfect ehm well thank you for your introduction, and also for ehm, telling us a little bit about you and it's very nice meeting you (laughs). And ehm yes, I'm AN, my pronouns are she/her as well. I am a medical doctor. I studied in Berlin as well at the Charité. Ehm and I'm also, right now, currently a medical researcher at the University of Bielefeld, ehm specialising or working in sex and gender sensitive ehm medicine. In particular, looking at European guidelines and ehm analysing the current state of sex and gender sensitive medicine in the European guidelines. And ehm 2020 I was lucky enough to find ehm, like-minded people to co-found ehm the NGO Feminist Medicine. Ehm we are currently in Germany and in Austria. Ehm and we, as a foundation we have intersectional feminism, and we promote gender equality, and also obviously ehm an intersectional approach ehm all marginalised ehm people in society in the German-speaking societies yet. (laughs) And (...) that's actually about it, I think, what's what's interesting and what what makes sense for now. So nice meeting you and nice being here. (smiles)

[0:19:08.6] **HE:** Yes, thank you very much. I'm also really happy to meet you. (smiles) Yeah, LDA. You want to introduce yourself?

[0:19:15.9] **HE:** (8) We we can't hear you. You're muted. Sorry. (smiles)

[0:19:31.3] **LDA:** (...) Sorry. (laughs)

[0:19:33.0] **HE:** Don't worry.

[0:19:36.0] **LDA:** Okay. (...) It's already a good evening in our country, so good evening, good

morning to everyone. (Everyone smiles) I'm LDA. I I am from Uganda. I am ehm a queer woman and a sex worker. Ehm my pronouns are they/them. Ehm I work with an organization called OGERA Uganda, Organization for Gender Empowerment and Rights Advocacy Uganda, as the Advocacy and Networking Officer and ehm we work basically to improve ehm the quality of life for female sex workers and urban refugees who identify as LBQ. And ehm, our work is around research and documentation, advocacy, ehm we also work towards economic empowerment ehm and health plus legal services. So we are very happy to be here, and LDA is a public health specialist, an influential civic ehm ehm speaker. Ehm on different platforms. And ehm, I represent a lot of ehm a lot of young people identify as key populations in the country and different platforms. I sit on the African Queer Youth initiative, where I do advocacy on a regional level to make sure that young people who are into sex work are recognised, and their rights are protected and promoted. So I'm very happy to be here as a person, but also as an organization because I know this ehm as we're are talking about global health policies, I know we need to voice out ehm voices for the vulnerable communities where I represent. So I'm really happy to see everyone. And finally, HE, I see you now. (laughs) So yeah back to you. (HE laughs, AN smiles)

[0:21:17.7] **HE:** Yes, thank you so much. Ehm I'm also really really happy that you're here, ehm and that we managed to do this focus group (LDA smiles). I also hope that NK will join us. (AN nods) Ehm, but yeah. She had a training until five, but she actually said she will manage, so I'm still ehm optimistic that she will join, ehm I can just say that she's the Program Coordinator (AN nods) of the Global Unit for Feminism and Democracy at the Heinrich-Böll Stiftung, ehm based in Sarajevo, in Bosnia and Herzegovina and she's also eh a feminist activist for many, many years now. Ehm, maybe she can also tell us something about her work if she joins. Ehm I also prepared a very short power-point presentation just to remind us why we're here, what's the topic, what's the expectation? So I will share my screen with you. (HE shares her screen and opens the power-point presentation) But it's also basically everything that was ehm already in the Concept Note. Ehm, yeah. So, as you know, this whole research is part of my master thesis with the topic Feminist global health policy – addressing health inequalities through an intersectional perspective. (HE changes to the next slide) And this project is based on an intersectional approach, and it focuses on the structural determinants of health, gender, race, and class. So this is based on the WHO framework on the social determinants of health. And this framework

emphasises the influence of the political context, and also the role of power regimes. And in my thesis I also give examples of the impacts this structural discrimination and also the inequalities have on health and well-being. And I give examples of the areas, you see listed here. And I focus a lot on gender inequality, but I also consider racism and classism throughout in all these examples. (HE changes to the next slide) And here I brought you my understanding of a feminist global health policy. So this is based on the literature. Ehm this means that maybe we end up with a new definition today, or maybe you have a different understanding. Ehm then, of course, you can tell us in the discussion, but we can maybe just use it as a starting point. So for me, a feminist global health policy is a very holistic and intersectional approach. This also means it should be inherently decolonial, and it recognises the influence and the importance of the socioeconomic-political level. And it wants to challenge, and also to change power hierarchies and the resulting structural discrimination. It does so by focusing on the most marginalised first, and by shifting to more participation and anti-discrimination. So in my thesis ehm I aim to create a framework on feminist global health policy, and also to give specific recommendations how it can be successfully implemented, as far as this is possible. And I use the focus groups to to gather my results. (HE changes to the next slide) So for the discussion, I want you to keep in mind that the underlying principles are intersectionality, and also the emphasis on power regimes and I have this, this very broad structure of ehm What, Who, and How regarding a feminist global health policy. So just to have this, yeah very broad frame in mind for the discussion, and I will stop here. (HE stops the presentation) (...) And ehm then there's one more aspect I would like to add, which is important to me. Ehm because you know that I'm adapting feminist research methods and also I use the focus groups so that I say rather in the background. And I hope that you can use the results for your own purposes, and you also benefit from the interaction. But I'm also ANre that this is not a perfect project. Ehm, and also, I am a white woman, I come from a Global North institution. So I'm a very privileged person, and also my position also influences the research. Ehm, so I try to be very reflexive about it, and to include this ehm in my thesis. But I just want to say I know that there will probably remain some power hierarchies also because these discussions primarily serve my master thesis. Ehm yes, so ehm that's everything I wanted to say before we start the discussion. (smiles) Ehm, so for the discussion I prepared some guiding questions, ehm but

as I said, the focus is on you, so it can also take a different path. As long as we keep in mind a bit the aspect of What, Who and How ehm regarding a feminist global health policy, and also how it can be implemented. Ehm, then I also want to say that this is an open and non-judgmental discussion. So there are no right or wrong answers. Also I don't intend to find final answers. Ehm so maybe we also end with a couple of new questions, so this is also fine. And yeah, and of course you can also disagree with one another and ehm discuss a lot. So ideally, I did most of my talking by now. Ehm, I think we just start with the two of you. Ehm, I don't think that's a problem, I think you have a lot to say. Ehm, I also sent you the first question in advance because I want this question to be answered by everyone, and then we can start with a more open discussion. So ehm for the beginning, I would like to know what do you consider the most pressing structural challenges regarding global health policy at the moment? And ehm, I don't know if someone wants to start. Otherwise, I can also pick someone. (smiles) (7) So maybe, LDA, you want to go first?

[0:27:26.0] **LDA:** (9) I I beg your pardon on the question?

[0:27:32.2] **HE:** Don't worry. Ehm I asked, what do you consider the most pressing structural challenges we have in ehm global health or global health policy at the moment?

[0:27:45.3] **LDA:** Ehm, I think when we look at ehm at the un/ Universal Health Coverage where we have to make sure that ehm services to everyone, regardless of their ehm gender, of their origin, of their where they come from, and everything. We need to focus more on the accessibility, affordability, ehm equitability, and the availability of the services. It is very, very unfair for us to have services that ehm, I'll speak in regards to my community, where my community cannot afford the services many of the times in my country. So we need to make sure that the global health policy at least ehm speaks to that towards that has a powerful speech towards making services accessible, affordable, equitable, applicable. And they need to be centred that everyone, regardless where they come from, they can access them. Yeah.

[0:28:49.6] **AN:** Ehm, one question in advance, LDA, can we say LDA and AN to each other? Or do you want me to ehm call you out on your last name, or what's what's your favourite? Just so I know how to ehm address you, if I say, like LDA, you just said this, I agree on this. What do you prefer?

[0:29:11.5] **LDA:** (unint. due to bad internet connection)

[0:29:17.0] **AN:** HE?

[0:29:18.5] **HE:** Ehm yeah, we can hardly hear you, LDA. I think your connection just got lost. (...)

[0:29:25.1] **AN:** Yeah maybe the no camera option is, yeah, better.

[0:29:29.3] **HE:** Yes (...) So but, LDA, could you hear ehm the question? So I think actually, you can refer to each other by first names because ehm that's what you also signalled to me. But yes, maybe we'll let LDA speak.

[0:29:48.2] **LDA:** Sorry?

[0:29:49.6] **HE**: Yeah, AN asked if it's okay if she ask/ ehm if she calls you by your first name.

LDA: Yeah, she can, she can feel free to call me any of my names, it's very okay with me. Yeah.

[0:30:01.5] **AN:** Perfect, so same for you too, LDA. Nice to meet you. Ehm I want to ehm link my answer to what LDA ehm what you just said actually because ehm for me, I think four main points are essential. If I look at at the most pressing problems, or most ehm relevant ones. I guess it was very difficult for me to to focus it, but I think four main points are essential. Ehm one of it you've you've already said LDA, is the accessibility. Ehm, I do think that ehm, a global health policy needs to be accessible for everyone in society, and therefore it needs to be transferred from like an elite discussion to a discussion that includes civil society, and that ehm is also shaped by civil society. Ehm the second one, I think, ehm is also linked to what you just said, LDA, that I am, that I believe that we/ whenever there is a framework, it also needs to, we need to keep in mind that it needs to be contextualised somewhere, like it's never, it cannot ever be just one framework. It needs to be ehm always thought in context of the local and resources, and whatever people need and ehm whatever place they are. And then ehm the third point, I think, is involving everyone. It's linked to the first one already ehm that I do also think that including all genders and all backgrounds in terms of an intersectional approach is absolutely essential. Ehm and that that needs to be that we need to mirror society also in the policy-making processes, that it cannot be, ehm like it is right now, that the that the policymakers ehm, may I say just to just say like that, it's like mostly cis, white, male, ehm and it's mostly able-bodied and ehm the Global No/ North who's shaping these frameworks, and ehm that is something that needs to be overcome. And therefore what you've already said, HE, ehm (...) to rethink power hierarchies, and to reflect them in every step of the process. It's essential, just as a ehm, I think, from a meta- ehm viewpoint, I guess.

[0:32:36.8] **HE:** Yes, thank you very much. Ehm, I think we can touch on a lot of the aspects ehm, both of you mentioned, ehm, during the discussion. So I want, also would like now to shift a bit from the problem-focus to a more ehm solution-focus and thinking about yeah, what alternatives a feminist global health policy can provide. You already touched a bit on that AN. Ehm and also, yeah, what key compo/ components ehm would would it have to consider? And also ehm because you said it has to be context-based. Maybe nevertheless, there are some like universal principles or something we can adapt at a global universal level. So maybe you ehm have some thoughts about that. (6) So I would just say, whoever wants to start can do so because I don't see both of you, so just feel free.

[0:33:43.2] **LDA:** Okay, I I will start. Ehm we think about ehm solutions or alternatives to make sure that, ehm, the global health policy contributes to ehm improving the lives ah, the quality of life for women. I think, when we talk about diversity and intersectionality, it should be at the forefront of that document. Because this is a framework that has to structure, you know, human rights in different approaches to make sure that ehm people are re/ people's rights are responding to, and everything. And then some other thing I'm thinking about is ehm, we need to also make sure that women human rights defenders are also not left out in that framework. Because these do a very, very big role to make sure that women's rights are structured, and, you know, are protected, but also promoted. We have seen in my country, we have/ and and I know you might be knowing about someone called Stella Nyanzi. She's a woman human rights defender, and she has faced a lot of violations. So many of the times these women human rights defenders they not have structures that are, you know, respond to their needs, to their needs, and yet they do a very, very big role in our lives. So I also think that the global health policy should also respond, and you know, at least care, and also make sure that ehm those voices are also listen to. Another thing I'm also looking at is making sure that our voices of women in the different diversity and intersectionalities are aired out in this document. Because ehm when I say LDA is a refugee, she's a sex worker, she's a queer woman, and she's a woman living with HIV. I have valuable settings to, you know violations, and also that creates multiple stigma and discrimination into my, you know, my setting. Whereby a lot of women have been struct/ silence/ silent, silently structured towards ehm, you know, being vulnerable to different violations. Whereby you are a refugee, you don't know how to speak the language probably of that country, you are a refugee you cannot access properly government ehm provisions

for the people who are supposed to be living there. So if we also (unint.) in ehm women in their different diversities and respect also their intersectionalities. That is also going to help us to respond to the different ehm needs in the global health policy. And I'm also looking at having, you know, a universal document that is going to be responding to the different needs of ehm, you know, ehm women and men in terms of equality and equity. Because when we look at the two dimensions, one side is not balancing very well because we we shall say we are looking forward to achieving equality. Even when you look at the Uni/ Univ/ ehm Un/ United Nations Development Goals you look at we are all looking at the Sustainable Development Goals are all looking at, ehm you know, equality by 2030. But where is the equity? Have we engaged men on board? Have we got the voices of men on board. Have we taken note of what experiences and what, you know, about vulnerabilities do men have in these structures where they're living in. So I really think also having men on board is very key for us, so that we can have equitable services, and also ehm achieve equality. And then also having the focus of ehm, when and wh/ when do you want to achieve it? Because when I look at the global health policy. I see it as a framework that is going to be responding to the needs of women and men. And then, in most of the circumstances we find that women apparently/ okay I really understand that men have been on the top in the past years and I'm very happy that we have really fought that patriarchal society, but it's still exists even in the political sentiments, even in the parliaments in our countries, even, you know, even in our homes you see that men are are still patriarchy. So I think the global health policy should also structure how to end patriarchal societies in in in the different settings. And also ehm look through reviewing ehm the different policies that are within different countries. In Uganda we have very, very, very punitive laws and policies. We have the the Sexual Offences Bill. We have the Anti ehm Pornography Act. We have the Homosexuality Act. We have, in our country we don't even have a sexuality education framework. We have a sexuality education framework, which is not comprehensive, and if it is not comprehensive that means it cannot respond to the needs of ehm young people or women, or (unint.) their different diversities. So you'll find out that a certain community is left out in that sense. So I think that global health policy should be structured to, you know, based on different country regiments, whereby ehm we have ehm the patriarchal settings and challenges that are within that country, and how best can we respond to them. But also looking at the ehm ehm Human Rights Declaration and also the ehm

Human Rights Review. That's recently just happened in different countries. I think we also need this document to be attached to those universal ehm, you know, documents. Now when I look at the Maputo Protocol that ehm talks about gender-based violence. And even up to now we still have women who are experiencing gender-based violence in different versions. So I really want the global health policy to be attached to those structured human rights documents. To make sure that has/ they are moving as they are structuring to review, different punitive policies. They are also looking at the global health policy as a structural, you know, ehm advantage and solution to the different human rights violations that are happening to different people in their lives, to access to sexual and health services, access to legal service because some of us in our country we can't even access, you know, legal services to because we identify differently with, you know, things of that kind. And then I wanted also to speak towards bodily autonomy and integrity (The internet connection gets worse) We have (unint.) (...)

[0:40:04.4] **HE**: Oh, I'm afraid your connection is just (...) getting worse. //

[0:40:10.1] // **LDA:** challenge where we cannot speak about who /

[0:40:15.7] **HE:** Sorry, maybe you ehm repeat that part with ehm bodily autonomy and integrity, the last part?

[0:40:21.9] **LDA:** Okay, ehm, sorry. I I was really saying, I would also want that global health policy if we are looking at solutions, I would want it also to structure ehm normalities around bodily autonomy and integrity because many of us in our countries are still finding challenges whereby ehm, sex work is illegally, ehm socially, culturally and religiously unacceptable. So we have a lot of challenges, and I'm ANre that it's only Senegal in Africa that has legalised sex work. in other countries we are still struggling. It's a battle between us, and you know the labour frameworks. So I would also work on the lab/ ehm the global health policy to structure, you know, normalities around ehm bodily autonomy and integrity. Yeah, back to you.

[0:41:11.3] **HE:** Thank you very much. A lot of very interesting and ehm thoughtful points. I would like to elaborate on that ehm a bit later. But now I just let AN answer the question.

[0:41:22.9] **AN:** Wow, ehm, thank you, LDA, for so many inspirational thoughts already. Ehm, I can only add a couple of more of my thoughts ehm on the long list that you've already ehm mentioned. Thank you for that. I think (...) I think I would like to mention, probably around three aspects that are structured around some sort of an education and

sensitisation process. I do think that we need to ehm (...) start reflecting, ehm reflecting upon these (...) many different privileges and the power hierarchies that ehm play a significant role, especially in the top-down areas of policy ehm ehm policy-making and political levels, and try to bring bottom-up and top-down to each other. Ehm, how can we do that? First of all, I think to start this sensitisation process in all of the areas, which means in civil society, by ehm edu/ starting from from the beginning, ehm with educating children and ehm going in a lot of educational (...) areas, which means not only children, but also youth and young adults to make them understand that ehm they are acting and active subjects who are ehm able to, what you just said LDA, ehm to to access and to act upon their bodily autonomy, and that everyone can be responsible to be active in society, and that they have, or can ideally, access tools to be, active in society, and to build a strong ehm bottom-up movement, I guess, of where the bottom-up movement can be linked to the top-down and the top-down being sensitised about the power hierarchies, and ho/ what consequences it has on the health in each country, but also globally. And (...) I do think that that would equate in a more ehm in more participation on every level, I think. If that makes sense. (...) I tried to bring a structure in my thoughts and try to link it to what LDA just said, but I ehm, if that was not completely clear or structured, let me know then I would elaborate on that more.

[0:44:09.4] **HE:** Yes, thank you. No, I think it was very structured and ehm very clear. And I would also like to ehm link to this bottom-up, but also top-down approach. Ehm (...) so maybe first the the top-down. Who would you consider accountable for adapting or implementing a feminist global health policy? And this can be at the global, but also at the local level. So whatever level you want to choose. Ehm yeah, who should be in charge, who is, who is accountable?

[0:44:43.8] **AN**: Oh, I love that question. (laughs) (HE smiles) Well, ehm on a on a country, I will start on a country level because that obviously then transfers on a global level as well. And because we/ the institutional level or the political level, ehm the law-making institutions, those are, I guess, the main (...) areas where we build a foundation that will last that will have an impact over years, and which is at the same time the more (...) a rigid of all. If we look at ehm policies, if we look at laws, especially, ehm for example regarding ehm abortion rights, even if we look in Germany ehm that considers itself ehm as a very, I guess ehm it is a very wealthy society, and it's also considering

itself, I guess, progressive, but at the same time we still have very conservative laws that have not been overcome. And I do think that therefore, on a political level, ehm there needs to be this very in depth, sensitisation, progre/ ehm process, and also at the same time, and that's why I'm saying top-down and bottom-up needs to be linked, ehm in all these processes there needs to be ehm more participation of everyone in society. Because we do have experts in every ehm, in every subject. We do have health experts that are in the midst of society and who can ehm decide and who should be the ones also creating ehm new approaches regarding health. And I do think inherently, especially in German society, you have this political ehm pathway where you run down. But those people making the policies are not necessarily those who know best about, for example, ehm equity and health, or ehm what's, what is what the society needs most. And therefore (...) yes, that I think that's enough. (...) For now. Maybe LDA wants to add more.

[0:47:07.6] **LDA:** Ehm yeah, thank you very much. And (...) I really think ehm having the community at the centre of the global health policy is very vital and very key because it will help to meaningfully ehm engage, but then also ehm monitor, give them space to monitor on how it is working to respond to their needs. So for me the number one actor could be community because we are the owners of this framework we all need and we have to really understand it. So community is very vital. But I don't want to ehm go ehm to to be to be different from what my colleague has said. I think also policy-makers ehm need to really structure. Because I realise in in my country, Uganda, in the parliament of Uganda we have committees, and we have committees that respond to health, respond to HIV, respond to (unint.), respond to, you know, climate. So I really think also the ehm policy-makers need to know and be ANre of this policy. Because that is when it will help us, when they are doing the different policies like on a national level, they are also in position to structure some ehm ehm slots from the global health policy and bring them as actors to help us ehm move on the same line of global health policy, and also the policies that are structured within that country. I'm also looking at having that East African Parliament because I come from Africa, but I come from Eastern Africa. So we have the East African Parliament, where I see a lot of issues are being raised on health, on climate and anything. So I also think, if we have ehm people from the East African Parliament understanding that global health policy, it's ethics and what it's going to bring, and how it's going to bring a change into our communities or into our countries could be a plus for me because I know when,

structurally in this policy, somewhere, somehow, there will be very, very con/ con/ conservative, and also co/ ehm understanding that in the global health policy there is an issue and this is how we have to address it. So if we are structured, if we are structuring this kind of policy, we need to ehm be very, very ehm determined, and also look through what the global health policy has because it is responding to the needs of us in our diversities. Let it be heterosexual, let it be homosexual, let it be a refugee, let it be a citizen, let it be, you know, an asylum seeker, let it be a worker, a (unint.) or anything, as long as it is a health issue and you are, and and you in the global health policy is in a position to respond to the needs. Yeah.

[0:50:06.2] **HE:** Yes, thank you, yeah AN go. (smiles)

[0:50:09.0] **AN:** Yeah. I would like to thank you, LDA, for all that you said. I just wanted to add one more ehm institution, I guess. Ehm, it's ehm the university and research because I did not mention that before, and ehm that's something, actually, I am passionate about, obviously, and I do think that ehm in policy-making we do need to also centre or work evidence-based, and also not forget how research is done. Ehm you did a very/ (smiles) I I loved your introduction, HE, because you mentioned it already ehm that we understand where research comes from, wh/ that there is a bias on depending on who does research and where it's conducted. And also all the policies decided needs to be/ need to be based on ehm yeah on on recent and ehm modernised approaches, which also include obviously ehm methodological approaches and intersectional ehm intersectional methodological approaches. There we go. (laughs) And yeah, that's that's very important. And that's one aspect that I wanted to add.

[0:51:26.6] **HE:** Yes, thank you both very much. Ehm, I think very important aspects you both mentioned and very key actors. And now I want to (...) because you both mentioned this, focus a bit more on civil society and community, and also because you're both active at the local level. Ehm how can these actors, civil society, social movements, how can they be included? (8)

[0:52:00.9] **AN:** LDA, do you want to start? (laughs)

[0:52:04.8] **LDA:** I beg your pardon on the question.

[0:52:07.2] **HE:** Yes, I I asked, how can civil society or the community level be included, in ehm yeah, in the decision-making process, in in the policy-formulation, or in adapting a feminist global health policy. Ehm because you both mentioned, of course, it has to be

contextualised, it has to be for the people on the ground. So how how can they actually be reached and included in the process?

[0:52:33.6] **LDA:** Okay, ehm, yeah, I I really think ehm. When we talk about a meaningful involvement, we really mean ehm engaging the community from the planning, designing, implementation, monitoring, and the evaluation of that of of that document. So to me, having ehm, us in our different intersectionalities in our different diversities is already a plus for me because at least I know your research will ehm have and influence on on on what can happen in the global health policy. Because at least you're already having the voices of the community people on ground. So this is already an initiative that I would really want us to take forward. And I, you know, have, ehm you know, probably representatives from different countries, who really understand the different health needs and challenges of different people in their countries, and also speak not only for them, but for the community because we are at the centre of the community. I as a person am not the whole community, but the community ehm trusts me that when I stand to speak I will not speak an issue for myself, but I'll speak a concern that is torturing my community where I come from. So, having representatives from different countries and different organisations is very key because the organisations are the ones, that civil society organisations are the one who are on the ground, doing a lot a lot of an enormous work. And it is very funny that sometimes we are not even recognised by the government or the Ministry of Health or Ministry of Education because ehm they think we are against them, or we are against their policies, or you know the labour (fraternity?) in the country. So meaningfully engaging us ehm as a whole, as a consortium, as a community, is very key. But having voices from each country is also very key because that is, when we shall understand, in Uganda the context is like this, and then in Nigeria it is different. And then, when you come to ehm Asia, it is different. When you come to Canada, it's this different. So I think voices give voices of community members and civil society members from the different ehm countries is also very vital that when you recognise ehm Africans recognise them as a whole. Recognise having comm/ having having ehm country hubs actually, I normally call them country hubs. Because that is when the country hubs can come together, speak about issues, and give probably recommendations, and how they can be (solved?), so that the person who is going to represent ehm them probably the global health policy framework has, and has a big understanding on what exactly that country has. Because I personally might be very i/

might be very good at speaking about issues that ehm address issues around sex works, address issues around ehm, you know, lesbian, bisexual women and queer, but I might not be very well at bring up issues that concern transgender women and men. So we need to have country hubs whereby people can come out, speak about their issues so that they can be addressed very well in the global health policy. Yeah. (smiles)

[0:56:01.4] **HE:** Thank you very much for the very concrete answer, also how we can include the civil society. Ehm, AN, do you want to add to that? (14) Can you hear me? (12) Can you hear me //

// **LDA**: (unint.) for me?

LDA: Your network is a little bit breaking.

HE: Okay, yeah. Sorry, yeah, is it (...) is it better now?

[0:56:51.8] **LDA:** (unint.) it is.

HE: Okay, thank you. Ehm sorry, so ehm I was just asking AN if she wants to add on what you said, LDA.

[0:57:00.5] **AN:** Well, everything LDA said has been (...) quite ehm concrete and very, (...) very important. Thank you for that, LDA. And I just want to add that I would ehm, I would like to have a standardised process for that. Like ehm define, for each policy-making process having ehm (...) some sort of must-do (...) access, access of, how do, how do I say that, like no, it's not called access. It's like reflecting upon how are we, how is the committee, ehm, who is in the committee, who is not in the committee, and for explicitly for that that we want to decide are we the most ehm, are we the most qualified, and especially do we have people in the in the process that are ehm not only specialised in this, but also, as you just said, LDA, are the people that we're talking about. So incorporating, I wouldn't call it quota, but maybe it is some sort of quota, I guess. Ehm, I think it is a structured and standardised (...) framework for each process of developing policies, and where we or where the committee ensures that everyone concerned is at the table. And that shouldn't be something that the committee decides themselves because then it would be probably ehm like it is right now already. Ehm, but it should be defined concretely that we want, what thirty percent of the people ehm involved be from from the bottom-up, from the communities, ehm from the marginalised ehm who are affected by. And then we want twenty percent experts from research being included in the process. Then we want ten percent what, from from the policy-making, political level, and then the

committee will always be ehm, will always be consisting of (...) everyone involved. And that would result in a process that will be far more fair and and ehm (...) and ehm mirroring society and ehm being target targeting the ones we want to reach. (...) That's actually the only thing I want to add, because LDA said everything else. (laughs)

[0:59:54.0] **HE**: Yes, thank you very much. Ehm yeah I like the idea of the committee, and of including everyone, and also including the ones affected, the most marginalised ones. Ehm as this is also what a feminist policy is about. So what do you think, what are the challenges when it comes to this, I mean, why is it not happening? Why are we not including the people on the ground enough, ehm, and everything you just mentioned? Where do you see the challenges in this process?

[1:00:27.5] **AN**: Well, first of all, I do think the whole point of reflecting and sensitisation. I don't think that this ehm is necessarily something everyone knows. (laughs) I don't think, that, for example, power dynamics, or or reflecting upon hierarchies and privileges, and how certain people are ehm are privileged enough by structures to end up in certain positions that we do have classism and racism and sexism, and all the other -isms that there are that affect people ehm (...) landing in deciding positions. And I don't/ do think that this first of all knowing about it, ehm would help. (laughs) So therefore the educational approach and second of all, of course, those in, and it sounds now very, very activisty, but those in power obviously ehm want to cling to it, and those speaking still want to be heard, and I do think that ehm it's important to let everyone know that we all will profit from an approach where everyone gets heard, and that it doesn't mean that those who have been heard now are less heard, it's just like everyone else is also included. (laughs) And I think that's for example something that's splitting the whole feminist movement. Ehm for example, the whole discussion upon ehm, who is a woman and who's not a woman and ehm trans-including and -excluding, and everything. Ehm it's about diversifying and letting everyone live, I I don't have to tell you. (laughs) Well, you know what I'm saying. (HE nods)

[1:02:19.2] **HE:** (...) Yes, definitely. Thank you very much for making that point clear again. Ehm LDA, do you want to add what challenges, you see?

[1:02:33.4] **LDA:** Ehm the challenges I really see are (...) The challenges I really see are around the funding because the global health we see it, the global health policy is a framework that is going to be used ehm worldwide. So challenges in funding, especially in ehm low income

countries like Africa, within my country Uganda is a challenge, I really see that there is inadequate funding for this ehm kind of framework to be administered. Especially when it comes to meaningful engagement. Because when I talked about the country hubs I meant having them in the country, in every country that we're having a a country hub. And we get the concerns and issues of that country and then we get someone to present them. So, that kind of system might not be in position to be structured because there is no funding to facilitate people from, you know, different regions in the countries to come together and, you know, ehm be at the same place. And then something else, I look at ehm the policies ehm, and frameworks build within our countries. Because when I look at Uganda it might be very hard, very difficult for us to adopt the universal ehm, you know, the global health policy. Because even at the Universal Human Rights Periodic Review has been a very challenge for us to have it in our country. So it's very difficult for us to adopt the global health policy because ehm of the policies are ehm and other structures around. I will give an example. In Uganda we normally have an event, called the Nyege Nyege Event, it always happens in September. And here we are, the Parliament yesterday sat and said it will not happen this year because we/ they are promoting immorality. And when they talk about immorality they are talking about the homosexual persons. So for them they are saying that there will be promoting gays and everything, which is not true. So for us as people we know, this is a fun event, everyone is ehm is available to have fun, to enjoy. But for them they're having it in a different perspective. So I'm very sure, it could also happen to the global health policy, whereby they will be like we are promoting sex, ehm you know, homosexuality. And so the problems within our country are also very, very structured. And when I look at our cultural, social, religious, and ehm ehm legal environment, they are not really welcoming. In our country, Uganda, we have a a tribe that has to do male circumcision to a male child when they are around twelve years. Now me as a mother who has given back to my son, I want to circumcise my child at the months at at an age of three months. But because I have a culture that is not allowing me to do that I'll wait until that child next twelve years, which is, you know how structured our community is. And now I'll give an example of the Sebei people who are, do female genital mutilation. This is very, this is one of the acts that is very harmful to women. And because it's a cultural setting you'll find women, and and, you know, men giving them their children to be done on female genital mutilation because it's a cultural setting. So culturally,

socially, and you know, ehm religiously and legally, we might not ehm ehm achieve the global health policy because of that kind of setting. Another thing that I really wanted to talk about is, ehm (...) if we have not engaged intergovernmental, ehm you know, settings. It is also going to be very difficult for us. Where is the Ministry of Health in Uganda, where is the Ministry of Health in Germany, in Italy in, you know, in Canada, in Kenya, in in any country. We need to and get this (unint.) to understand this kind of policy and what it is aiming at. Because I'll, I'll give an example of the UNAIDS, United Nations Aids Strategy. Whereby we are saying ending HIV, sorry <u>Aids</u> by 2030. And this is something that has been adopted by every country. You see, all countries are doing a lot of efforts to make sure that they reach the 95-95 strategies and targets. Here we are. Have we involved the relevant, ehm, you know, ehm government societies. Have we really involved the people that rule, you know, the policies in the countries? Because this is what we have to get intact. And make sure that at least the policies in Uganda (unint.), and this is what we have in the global health policy. So I really really think those are the key issues I would really look out at that point. Yeah.

[1:07:40.4] **HE:** Thank you very much. You mentioned so many aspects from the local, but also the global level. And I hope we can touch on all of these. And ehm yes, so now I would ehm consider the global a little bit more because you also said <u>funding</u>. And (...) because this is also part of my thesis, I think it's also very closely linked to colonialism on coloniality. So I'm also wondering how can a or what can a feminist global health policy do with regard to decoloniality. So what could be alternatives if we consider this at a more global level? Ehm, I mean, funding would definitely be a major aspect, but maybe you also have different ideas. (11) So, ehm LDA, I don't know if you want to add on this directly? (16) I hope you can still hear me. (20) Can you hear me, LDA? (...)

[1:09:32.0] **LDA:** Yeah, I can hear you.

[1:09:34.1] **HE:** Okay, great, because I/ we also lost AN. But now she's back. (laughs) So I wasn't sure if it was my internet connection.

[1:09:42.7] **LDA**: Sorry, sorry, sorry. I beg your pardon in your question.

[1:09:46.7] **HE:** Don't worry. Ehm, I just want to make sure that AN is back with us now.

[1:09:52.1] **AN:** I am. Thank you.//

[1:09:53.5] **// HE:** Okay, great. (laughs)

// **AN:** (unint.) of that question as well but then you stopped at some point and

then I/

[1:09:59.2] **HE:** Okay, I don't know what's happening here today. I'm sorry. Ehm yes, I wanted to add this asp/ aspect of the decoloniality ehm because ehm, as we also just mentioned, or LDA mentioned with ehm funding and ehm, all these multilateral institutions that are meant to be global. But there's still some colonialism inherent. So what alternatives can a feminist global has policy provide with regard to decolonialism or decoloniality? (10)

[1:10:41.4] **LDA:** Ehm, when when it comes to our issues around decolonialism, we we, you know, many of our countries have been colonised, (...) and this is a very big aspect that we really need to take note of and make sure that ehm we are, you know, coming out with the the decolonialism aspect. I'm trying to look for something on my laptop, I don't know whether/ Okay in Uganda, we have a feminist, she's called Dr. Sylvia Tamale. She has written a book about decolon/ (laughs) decolonialism, it's what I'm really trying to look for, it's a very good book. I've read it and it has really opened up my m/ my mind. (...) And ehm when when we do not ehm, you know, as I I liked what my fellow speaker talked about, engaging the community, sensitising the community. When we do not sensitise the community, we shall not get an opportunity, of ehm helping them get out of, you know, these different vulnerable situations that they are going through. So, let alone LDA who is very conversant, who is speaking, she knows the issues, she can explain that issues. And then there are those people who are on the ground, the far to reach people who are, you know, in Uganda most of the sex workers are (immigrants?), so we need to make sure that these ehm documents are translated, are put into easy to read languages, easy to read, ehm you know, terms. We need to make sure that when someone gets the global health policy, they are in position to understand it better. (...) I don't know whether you're getting my point (HE nods) So we need to make sure that people understand these points very easily, (...) and they are not criminalising. They are not stigmatising, not even, you know, discriminating any of the communities because sometimes we do not understand that the number that we use many of the times is, you know, punitive to the different communities. It might be very punitive to sex workers, and it might be very, very okay to, you know, another community. Now, when it comes to the trans community, it is very worse. Because I don't know people/ you might find a a LDA who wants to be, who prefers to be called Enock, and then, because for you, you're seeing sh/ she physically, but when someone tries to explain to you, I don't want to be called she, I am called Enock. Please

let's adopt to that. So if we do not sensitise the communities and also structure languages that communities can understand. Let's jump from I I I normally call it a medical language. You know how medical personnel can speak languages that are very difficult to understand. That is what I'm trying to mean. So we need to get ehm languages, words that our communities understand much more better. Yeah. Back to you.

[1:13:49.2] **HE:** Yes, thank you very much, ehm very important aspect that it's actually understandable for everyone, and especially the people we want to reach. So, AN, do you want to add on that?

[1:14:05.0] **AN:** Hmm. Well, I'm thinking what I could add like ehm because of the (...) the reflection process, I think I've said (laughs) like a million times already, ehm involves also understanding the colonial past, and also reflecting upon (...) what responsibility comes with that. Especially from the Global North. And therefore (...) being very proactive in (…) providing resources and making sure that everyone sits at the table, everyone is allowed to decide the same amount. Everyone can, from from ehm every level of society, is capable of acting independently. And what LDA already said, funding is key, communication is key. Ehm and also this realisation that there is a responsibility, I think that's the one thing I want to add as well. Ehm that inequalities didn't just happen, and they are not just here like that. And therefore ehm helping everyone become as independent as possible. And ehm taking on that/ tackling that responsibility, I think that's that's very important as well.

[1:15:45.1] **HE:** Yes, thank you very much. And do you see, if, like these multilateral organisations, for example, the World Health Organization, or UN Women, or any other of these organisations, can play a role in a feminist global health policy? Or is it rather the nation states? And then the community and very contextualised ehm specifically? Or is there also like this very global universal approach, maybe one of these organisations could take up. What, what do you think about that?

[1:16:24.7] **AN:** Ehm I actually think everyone should be involved, and everyone can, and especially regarding institutions like UN Women and WHO. Those are the places where the resources sit as well. So, therefore, of course, ehm it should be also the responsibility of organisations like that to ehm to access their own resources, to then go on the national level and in the communities and work together. Like, just just as we mentioned in the in the in the far beginning, ehm the whole top- down bottom-up approach doesn't work if we're

not interacting and intertwining every every level. So yes, I do think that works. But also, (laughs) ehm if I can be a little sarcastic here, ehm those institutions need to, (…) need to get in the field a little bit more, and not only stay theoretical. If that makes sense.

[1:17:30.3] **HE**: Yes, so what would you expect of them if you say they should be in the field more. Is there anything concrete you have in mind?

[1:17:40.3] **AN:** Well, actually, what you just mentioned before, like on the national level, and ehm as/ accessing resources, and providing ehm providing resources and (...) then implementing (...) policies and seeing it through up until the end. But also s/ having it as an interactional process like a never-ending process where also the community's feedback, I think it should be like a ehm yeah, that that's about it. Yeah, if that's concrete enough.

[1:18:17.8] **HE**: Yes sure, thank you. Ehm maybe, LDA, you also have an opinion about the the global level, i/ if you want.

[1:18:25.4] **LDA:** Ehm I think ehm agencies like the UN Women, UN itself, UNHCR are very vital and ehm because they provide the platform on a global level, but then also they provide funding. Because I've seen UN Women in my country are supporting work around gender equality and equity and that is a lot of their passion. So providing, their role should be providing funding and also providing the platforms that ehm the global health policy is, you know, adapted in different countries. Ehm and then actually also, everyone should be engaged, as my other colleagues said, because it's very key for us to have everyone's voice. We don't need to lea/ We don't need to leave anyone behind. Recently, when we were in Canada for the International Aids Society Conference. We we we realised that ehm a lot of people we denied visas. So our work was around advocating for you know, people getting visas, and we were saying, no visa, no voice. And what we were meaning was, ehm some people have not been able to come, and we are not going to get their voices. So if we do not get visas, we shall not get the voices that we need. So here we come. No inclusion, no voice. We are not going to get a full holistic package, a comprehensive package in the global health policy if other people, if other categories of people are being left out. Yeah.

[1:20:11.4] **HE**: Yes, thank you very much. Ehm that's so true. And yeah, I also followed the ehm the Aids conference, and the problem with the visas and I think it's ehm yeah, very, very important issue, and we should not have this problem in 2022 anymore, I guess. Ehm (...) Yes, so now ehm, maybe to make it a bit broader or open it up, I was just wondering if you

could make reference to your like very personal, or very daily experience like, where do you see the benefits of a feminist global health policy for yourself, I mean, AN, you're a trained medical doctor, ehm so maybe you can tell us something about the more medical sid/ ehm side or the more clinical side. And ehm yeah, LDA, you're also from a sex worker background, but also from the activist background ehm li/ to have like some ehm concrete on the ground experience where you think this would really provide a benefit for the situation. If if you want to do so, of course ehm, yes.

[1:21:27.2] **LDA**: Yeah, ehm the global health policy will really benefit a lot of people on the ground. I'll give an example. Ehm people living with HIV, many of the times there's a lot of challenges. Ehm and these challenges are not really really ehm responded to because of different issues, probably funding, or that is not the focus, a priority that we are giving. I liked it when UNAIDS ehm last, I think last year but one of their themes was Ending Inequalities. And with, I think this global health policy is going to help us end the inequalities that are around the whole structures in different countries. So to me, I see the policy coming, with the framework coming into the country and ehm ehm the framework coming into the country, and you know, giving guidelines, giving guidelines to our health service providers. But then also we civil society organisations, and we activists, who advocate for these ehm services to be provided to the people. I recently, I I'm an HIV Prevention Advocate. (smiles) So recently I carried/ I led/ ehm a a a peaceful demonstration around accessibility and availability of the dapivirine vaginal ring, which contains PrEP and it is is in/ inserted ehm in a woman's vagina. And we found out that Pepfar had pulled out of the funding in Africa. So our main issue was to advocate for the availability and accessibility of this ring. And it was a very successful ring, and one of our aims was to request the UNAIDS Director Winnie Byanyima to be a Global Ambassador. And (unint.) I'm telling you with a lot of happiness on my face, she committed to be the ambassador of the ring globally. So I am seeing the global health policy, you know, ehm affecting, bringing an effect on such, you know, positions whereby donors have pulled out in funding a category of (unint.) services or, you know, a category of populations. And then we are struggling and telling them this is what we have in a global health policy. This is an issue that is affecting this country and this country needs support. So to me, I really think the global health policy would help, help us structure and when it comes on community level, I see ehm people's, you know, quality of

life improving because I know there will be an improvement in health provision. And when I go back to where I started from speaking in the first place, on this call was, ehm you know, making the Universal Health Coverage a reality for everyone. When we talk about affordability, accessibility, equitability, availability and all along, we need to make sure that the Universal Health Coverage is on the ground to hold people accountable. Because if I come into your office and I'm a trans man, and I want a service, do not look at my sexuality, do not look at who I am. Look at what you are supposed to be providing for me. Do not look because I am not, I'm a refugee, I'm not a Ugandan, I'm from Kenya, as I am. And, you know, and those are the structures that are under on the community that are really, you know, diplomising us to access the services. So I see that global health policy coming in to interact with the structures that are available in the country and influencing them to make sure that people have access to comprehensive and holistic health services.

[1:25:15.2] **HE:** Thank you so much for giving these examples, and also congratulations on your efforts with ehm the UNAIDS director. (LDA cheers) It's really nice. (laughs) Yeah, AN, you also maybe want to share some thoughts?

[1:25:36.1] **AN**: Thank you, LDA, for sharing that. That was very ehm nice to hear. Also the vision of accessibility and access to a healthcare that is holistic and works for everyone. Ehm (...) your question was, how would this affect work and just like specifically in the medical field for me as a medical doctor, for example in the clinical practice. How, because I mean it's different areas that would be affected, I guess. Do you mean just as a question to you, HE, again, because there's so many ways to answer this this question. Ehm what exactly do you mean? Do you mean some sort of a vision of a future? How that could look like if ehm, we have a holistic intersectional approach in a society where everyone has an access to healthcare, and everyone has looked at the same? Or what ehm is the question?

[1:26:42.3] **HE:** Yes, I mean, you're free to I answer it, ehm any way you want to. But ehm, yeah. So if you think about your area of work now. I think you also because you're the co-founder of ehm fem/ Feminist Medicine, you see see probably some very deep structural challenges, and how you think a feminist global health policy could maybe be a solution or an alternative. And if you just could give some examples, what could change, maybe, and it can be very broad, but it can also be like very narrow that ehm, I don't know you worked with this ehm

old white man and I don't know (smiles) it's so, it's, it's really up to you, what ehm, how you want to answer the question.

[1:27:27.0] **AN:** Ehm, all right. Well, first of all, the whole educational part of it, ehm in our every worl/ I mean, that's the wh/ most important part of our work of the organisation is that ehm (...) we produce a lot of content where we want to educate and talk to everyone about these problems that exist about the discrimination in the healthcare system. And I do ehm (...) believe that (...) ideally ehm through this reflection process ehm people start being angry. And, ehm for example at my last workplace by bringing up these issues ehm there was some more sensitisation towards ehm towards patients, for example with different backgrounds, because the team was super ehm white, and had a very Eurocentric approach, and ehm was very ehm not sensitised in terms of sexism or racism. So in the medical practice, ehm by including a critical view on our healthcare systems ehm locally and also globally, but I'm talking about locally now. Ehm I think once you put on the glasses, then you're not able to not to, to unsee it. And therefore this sensitisation or educational process is key, I believe, and (...). So I hope by, by making people realise how the inequality, how inequality ehm exists right now, for example, doing a pers/ a presentation on intersectional feminism in the healthcare system right now. Ehm, for example, that ehm that the pulse oximeters, I'm not sure how you call them in English in an easy way. It is the the the ehm the instrument that measures oxygen in the blood. Ehm that that measures oxygen ehm differently, and and on black and brown skin, for example, in comparison to white, and that that could result in ehm not detecting less oxygen in the blood and therefore are bringing my patients in danger. These are facts that we know of recently. So, therefore, ehm through this whole sensitisation process that goes in different areas, ehm that concerns the research area, where we will find a lot more when we ehm start looking. But we haven't started looking ehm adequately yet. And then ehm this sensitisation process in the medical education, where from the beginning, everyone starts to rethink on what are we taught, and who has ehm, who has shaped medicine and medical curricula throughout the ages. And ehm having this critical view upon it, that will also change how we practice medicine in the future ideally. So the clinical practice in a couple of years and many, many years will be different if we, if we adapt this process, this critical way on thinking on health, health systems, on knowledg/ generating knowledge, and on who profits from what. Ehm that is key in every area, and in the end, ideally, the patient will profit

from it. Ehm but I do think that's a very long process, and still in the everyday practice (sighs) there's still a lot of inequalities to tackle in many areas. So, yes. That's what I mean to say.

[1:31:35.8] **HE:** Yes, thank you for sharing these ehm medical insights with us to. Ehm and now I just want to ask you because you mentioned already quite a lot ehm, I just want to ask you if if you want to add something that we haven't touched on so far, something you consider important. Maybe that's something I didn't really, ehm yeah, think of so far. Ehm it can be regarding challenges, it can be regarding solutions or alternatives. Ehm something you think this should be in the discussion. Then you can tell me now. (11)

[1:32:22.2] **AN:** Thank you, HE.

[1:32:24.3] **HE:** Okay, okay great. Then ehm yeah. I mean, it was kind of also already included in your last answers. But I just want to have this this pos/ positive outlook. What are your hopes, or, as you said AN, your visions regarding a feminist global health policy for the future? Ehm maybe it's also a bit redundant with what you've already said, but ehm just to wrap it up. (8)

[1:33:02.3] **LDA**: Yeah, my, my hopes are (...) I am very sure that the global health policy framework will ehm improve ehm the quality of services that are provided in my country through making them comprehensive and holistic. But then also ehm ehm many of the vulnerable communities will be, you know, integrated into those different ehm, you know, approaches. And it will also have a space for women human rights defenders because they also do a very big role. Activists do a tremendous job. The advocates that we have are very, very vital when it comes to our security, and you know presence. So I think it will also respond to the challenges that it's/ they experience in one way or the other. And ehm I am very sure it will promote gender i/ or equity to achieve gender equality. Yeah.

[1:34:09.5] **HE**: (...) Thank you very much. Very nicely said. (smiles) AN, do you want to share your vision with us?

[1:34:18.9] **AN:** LDA had a wonderful vision already. (laughs) Ehm (...) Actually, my my vision or my hope is that one day we we don't need to talk about this anymore. And I ehm (...) But until that point I really need and want and wish for ehm everyone making this holistic and intersectional approach to healthcare their priorities because it is our responsibility, and it is what we (...), what we live with it. And therefore, I don't/ I I wish for a future where that's a priority, and where everyone understands that the more diverse we

are, and the more everyone has an equal oppor/ opportunity, the more (...) healthy in all aspects society will become. (...) That's it.

[1:35:29.1] **HE:** Yes, thank you. Thank you, also very well said. Ehm yes, I think we touched on so many aspects that I have a lot of (laughs) food for thought, ehm there's just something I ehm I want to ask you and because with the other groups we decided that we will share the videos ehm of the recordings. So ehm but I have/ I mean everyone has to agree, but if you agree, then I can send you the other videos, and they will also get this one. So everyone sees what the other group talks about. And because you are part of this this project. If you agree, I can do that.

[1:36:14.9] **AN:** Sure, no problem. (...) That's wonderful.

[1:36:19.4] **LDA:** No problem!

[1:36:20.2] **HE:** Okay, great. Great, thank you. Ehm yeah, then I will share with you the videos of the other groups afterwards. And of course also, as I said, ehm it will probably take some time, but when I have the results, and I wrote everything down, I can also send it to you, and then you can see if, if you feel misunderstood or something. And ehm give me feedback. I mean you don't have to do this because it's also a lot of work. But if you want to ehm, I will definitely include this in my thesis, so that everyone who wants to adjust something can do so. Ehm yeah, just to let you know. (laughs) (...) Okay, yeah, then I don't want to keep your time any longer. Ehm I think I haven't heard from NK, it's a pity she couldn't join us. But ehm, yeah, I really enjoyed the the interaction with you two, and you had so much to say already. So thank you a lot for taking the time also because it's quite late now, especially for LDA. Ehm so thanks a lot, and I really hope we stay in touch.

[1:37:27.4] **AN**: Thank you so much HE, for initiating this and for the invitation. And it was very wonderful and insightful to meet you, LDA. Thank you.

[1:37:46.6] **LDA:** Oh wow, it was also nice meeting you too. Thank you very much for ehm making me be part of the conversation, HE. I know we have had a very big discussion. It's it has been quite long. (laughs) It's 8:30 PM in my country, so it's it's it's night. But you know the life/ you guys should come to Uganda, the life in Uganda. is very fun. (laughs) So, yeah. I'm very happy, I'm a very talkative person (unint.)

[1:38:06.1] **HE:** Thank you very much. (laughs) Okay.

[1:38:15.9] **LDA:** Bye bye. (waives and smiles)

847	[1:38:16.8] **HE:** Good night, bye. (waives and smiles) (AN waives and smiles)
848	[1:38:18.7] **LDA**: Good night. (laughs)
