# Supplementary material for: Towards a Feminist Global Health Policy: Power, intersectionality, and transformation
Source: PLOS Glob Public Health. 2024 Mar 7;4(3):e0002959. doi: 10.1371/journal.pgph.0002959 (PMC10919653; doi:10.1371/journal.pgph.0002959)
Supplement: S3 Appendix — (PDF) [file pgph.0002959.s003.pdf]

Annex S3: Code system

Code	Definition	Scope of application / Demarcation from other codes (*if applicable*)	Anchor quote
Formal codes			
**Information about participants**	This formal code is applied when participants introduce themselves in the focus groups.	It is not analysed separately but taken into account when analysing the other codes.	“Ehm so my name is MM, my pronouns are she and they. Ehm I'm a social scientist, so trained mainly in anthropology and sociology. Ehm I'm originally from France, but I studied yeah I've done my PhD studies in Canada. I also worked in the UK and now I'm working in Belgium. Ehm (...) Yes, I've worked mostly like on health inequities, discrimination ehm yeah in my whole life. So I'm coming from a research background more. Ok, that's it from me” (FG1, MM, 69-74)
**Nonverbal & Interaction**	This formal code is applied for nonverbal expressions or interaction during the focus groups.	It is not analysed separately but taken into account when analysing the other codes.	“(AN waives and smiles)” (FG3, 27)
Thematic codes			
**Challenges in Global Health**	This main code entails the major current structural challenges within global health.		

Code	Definition	Application / Demarcation	Anchor quote
Privatisation & capitalism	This subcode presents the consequences of privatisation and capitalism as a current challenge in global health.	This subcode is interlinked with several codes. The great importance of privatisation and capitalism in global health and the explicit emphasis in the focus groups require a separate code.<br>(For further distinction, please refer to the codes "Private sector" and "AAAQ of healthcare")	“my immediate thought was that the biggest structural challenge in global health ehm is the role of the unregulated private sector ehm in the delivery of health care ehm and the corresponding myth that the private sector is more competent to deliver healthcare than the state” (FG1, JC, 217-21)
Structural inequalities	This subcode presents structural inequalities as the core of various challenges in global health.	This subcode is interlinked with several codes as structural inequalities constitute both the cause and consequence of further challenges. Since "Power regimes" determine "Structural inequalities", these codes are closely interrelated. However, to depict the structural causes and the resulting inequalities as fundamental challenge in global health, the subcode "Structural Inequalities" is applied.	“It's also about how can we dismantle this ehm this ehm this structure, this dominant structure that perpetuates inequality, and perpetuates ehm very oppressive, discriminating, ehm and repressive health policies.” (FG1, CJ, 635-37)
AAAQ of healthcare	This subcode presents current challenges related to accessibility, availability, affordability, and quality of healthcare.	The code relates to the code "Enhanced health and healthcare", while emphasising the current problems and challenges instead of	“We need to focus more on the accessibility, affordability, ehm equitability, and the availability of the services. It is very, very unfair for us to have services

Code	Definition	Application / Demarcation	Anchor quote
		anticipated improvements. It is further linked to the code "Privatisation & capitalism", but "AAAQ of healthcare" encompasses more than the financial aspect and was specifically mentioned as a challenge in the focus groups. "Privatisation & capitalism", also emphasised in the focus groups, entails further aspects not related to accessibility, availability, affordability, and quality of healthcare.	that ehm, I'll speak in regards to my community, where my community cannot afford the services many of the times in my country.” (FG3, LDA, 192-95)
Lack of political will	This subcode presents the lack of political will and the preference to remain the status quo as a current challenge in global health.	This subcode is related to "Power regimes" and "National policymakers", but focus is on the explicit challenge that insufficient action is being taken and no CJe is sought.	“I mean, it's this idea of like, if you're not part of the solution, you're part of the problem. If you don't have the political will, and you're really not ready to see why it's needed to have this this this power shift. Then it's because you probably are the ones that are gate keeping or blocking that from happening.” (FG2, OLU, 516-20)
Quantitative & biomedical focus	This subcode presents the narrow scope of a quantitative and biomedical focus as a current challenge in global health.	Parallels exist to the codes "Knowledge and discourse", "Education within the health system", and "SDOH & contextualisation". However,	“also stop seeing health only in terms of hospitals and tests (OLU nods) and medicines, and you know all the rest of it. So ehm you know, it's just the way

Code	Definition	Application / Demarcation	Anchor quote
		the code has its own focus and is emphasised as a challenge in the focus groups.	that health is viewed itself is still narrow and ehm also that the people who have been doing the work for so many years, and have the year to the ground, are not respected.” (FG2, SC, 540-43)
Example of Covid-19	In this subcode challenges using examples of the Covid-19 pandemic are compiled.	Concrete examples were given with regard to persisting inequalities and the narrow biomedical scope in global health, which relates to the codes "Structural inequalities" and "Quantitative & biomedical focus". However, the example of Covid-19 sets a specific focus.	“If you frame Covid-19 as an epidemic, as strictly a biomedical phenomenon, you get the kinds of interventions that ER was talking about. You get physical distancing, you get hand washing, you get vaccines. That's nothing against those behavioural and pharmaceutical interventions. But if you widen the lens and look at Covid-19 as a result of hazardous work, as a result of crowded and substandard housing, as some, as a, as a complex phenomenon whose burdens (coughs) are distributed unequally in in society. Then you get a different (coughs) set of interventions (CJ and ER nod), a much more structural set of interventions.” (FG1, JC, 496-504)

Code	Definition	Application / Demarcation	Anchor quote
**Power regimes**	This code is applied when references to power regimes are given. This includes the description of mainly problematic power hierarchies in societies and their effects as well as who holds power. It also entails an alternative perspective on why and how existing power regimes can be CJed.	The problem-related aspects indicate linkages to the codes "Privatisation & capitalism", "Structural inequalities" and "Lack of political will". The positively connotated text passages partly align with the codes "Meaningful engagement", "Community and civil society", "Equality", and "Radical transformation".<br>The codes "Power regimes" and "Structural inequalities" are inherently related since power hierarchies create structural inequalities. However, the application of "Power regime" as a code on its own can be justified because of its overall significance to this topic and its emphasis in the focus groups. Moreover, not all text passages are double coded because "Power regimes" does not necessarily relate to inequalities as this code also portrays alternatives and content about power itself.	“the point I had wanted to make really was picking up on the idea of power and, and the way that kind of power inequities are really baked into the global health architecture (laughs), and in part because of it's history, but also because of the way that we continue to perpetuate them either as individuals or institutions.” (FG1, ER, 292-96)
**Intersectionality**	This code contains content related to intersectionality. It is	The concept of intersectionality is closely intertwined with power.	“So racism, capitalism, and patriarchy ehm (OLU smiles)

Code	Definition	Application / Demarcation	Anchor quote
	applied if intersectionality is specifically mentioned or if intersectional aspects are circumscribed or exemplified.	Hence, the codes "Intersectionality" and "Power regimes" often appear together. Intersectionality is also linked to the code "Structural inequalities". Due to the relevance of this concept for research and practice and the explicit naming of intersectionality in the focus groups, this code is included.	anything that we're going to do in terms of feminist policy ma/ne/ needs to take into account those things and needs to intervene at the point where we're not just thinking about ehm ehm women ehm as a as as a as a universal issue, but rather the intersectional issues of which kind of feminist policy we're talking about.” (FG2, SM, 367-71)
**Knowledge & discourse**	In this code all statements or references to knowledge and discourse are considered. This implies the abstract, meta-level regarding questions about knowledge paradigms as well as the direct mentioning of knowledge, discourse, and language.	Questions around what counts as knowledge, who shapes the discourse, and who is listened to are related to the codes "Decoloniality", "Power regimes", and "Meaningful engagement". However, the code has its own focus and is emphasised in the focus groups.	“That our notions of excellence, and how we recognise excellence are so wedded to one particular way, predominantly kind of an Anglo-European approach or a Western approach of what evidence is ehm that we discard vast amounts of really important data when we're thinking about effects in health policy” (FG1, ER, 421-24)
**Feminism itself**	Aspects and issues concerning the feminist movement itself, including the existence of multiple feminisms, are entailed in this code.	The history of feminism and current challenges correspond with the codes "Power regimes" and "Intersectionality". However, the code has its own focus and	“we'll be talking about feminist approaches, yeah, or approach. But I I think they are plural (ER nods heavily), and like for Europe and and North America it's very white dominated, you

Code	Definition	Application / Demarcation	Anchor quote
		is emphasised in the focus groups.	know, by yeah middle-aged educated white women." (FG1, MM, 741-44)
**Objectives & principles of FGHP**	This main code entails the objectives of FGHP and the fundamental principles this policy is built on.		
Enhanced health and healthcare	This subcode depicts the positive impact of a FGHP on health and healthcare and thus one of its main objectives.	Parallels exist to the code "SDOH & contextualisation". However, the code has its own focus and is emphasised in the focus groups. "Enhance health and healthcare" is the objective with "SDOH & contextualisation" rather depicting one component to achieve this.	"So I see that global health policy coming in to interact with the structures that are available in the country and influencing them to make sure that people have access to comprehensive and holistic health services." (FG3, LDA, 728-31)
Reproductive justice	This subcode is applied if reproductive justice is mentioned as a crucial objective and significant example of FGHP, including sexual and reproductive health and rights and bodily autonomy.	Parallels exist to the codes "Enhanced health and healthcare", "Human rights", and "Equality". However, the code has its own focus and is emphasised in the focus groups.	"how do we place specifically sexual and reproductive health. How do we ehm give that control of the women so they can also control a little bit more about their lives, and their bodily autonomy." (FG2, OLU, 329-32)
Human rights	This subcode presents human rights as a central objective and principle of a feminist global health policy.	Human rights are closely related to the code "Equality". However, both concepts are important as independent codes.	"we have to think about ehm health being a right, and you know part of our human dignity and well-being" (FG1, CJ, 450-52)

Code	Definition	Application / Demarcation	Anchor quote
Equality	This subcode presents equality as a major objective and fundamental principle for a feminist global health policy.	Parallels exist to the codes of "Human rights" and "Democracy", but the emphasis and different focus of each concept highlights the necessity of independent codes.	"I'm also looking at having, you know, a universal document that is going to be responding to the different needs of ehm, you know, ehm women and men in terms of equality and equity." (FG3, LDA, 273-75)
Democracy	References to democracy and its importance for FGHP as well as the threat of authoritarianism are compiled in this subcode.	Democracy entails the aspects of "Human rights", "Equality", and "Meaningful engagement". Therefore, strong linkages exist between these codes. However, it is important to mention democracy as a political system in addition to the components and principles it comprises.	"I think that political leaders who ehm are not held to democratic checks and balances are much more likely to enter into sweetheart deals with corporations to be seduced by corporate lobbying to be bought, and so the erosion of democracy and the erosion of political checks and balances, and the balance of power between the executive branch, the legislative branch, the judicial branch is so profoundly linked to ehm the kind of corporate capture of healthcare" (FG1, JC, 241-46)
Decoloniality	This subcode presents decoloniality as a central objective and principle of a feminist global health policy. It is applied if either coloniality and its mechanisms are mentioned	The code "Decoloniality" is related to "Intersectionality" and "Power regimes" as power hierarchies are inherent in colonialism. However, the code has its own focus and is	"So I think the first thing we've got to do in that imagination again is that we must acknowledge that all of us are deeply colonised." (FG2, SM, 693-95)

Code	Definition	Application / Demarcation	Anchor quote
	or if endeavours towards decoloniality are depicted.	emphasised in the focus groups.	
**Components & implementation of FGHP**	This main code entails the relevant components of a feminist global health policy, including necessary steps and actions to achieve the anticipated impacts.		
Radical transformation	This subcode outlines that a feminist global health policy requires a radical transformation.	"Radical transformation" is inherently linked to a shift of "Power regimes". However, this code emphasises the radical element as relevant for FGHP and how this transformation is possible.	"Let's burn it all and start all over. Let's see if that works" (FG2, OLU, 669)
SDOH & contextualisation	This subcode comprises statements about the importance for FGHP to consider the social determinants of health and context.	Parallels exist to the codes "Meaningful engagement" and "Enhanced health and healthcare". However, the aspects of contextualisation and the social determinants of health are emphasised in the focus groups as crucial components and therefore require a separate code.	"I could not agree more that feminism, takes us ehm from a narrow focus on biomedical solutionism to a broader focus on wellness and health as a complete state of psychological, physical, mental, spiritual well-being (CJ nods), and an emphasis on the social determinants of health." (FG1, JC, 485-88)
Meaningful engagement	This subcode describes meaningful engagement of civil society and the community as an	This code is related to "ANreness raising", "Community & civil society", and "SDOH & contextualisation". While these	"When we talk about a meaningful involvement, we really mean ehm engaging the

Code	Definition	Application / Demarcation	Anchor quote
	integral component of FGHP and what that encompasses.	codes closely align, the code "Meaningful engagement" emphasises the aspects of participation and inclusion.	community from the planning, designing, implementation, monitoring, and the evaluation" (FG3, LDA, 433-35)
ANreness raising	This subcode presents the need of ANreness raising and related concepts as crucial for FGHP.	This code is linked to the codes "Meaningful engagement" and "Community & civil society". However, the code has its own focus and is emphasised in the focus groups.	"I do think that therefore, on a political level, ehm there needs to be this very in depth, sensitisation, progre/ ehm process, and also at the same time, and that's why I'm saying top-down and bottom-up needs to be linked, ehm in all these processes there needs to be ehm more participation of everyone in society." (FG3, AN, 368-71)
Education within the health system	This subcode is applied when the need to CJe the education within the health system and workforce is mentioned as an important component of FGHP.	This code is closely linked to "ANreness raising". However, "Education within the health system" refers explicitly to the training and content taught to the health workforce, while "ANreness raising" is less about training and more about sensitisation and consciousness-raising approaches in the broader society.	"another challenge that I would also like to touch upon is really the entire medical education and the way in which this education is given. So you know the principles of human rights, and all of that are not taught to medical practitioners" (FG1, SKY, 273-76)

Code	Definition	Application / Demarcation	Anchor quote
Shift in funding	This subcode includes challenges and disadvantages of the current funding in global health, why a CJe is needed, and what it should encompass.	The code relates to the code "Privatisation & capitalism", but the focus is on presenting alternatives and considers further aspects besides privatisation.	"I think more policymakers, more people sat in funders that have positions of power within global health that subscribe to and live feminist values is really important." (FG1, ER, 728-29)
Orientation towards existing structures	This subcode is applied when existing documents and structures are mentioned which can be used as a reference for FGHP.		"So I really want the global health policy to be attached to those structured human rights documents." (FG3, LDA, 306-08)
**Actors in FGHP**	In this main code all relevant actors for a feminist global health policy are listed and information about their role and responsibility mentioned.		
Community & civil society	This subcode compiles statements about the role of the community and civil society as actors in a feminist global health policy.	This code is related to the codes "Meaningful engagement", "ANreness raising", and "SDOH & contextualisation". However, focus is on the community and civil society as actors and their function for FGHP.	"for me it is really community-based organisations (CJ and MM nod). Which have or follow feminist ideology and principles (JC nods). They, I feel, are key. Because they are the ones who are in the community." (FG1, SKY, 657-59)
National policymakers	This subcode compiles statements about the role of national policymakers and states as actors in a feminist global health policy.	Parallels exist to the codes "Lack of political will", "Power regimes", and "Democracy". However, focus is on policymakers and states as	"the states are the duty bearers" (FG1, CJ, 441-42)

Code	Definition	Application / Demarcation	Anchor quote
		actors and their function for FGHP.	
Multilateral organisations	This subcode compiles statements about the role of multilateral organisations as actors in a feminist global health policy.	Parallels exist to the code "Shift in funding". However, focus is multilateral organisations as actors and their function in FGHP, which extends beyond funding.	“I actually think everyone should be involved, and everyone can, and especially regarding institutions like UN Women and WHO. Those are the places where the resources sit as well. So, therefore, of course, ehm it should be also the responsibility of organisations like that to ehm to access their own resources, to then go on the national level and in the communities and work together.” (FG3, AN, 649-53)
Academia	This subcode compiles statements about the role of academia as an actor in a feminist global health policy.	Due to its educational focus, this subcode is related to the codes "ANreness raising" and "Education within the health system", and also to "Intersectionality" and "Quantitative & biomedical focus". However, focus is on academia and universities as actors and their function for FGHP.	“what you talked about this already, neoliberal university where we have to put like where the quantity of output is more important than the quality of it (ER nods). Then there is no focus on the impactful CJe, there's no focus on the process and only on the output.” (FG1, MM, 381-83)
Feminist economists	This subcode compiles statements about the role of	This code is related to the code "Shift in funding", but the focus is on feminist economists as	“but the lack of and to sort of equity informed, justice-informed social regulation of that

Code	Definition	Application / Demarcation	Anchor quote
	feminist economists as actors in a feminist global health policy.	actors, which implies more than funding. Moreover, the code "Shift in funding" includes further actors and aspects.	sector ehm has led to tremendous health inequalities, and I think that ehm feminist economics (CJ nods), with its emphasis on equity and justice, provides a very important antidote to that." (FG1, JC, 222-25)
Private sector	This subcode compiles statements about the role of the private sector as an actor in a feminist global health policy.	Due to the proximity to capitalism and privatisation, the code "Privatisation & capitalism" is closely linked to this code. However, the two codes are distinct as the code "Private sector" portrays a potential actor for a feminist global health policy, whereas the code "Privatisation & capitalism" depicts existing challenges.	"how are we keeping accountable corporations? Because those are the ones that are ruling the the world right now. And how are we talking about that? They have more power than most of the/ our government" (FG2, OLU, 662-65)
