# Supplementary material for: Towards a Feminist Global Health Policy: Power, intersectionality, and transformation
Source: PLOS Glob Public Health. 2024 Mar 7;4(3):e0002959. doi: 10.1371/journal.pgph.0002959 (PMC10919653; doi:10.1371/journal.pgph.0002959)
Supplement: Attachment — Submitted filename: Response to Reviewers.docx [file pgph.0002959.s004.docx]

**RESPONSE TO REVIEWERS**

Dear Editors,

We have re-submitted the manuscript “Towards a Feminist Global Health Policy: power, intersectionality, and transformation” to PLOS Global Public Health.

We thank the editors and reviewers for their thorough review and valuable comments. We have taken their recommendations into consideration and extensively edited the manuscript. With this letter, we briefly respond to each point raised by the editor and reviewers to better understand the changes that have been incorporated.

Kind regards,

Hannah Eger

on behalf of all co-authors

| **Comments by editors /**  **Journal requirements** | **Response by authors** |
| --- | --- |
| PLOS journals require all data underlying the findings described in the manuscript to be freely available to other researchers. | All required data has been uploaded and is freely available as Supporting Information. This entails three documents: (1) S1 Appendix: Guiding questions for facilitating the FGDs; (2) S2 Appendix: Transcripts of the FGDs; (3) S3 Appendix: Code system.  The data availability statement has been changed accordingly. |
| PLOS Licences and Copyright: potential copyright issues regarding Fig 3 | The corresponding author contacted the Editorial team to clarify the copyright for Fig 3: There should be no issue regarding copyright or licences. The authors used clip-art and icons from Microsoft Office, which are free to use without copyright information: <https://support.microsoft.com/en-au/office/insert-icons-in-microsoft-365-e2459f17-3996-4795-996e-b9a13486fa79>  This figure is also included in a similar way in the thesis publication of the corresponding author (it has been slightly modified for this submission). This means that the thesis’ publication might have to be mentioned as reference. |
| **Comments by reviewers** | **Response by authors** |
| ***General comments*** | |
| Provide a more in-depth overview of existing literature and efforts related to intersectional feminism in health. It is essential to delineate how their approach differs from or adds value to the existing body of knowledge. As it stands, it is unclear the added value of the small number of FGDs to the existing literature and frameworks, and what emerged from these FGDs that could have not been captured by a synthesis of the substantive amount of work already done in the field. This could enhance the manuscript's clarity and help readers understand the unique contributions being made. | The authors have included more information and references on intersectional feminism in health in the introduction (particularly in the sections “Understanding the intersectional landscape” and “The call for a Feminist Global Health Policy”), as well as in the discussion (see below). The unique contribution provided by this approach has been highlighted more extensively in the introduction and in the methodology chapter, by portraying the added value of the FGDs and by describing in-depth the methods and the reasoning behind this approach. |
| Some specific points/concepts/words seem sprinkled in the manuscript without a clear purpose. For instance, feminism, decoloniality, intersectionality, and equity are closely linked. However, for a peer-reviewed scientific paper (and not an editorial/opinion piece), you need to make sure what is the point you want to highlight. If you’re focusing on intersectional feminism, you must be intentional about using points on indigenous knowledge and elaborate on why you brought that point into the paper. | The language and terms used in the whole manuscript have been revised, particularly in the introduction. The authors incorporated changes to clarify the intention and meaning of words, concepts and terms used. To this end, definitions of the terms *intersectionality* and *health equity* were added to the introduction with further information to explain their purpose. The manuscript was checked and edited to avoid conflations of similar words and concepts. The reference to Indigenous knowledge in the introduction and its link to intersectionality has been edited for. |
| Concepts of health, healthcare, equity, and equality have been conflated throughout this manuscript. They must be clearly defined, and using them interchangeably must be avoided. |  |
| Explain what they exactly mean by concepts such as health equality or the fulfillment of bodily autonomy (bodily autonomy is more of a concept to consider and center, not a binary condition easily achieved, and so the wording when using such concepts must be very carefully examined). |  |
| Overall, the manuscript needs to be edited for language, accuracy, cohesion and flow. | The manuscript has been thoroughly revised in terms of language and content. The changes are presented below for each chapter, corresponding to the points being raised by the reviewers. Overall, certain points and aspects have been outlined more comprehensively, more references to existing literature have been included, the added value of this study has been emphasised and the discussion has been extended. The title of the manuscript was not changed after the changes have been incorporated. After taking account the reviewers’ comments and revising the manuscript, it has been shortened to optimise reader-friendliness. |
| In summary, while the manuscript presents an interesting perspective on intersectional feminism in global health, but a more comprehensive description of the methodology, discussion of existing literature, critiques, and potential strategies to avoid co-optation is necessary. Furthermore, I advise that the title is changed to better reflect what the paper presents. |  |
| ***Introduction*** | |
| The introduction section is a bit of patchwork, repeating exact keywords without a strong and cohesive flow to the points presented, missing a significant opportunity to present a strong argument for the need for this study. | The introduction section has been heavily edited. The language, with regard to words and flow, has been adjusted. Segments were shifted to allow for better comprehension and less replication. Relevant concepts were elaborated in more detail and existing literature, particularly on intersectional feminism in health, was more frequently referred to. By doing so, the unique contribution and added value of this study has been endorsed, which has been emphasised and explained specifically towards the end of the section. In the section introducing the research question, the objective of the study has been more comprehensively articulated as well as the reason for convening FGDs (more on FGDs below in the methodology section). |
| Essential to delineate how their approach differs from or adds value to the existing body of knowledge. As it stands, it is unclear the added value of the small number of FGDs to the existing literature and frameworks, and what emerged from these FGDs that could have not been captured by a synthesis of the substantive amount of work already done in the field. |  |
| Consider what the exact objective of the study was, why were the FGs convened, and continue to present those points. |  |
| Recommend that the authors consider revising the manuscript to provide a more in-depth overview of existing literature and efforts related to intersectional feminism in health. |  |
| Line 57: It is important to define intersectionality and, of course, important to note its roots in black feminist legal activism. | A comprehensive definition of intersectionality and its roots in black feminism, with reference to Kimberlé Crenshaw (among others), has been added. |
| Lines 66-67: The mention of GBV is abrupt and not followed up in the paragraph. | This paragraph has been completely changed. |
| Lines 76-79: Need references to the claims/points made. | Appropriate references have been added. |
| Lines 79-82: It would be more helpful if you elaborate on how these power regimes come into play, how intersectionality plays out, how these intertwined layers operate, and explain your points to set the scene for the reader. | In the edited section on intersectionality, the authors have elaborated on intertwined power regimes and the resulting forms of discrimination and outlined the added value of an intersectional approach. |
| Lines 83-84: This sentence is unnecessarily complex. Please rewrite to make your points clear. | This sentence has been changed accordingly. |
| Line 85 (and point repeated in Line 132): You introduce SDoH, but you have used political and structural DoH before this point. It is essential to 1. not conflate these concepts with each other, and 2. clearly define whichever one you want to use. | The SDoH, based on the model by Solar and Irwin, have been introduced and explained (section “Understanding the intersectional landscape”). In this regard, the authors are also referring to the structural determinants of health, as conceptualised in the model. |
| Lines 86-87: While correct, the point on indigenous knowledge seems to be mentioned suddenly and without proper introduction and follow-up. | This sentence has been edited and shifted to better align with the flow and to be set in context. |
| Lines 88 & 107: Decolonial might be a better word instead of decolonizing/decolonization. | The authors have adopted the recommendation. |
| Lines 99-100: Use appropriate references to highlight the increasing calls. | Appropriate references have been added. |
| ***Methodology*** | |
| *Data collection section:* | |
| It is unclear how FG2 and 3 differed. The composition of FGs is not mentioned. It is not clear how many people were in each FG. How the FGs convened in terms of moderation and/or data collection is unclear. What questions, themes, or discussion topics were introduced? How long did each FG meet for? How many times? You need to explicitly mention the gender, level of seniority, region, nationality, educational background, etc., of participants, in aggregate, to paint a clear picture of whose collective contributions led to the following sections while maintaining anonymity. | The authors have included an extensive description and explanation of the selection, composition and conduct of the FGDs in the methodology. This entails information on the number of participants and their background, their selection and the reason for purposive sampling, the non-response rate, as well as details on the procedure and content. Moreover, additional documents to ensure the traceability and transparency have been uploaded as Supporting Information, including the guiding questionnaire, transcripts (raw data) and the code system. Challenges and risks arising from this procedure are referred to in the methodology section and further discussed in the limitations section. |
| Why weren’t participants randomly mixed? This intentional differentiation risks creating hierarchies of knowledge. |  |
| Share information on procedure in FGDs. |  |
| The non-response must be elaborated. Does this mean the FGs were convened but participants did not attend? Does the number 11 include all who were invited to FGs, or all who showed up but didn’t contribute to the conversation? Or all who participated and contributed to the conversation? This is a significant point you need to explain. |  |
| *Role of primary researcher / facilitator and reflexivity:* | |
| Further, having a facilitator that ‘remained in the background’ seems an oxymoron. It is indicated that the lead researcher, a young white European woman with an advanced university degree was leading the conversation. How was she facilitating the FGDs yet remained in the background? Why weren’t someone external recruited for facilitating the discussion? There is an inherent power dynamic between the ‘researcher’ who is at the same time facilitator, and those who are participating, layered by other intersectional factors playing a role. | The role of the primary investigator and facilitator of the FGDs has been elaborated in great detail. Potential risks and remaining power hierarchies were identified and mentioned in the methodology section and are further discussed in the limitations section. The authors have decided to edit and enhance the reflexivity section as well as the limitations section, but not to merge these two, as reflexivity per se is not a limitation, but effects of the specific positionality can lead to limitations. The reflexivity section, as well as the limitations section, refer to the whole research process. Information on the co-authors and their involvement has been added to the reflexivity section. |
| The reflexivity section lacks any mention about researcher as facilitator in FGDs and inherent power dynamic. Though these are considered in the limitation section of the paper. Perhaps the sections on reflexivity and limitation should be revisited to consider what should go where, and whether they could be combined. |  |
| Enhance reflexivity, whole research process: reflexivity needs to also be applied in the process of developing the guiding questions, the selection of the participants, coding and analytical processes. Transcripts and audio recordings are not the ‘findings’, but the raw data. Content analysis is by nature a subjective process. Were the participants invited to provide input into the analytical process and the ‘findings’ following coding and analysis? |  |
| It is important to include other co-authors. If they are all FG members, this needs to be explicitly mentioned. If not, their positionality has also influenced the write-up of this research and is worth noting in the section. |  |
| Line 126: Needs a reference. | References have been added. |
| Lines 132-133: This sentence is unnecessarily complex. Please rewrite to make your points clear. | The sentence has been rewritten for improved comprehensibility. |
| Lines 140-141: Unclear what this sentence adds. | This sentence has been deleted. |
| Lines 143-145: “Drawing on constructionist ontology, postmodern epistemology, and interpretivist methodology, we used qualitative methods in this research.” I recommend starting the methods section with the most important piece of information: this is a qualitative study. Then bring up the abovementioned frameworks to elaborate on how they are applied to qualitative data interpretation. | The study design section has been amended accordingly, beginning by stating that this is a qualitative study. |
| Line 153: It is unclear how this section pertains to quality. And what quality? Quality of the overall project? Data quality? Data collection methods quality? Participant’s quality? Their selection criteria quality? | Information has been added to the quality criteria section to address the points raised. Moreover, the subheading “Quality criteria” has been deleted and content merged with the paragraphs above, since this information relates to proving that the study is a qualitative study. |
| Line 155: Unclear what you mean by “specific criteria for qualitative research were perceived as appropriate. “Maybe mention the “the following specific criteria.” |  |
| Line 161: Convened instead of conducted. | This has been changed accordingly. |
| Line 162: Online meetings platform, Zoom | This has been changed accordingly. |
| Line 163: what is the most appropriate composition? You need to elaborate on this appropriateness and its criteria. | Information has been provided to clarify what is understood as most appropriate composition. |
| Line 167: What do you mean by Majority World, given it has no reference yet is capitalized similarly to a particular name or concept? | The term Majority World has been deleted to avoid the introduction of too many concepts and because it does not add value to this section. |
| Lines 170-172: How was this done and achieved? | The sampling process has been described more comprehensively in the “Data collection” section to address this point. |
| Line 182: Elaborate how feminist principles come into play here. | The authors have outlined the conduct of the FGDs in greater detail and explained how feminist principles have been applied and what these constitute. |
| Line 185: A vibrant discussion… is not a scientific term and does not add to this paragraph either. | This sentence has been deleted. |
| Lines 204-205: Did they? I think this needs to be elaborated. | Information on the co-authors who were FGD participants has been included. |
| Lines 212-213: I’m unsure about including this in the positionality and reflexivity statement. | This sentence has been changed and partly deleted. |
| Figure 1: An arrow moving from deductive/inductive coding to the final code system is missing. | The arrow has been added. |
| Figure 2: You shouldn’t use abbreviations in figures without introducing them. AAAQ is an abbreviation. | All abbreviations have been written out in full. |
| ***Results*** | |
| As it stands, this manuscript is elevating certain important points, but does not really introduce a framework, nor tools to utilize/apply that framework. I believe this should be considered throughout the revision. | The intention of the framework, its use, added value and restrictions, have been addressed in the results section and extensively at the beginning of the discussion. The linkage between global, guiding principles and contextualised implementation has been referred to in detail throughout the discussion, based on the findings presented in the results section. The term “universal” has been replaced to emphasise that guiding principles form a common denominator, but that there are multiple approaches to achieving them. |
| While stating that “the analysis and findings are subjective and contextualised and should be interpreted accordingly”, the framework is presented as a ‘global health policy’ framework, something that can be perceived to serve as a universal one. This needs to be further discussed and elaborated. |  |
| The findings are summarizing some of the points, yet does not sufficiently contextualize the statements. For example, “The absence of regulation is fostered by a lack of political will to interfere.” (252). As the participants are based in different countries, it seems that the governments and the political will may be highly different in different countries. This is a bit too general conclusion. | The results section has been heavily edited to improve the flow as well as content. The structure has been adapted to emphasise and elaborate on important aspects. The whole section has been revised to better align FGD statements with the text. The authors made sure that conclusions derived from the results. To avoid making bold claims, some sentences have been deleted or have been contextualised. |
| While it is clear the authors intend to contribute to the discourse, they should refrain from making bold claims that can’t be directly linked to the focus groups. |  |
| Often, it was unclear how the authors moved from point A to point B and drew connections between quotes from the FGs and presented conclusions. |  |
| Figure 3 should come further in the manuscript, perhaps in the discussion alongside recommendations/Table 1, and should be explained and elaborated. | Figure 3 has been shifted to the discussion and is referred to more comprehensively in the text. |
| Line 291: Intersectionality: This section can be expanded on much more, given that it is a central theme in the manuscript title as well, but as it stands, it’s not at all informative, nor does it express how intersectionality is relevant to the solutions put forth by the authors. | The section on intersectionality has been heavily revised. It has been expanded to highlight its importance and changes have been made to include relevant aspects. The section of “Feminism itself” has been merged with the section on intersectionality, as the content was closely linked to intersectionality and aligns very well with this passage. |
| Line 322: “Feminism itself”. I believe the use of the word “itself” makes it sound conversational. “Feminism” should suffice to convey the message here. If changed, this should be fixed in Figures as well. |  |
| Line 332: Objectives and principles section: see overall comments at the beginning. This section starts by introducing the four principles but moves on not to dive deep into any of the principles; instead, it continues presenting results. It is a very piecemeal section, very long, and the absence of subheadings makes it very difficult to follow and understand. | The section on objectives and principles has been extensively edited. It has been separated into several subsections with subheadings. The new paragraphs include more comprehensive information, e.g. the four principles have been outlined in more detail. Statements from the FGDs were incorporated and referred to in the text. The adaptations intend to improve the flow and to provide a better understanding of the presented framework and its components. |
| Line 333: FGD is being used for the first time here. | The abbreviation FGD has been introduced earlier in the text. |
| Line 355: Democracy has not been discussed before here, except for one quote in line 256. If it is one of the four principles, it must be addressed much earlier in the paper. | Democracy and its relevance for this article has been introduced in the introduction. |
| Lines 367-380: A lot of repetition and not very cohesive. Language should be heavily edited to highlight the most important points and remove repetitions. | This section has been greatly edited, along with the adjustments described above. This includes the introduction of new subheadings, more concise language and less repetitions. |
| Line 387 & 526: UN Sustainable Development Goals 2030 (add reference) | References have been added. |
| Line 389-390: What do you mean by multiple oppressed individuals? As it stands now, it reads as multiple individuals, i.e., the plurality and number are important. I believe you intend to emphasize individuals experiencing multiple and intersecting layers of oppression. | The recommendation by the reviewer has been incorporated. |
| Line 391: Beginning of what? The policy-making process? | This sentence has been edited accordingly. |
| Lines 415-417: Another example of repetitive points that are not really coherent or placed well in the section. | In line with the thorough revision mentioned above, this section has been edited and adapted to the context. |
| Line 475-476: This paragraph is about academia, yet in the middle of the paragraph you add a very strong point about the health workforce without further elaboration. | The authors have added further elements to this paragraph. |
| Line 482: This claim needs references to some of these feminist economic theories or practices. | This sentence has been rewritten to clarify that this claim derives from the FGDs. References to feminist funding and economy have been added here as well as in the discussion. |
| ***Discussion*** | |
| The manuscript would benefit from examination of the critiques against feminist foreign policies and feminist development policies to date. Addressing these critiques is crucial to understanding the potential challenges the proposed feminist global health policy might face and how their new propose framework intends to tackle these concerns. Specifically, the authors should explore whether their approach sufficiently avoids the pitfalls of co-optation, dilution, and depoliticization of feminism. In the current global health landscape, which is often entrenched in colonial and patriarchal structures, it is essential to critically evaluate whether the proposed policy can indeed navigate these challenges effectively in the existing structures and institutions which are often rather hierarchical and patriarchal. | The authors have incorporated this recommendation by adding a comprehensive section in the discussion. The criticism of feminist foreign and development policies are presented and explained, in particular with regard to the risks of co-optation, dilution and depoliticisation. Moreover, the authors have added a section on how to avoid and encounter these pitfalls in implementing feminist global health policy. |
| The authors must be very cautious when drawing a conclusion about the robustness and validity of a concept. As this manuscript is submitted for peer review and conducted according to a specific qualitative methodology, validity should not be claimed based on similarities with other published work that have not been conducted following similar or other scientific methodology. The CFFP paper is a desk review type of publication, and the Davies et al. paper is a viewpoint. Both are great additions to the discourse but cannot be used in a point about the validity of a framework introduced in this paper. (Line 548-549) | This section has been revisited and the authors have placed greater attention on not drawing preliminary conclusions about the robustness and validity of the FGHP framework. |
| *On limitations:* FGDs are only conducted in English, which indicates that participants were having a rather advanced level of fluency of and comfort with English language, further indicating an advanced level of literacy for non-native English speakers, which already introduces some biases in the sample (...) The authors mention the limitation of the process, but does not reflect more on the influence on the outcome. | The limitation section has been edited. More details have been provided and greater emphasis has been put on the consequences of the limitations. Furthermore, the limitation section has been revised with regard to the reflection section in the methodology chapter. |
| Line 566: FGD is used again. | The abbreviation “FGD” has been introduced earlier in the manuscript. |
| Line 583: Respected or implemented? | The authors have changed the term according to the comment. |
| Line 600: Terminate? Or transform? | The authors have changed the term according to the comment. |
| Lines 657-658: Deeply embedded or informed by? | The authors have changed the term according to the comment. |
| Line 683: It would be great to elaborate on the recommendations and briefly describe where each recommendation is drawn from - this could happen earlier in the discussion section. For example: A, B, and C, raised by the FG and the literature (add references), led us to make this recommendation: X | The Recommendations Table has been amended with regard to listing more actionable recommendations. However, the authors have refrained from elaborating on each recommendation in the discussion, to keep the manuscript shorter and avoid repetition. |
| Table 1: Recommendations are not actionable, e.g., Adopt more holistic approaches within global health research. |  |
| Table 1: Research column, second point: extend, not extent. |  |
